# Long-Term Prosthesis Survival After Total Hip Arthroplasty in Young Women with Developmental Dysplasia of the Hip: A Narrative Review of Instability, Revision Risk, and Reproductive Health Considerations

**DOI:** 10.3390/jcm15187220

**Published:** 2026-09-17

**Authors:** Man Hung, Annabella Jensen, Isabella Strickler, Sharon Vu, Avianna Arapovic, Mouhanad El-Othmani

**Affiliations:** 1 College of Dental Medicine, Roseman University of Health Sciences, South Jordan, UT 84095, USA; 2 Division of Public Health, University of Utah, Salt Lake City, UT 84108, USA; 3 William Beaumont School of Medicine, Oakland University, Rochester, MI 48309, USA; 4 School of Medicine, Boston University, Boston, MA 02118, USA; 5 Lake Erie College of Osteopathic Medicine, Erie, PA 16509, USA; 6 Department of Orthopaedic Surgery, Corewell Health, Royal Oak, MI 48073, USA; 7 Warren Alpert Medical School, Brown University, Providence, RI 02903, USA

**Keywords:** total hip arthroplasty, developmental dysplasia of the hip, young women, prosthesis longevity, instability

## Abstract

**Background/Objectives:** Total hip arthroplasty (THA) reliably improves pain and function, but implant longevity in women aged ≤50 years is influenced by longer duration of implant use and distinct failure mechanisms. Developmental dysplasia of the hip (DDH) is a common underlying diagnosis among patients undergoing THA at younger ages and may increase instability risk, while pregnancy after THA remains an important but understudied consideration. **Methods:** A narrative review of contemporary studies published from January 2000 through January 2026, supplemented by seminal older studies when directly relevant, was conducted. Evidence was synthesized by clinical mechanism and endpoint, with an emphasis on instability, revision, and pregnancy-related outcomes. **Results:** Outcomes were generally favorable in young women undergoing THA. DDH-related anatomic complexity may increase instability risk, but this risk appears highly modifiable through contemporary reconstructive and implant strategies. Pregnancy after THA was not consistently associated with increased dislocation or revision, though DDH-specific data are sparse. **Conclusions:** In women aged ≤50 years, THA longevity is shaped by both prevention of early instability-related revision and preservation of durable long-term fixation. DDH should be viewed as a reconstructive phenotype requiring stability-focused surgical planning and counseling. Future research should standardize instability reporting and evaluate the interactions among DDH severity, implant strategy, and long-term revision risk.

## 1. Introduction

Total hip arthroplasty (THA) is among the most successful operations in modern medicine, reliably improving pain and function across a wide range of hip diseases [1]. Yet the stakes and the failure modes look different in women undergoing THA at age 50 years or younger. This group faces a substantially longer lifetime exposure to an implant, higher cumulative mechanical demand, and a greater likelihood that complications, particularly instability, will lead to reoperation and revision [2]. Contemporary series suggest excellent short- to mid-term implant longevity even in younger cohorts, but the clinical problem for women ≤ 50 is less about whether a modern THA “works” at 5–10 years and more about what fails first, why it fails, and how early failure alters lifetime revision burden [2].

A major reason women present for THA decades earlier than the “typical” osteoarthritis (OA) population is developmental dysplasia of the hip (DDH) [2]. DDH is strongly female-predominant (often reported as several-fold more common in women than men) [3], and many dysplastic hips develop symptomatic degeneration early in adulthood [4]. Importantly, DDH is not simply “OA at a younger age.” It is a distinct structural phenotype characterized by variable acetabular deficiency, altered femoral morphology and version, and soft-tissue adaptations that can persist even in “milder” adult dysplasia [5,6]. In a recent study of patients undergoing THA under age 50, dysplasia features were strikingly common, depending on the radiographic definition used; over 40% met a dysplasia criterion, highlighting how frequently dysplastic morphology is present in so-called “young OA” THA cohorts [7]. This prevalence makes DDH-centered risk modeling and counseling particularly relevant to women ≤ 50 years.

From a reconstructive standpoint, DDH creates a set of interlocking challenges that directly relate to the outcomes patients and surgeons fear most—instability and revision [5,6]. The dysplastic acetabulum may be shallow and deficient, driving difficult choices about whether to reconstruct at the anatomic hip center, use a high hip center, or augment deficient bone; the femur may exhibit abnormal version, narrow canals, and altered offset; and the combined anteversion “envelope” may be harder to optimize [5,6]. Contemporary reviews of THA in DDH emphasize that these morphological alterations influence component placement strategy, restoration of offset and leg length, and soft-tissue tensioning—all determinants of impingement, instability, and long-term mechanics [5,6]. Consequently, comparing DDH with non-DDH diagnoses is not merely comparing “etiologies”; it is comparing different anatomic starting points and different technical solutions, each with its own failure pathways [5,6].

Despite decades of work on THA outcomes in dysplasia, the literature still leaves important uncertainties precisely where young women need clarity. Meta-analytic evidence suggests that revision may be higher after THA for DDH than for OA, although estimates are sensitive to confounding by age, activity level, dysplasia severity, and implant era; notably, some syntheses find revision differences without consistent differences in dislocation across pooled populations, underscoring how heterogeneous the underlying data are [8]. Meanwhile, registry-based work in younger patients broadly indicates that revision risk depends on modifiable factors such as surgical approach, head size, and bearing choice—variables that intersect directly with instability prevention strategies [9,10]. At the population level, instability is a common indication for reintervention and is associated with recurrent events and substantial patient burden [9,10]. For women ≤ 50 years, who often have decades of anticipated implant use, an early instability event can be more than an acute complication: it can initiate a sequence of liner exchange, constrained constructs, dual-mobility conversion, or full revision, each potentially trading one risk for another and shaping lifetime outcomes [11].

Critically, much of the existing DDH versus non-DDH literature is not tailored to the questions young women actually ask in clinics. First, many analyses pool men and women across wide age ranges, obscuring sex- and age-specific mechanisms. Second, DDH is frequently treated as a binary label rather than a spectrum, and dysplasia severity classifications (e.g., Crowe or Hartofilakidis) are inconsistently reported, limiting phenotype-specific inference. Third, implant-era effects matter: conclusions drawn from legacy polyethylene, older fixation strategies, or historical component designs may not translate cleanly to modern bearings and contemporary stability options. Finally, and most relevant to young women, the literature rarely integrates reproductive-life exposures into outcome interpretation, counseling, or research design.

The intersection of THA and pregnancy is no longer a curiosity. A nontrivial number of women undergo THA in their 20s, 30s, and 40s, often for DDH, making pregnancy after THA a foreseeable scenario [12,13]. The best-known early large series of young women after THA documented successful pregnancies and a mix of vaginal and cesarean deliveries, while also illustrating how limited the evidence base was even in a high-volume setting [12]. More recent reviews emphasize that current knowledge remains fragmented, derived from a small number of observational studies with heterogeneous outcomes and limited diagnosis-stratified reporting; a 2024 systematic review explicitly highlights the inadequacy of the evidence base and the need for clearer, multidisciplinary interpretation of maternal and obstetric considerations [14]. Population-based data also raise the possibility of higher rates of selected obstetric complications among women with prior THA, though findings differ across study designs and settings [15]. Yet DDH-specific pregnancy counseling is precisely where anatomy, biomechanics, and life-course change plausibly intersect, and where the absence of a consolidated, diagnosis-aware synthesis leaves clinicians without a clear framework for counseling and leaves patients navigating uncertainty.

Given these gaps, there is a clear need for a focused synthesis that is (1) woman-centered, (2) mechanistically organized around instability and revision, and (3) explicit about how DDH differs from non-DDH diagnoses in reconstructive decisions and failure pathways, while also acknowledging where evidence is limited and where consensus-based counseling must be transparently labeled as such. Narrative reviews are well suited to this task when the goal is to integrate registry data, cohort studies, surgical technique literature, biomechanical concepts, and emerging obstetric considerations into a clinically usable framework, provided that the process is transparent and the claims are anchored to the strength and type of available evidence.

## 2. Objectives of This Review

Accordingly, this narrative review synthesizes contemporary evidence on (1) total hip arthroplasty outcomes in women aged ≤50 years, comparing developmental dysplasia of the hip and non-dysplastic diagnoses and emphasizing instability and revision mechanisms, including cause-specific revision where available; (2) modifiable surgical and implant-related factors relevant to preventing instability and revision in both dysplastic and non-dysplastic anatomy; and (3) the limited literature on pregnancy and childbirth after total hip arthroplasty, culminating in a practical counseling framework and a prioritized research agenda.

By organizing the evidence around why failures occur, rather than solely how often they occur, this review aims to strengthen patient counseling, inform surgical planning for young women, particularly those with developmental dysplasia of the hip, and identify the most actionable studies needed to close persistent evidence gaps.

## 3. Methods

### 3.1. Design

This narrative review focused on prosthesis longevity in women aged ≤50 years undergoing primary THA, comparing DDH with non-DDH diagnoses [16]. The goal was not to exhaustively capture every publication or statistically pool estimates, but to produce a clinically useful, mechanism-oriented synthesis centered on instability and revision pathways and to identify modifiable surgical and implant factors that may reduce instability-driven revision. No formal risk-of-bias assessment was performed because the objective was a mechanism-oriented narrative synthesis rather than quantitative evidence appraisal.

### 3.2. Scope and Key Definitions

For this review, “young” was defined as ≤50 years at the time of primary THA. When studies reported subgroups using age cutoffs between 40 and 45 years, those data were highlighted as a focused lens within the broader ≤ 50 population. DDH was accepted as defined by each study (e.g., clinical diagnosis, radiographic dysplasia, or registry coding); when severity classification (e.g., Crowe or Hartofilakidis) was reported, it was extracted to contextualize outcomes. “Non-DDH” comparators included primary osteoarthritis and other non-dysplastic etiologies as defined by the study (e.g., avascular necrosis, inflammatory arthritis, post-traumatic degeneration). The primary outcomes of interest were instability/dislocation and revision (all-cause and cause-specific, particularly instability-related revision). Pregnancy and childbirth after THA were treated as a dedicated topic because they are highly relevant to young women and are incompletely synthesized in the DDH-specific literature.

### 3.3. Literature Identification

We used a targeted and iterative search strategy designed to capture (1) influential and contemporary evidence on DDH versus non-DDH THA outcomes in younger patients, and (2) the smaller body of work addressing pregnancy/childbirth after THA. Searches were conducted in major biomedical databases (PubMed, Scopus, Web of Science, and the Cochrane Library), with the final search performed in January 2026 using a primary search window from January 2000 through January 2026 to emphasize modern implant eras. Seminal older studies of THA for dysplasia were also included when they directly informed reconstruction challenges or long-term failure patterns.

Search terms combined concepts for THA (“total hip arthroplasty,” “total hip replacement”), DDH (“developmental dysplasia,” dysplasia, DDH, Crowe, Hartofilakidis), age/sex (women, female, young, <50), and outcomes (instability, dislocation, revision, survivorship, reoperation). A representative search strategy combined these concepts using Boolean operators: (“total hip arthroplasty” OR “total hip replacement”) AND (“developmental dysplasia” OR dysplasia OR DDH OR Crowe OR Hartofilakidis) AND (women OR female OR young OR < 50) AND (instability OR dislocation OR revision OR survivorship OR reoperation). Search syntax was adapted as appropriate for each database. Because pregnancy-related evidence is often not captured by standard arthroplasty keywords, we also performed a dedicated search combining THA terms with pregnancy/childbirth/postpartum terms (pregnancy, delivery, cesarean, postpartum). To capture key papers that may not surface through database queries alone, we also screened reference lists of highly relevant reviews and sentinel studies and used forward citation checks to identify subsequent related work. Because this was a targeted narrative review rather than a systematic review, formal record counts and PRISMA-style screening were not performed.

### 3.4. Study Inclusion

Because this is a narrative review, we used relevance-based inclusion rather than exhaustive screening. Studies were prioritized if they (1) included women aged ≤50 years, (2) reported outcomes for DDH and a non-DDH comparator, and (3) reported at least one primary endpoint: instability/dislocation and/or revision.

Large registries and well-designed cohorts were used to summarize rates and predictors of revision and instability, while smaller cohorts and biomechanical/technique-focused papers were included when they clarified reconstructive mechanisms relevant to DDH. Studies of pregnancy and childbirth after THA were included even when diagnosis-stratified DDH data were limited, but they were explicitly labeled as limited evidence where appropriate.

Evidence from studies directly matching the target population (women aged ≤50 years) and including a DDH versus non-DDH comparison was prioritized as the core evidence base. Studies including broader age ranges, populations that were predominantly, but not exclusively, female, or DDH-only cohorts were considered supportive evidence when they provided clinically relevant information regarding reconstructive factors, instability mechanisms, or long-term failure patterns. These studies were interpreted in the context of their population limitations and were not considered equivalent to evidence directly matching the target population.

### 3.5. Data Extraction

From each included study, we extracted a focused set of variables necessary to support a mechanism-oriented synthesis and clinically meaningful comparisons. These included key elements of the study context, such as design (registry or cohort), country, study period, sample size, age definition, duration of follow-up, and implant era, when identifiable. We also recorded population and diagnostic characteristics, including how developmental dysplasia of the hip was defined, whether dysplasia severity was reported, and the composition of the non-dysplastic comparator group.

When available, we captured relevant surgical and implant features, including surgical approach, reconstruction strategy (for example, hip center strategy in dysplasia), fixation method, femoral head size, use of dual-mobility constructs, and bearing surface. Outcomes of interest included definitions and rates of instability or dislocation, overall revision rates, causes of revision, particularly those related to instability, and reported effect estimates for predictors where provided. Finally, for studies addressing pregnancy, we extracted the number of pregnancies, timing relative to total hip arthroplasty, hip-related outcomes during and after pregnancy, and any delivery-related considerations described.

The unit of analysis (hip vs. patient) was extracted and retained as reported in the original study, and no conversion between units was attempted. Implant-level outcomes, including revision, dislocation, and survivorship, were interpreted as hip-based when reported as such, whereas patient-reported and pregnancy-related outcomes were interpreted at the patient level.

### 3.6. Evidence Synthesis

Evidence was synthesized narratively and organized by clinical mechanism and endpoint, rather than chronologically. We first summarized the study landscape (the types of evidence available for women aged ≤50 years with DDH), then synthesized findings in three sections: (1) instability/dislocation patterns in DDH versus non-DDH, (2) revision outcomes with emphasis on instability-driven revision, and (3) modifiable surgical and implant factors linked to instability and revision risk. The pregnancy/childbirth literature was synthesized in a dedicated section and translated into a practical counseling box, with a careful distinction between findings supported by direct evidence and those that are best interpreted as consensus-based guidance in areas where evidence remains sparse.

Because the included studies varied widely in their definitions, implant eras, and reporting, we did not perform a meta-analysis. Instead, we emphasized consistency in the direction of findings across higher-quality sources (particularly registries and robust cohorts), highlighted where findings were mixed or potentially confounded (e.g., implant-era effects, diagnosis misclassification, confounding by indication for dual-mobility use), and transparently noted where evidence was limited.

## 4. Search Results

### 4.1. Search Yield

The targeted search strategy identified a heterogeneous but thematically coherent body of literature addressing THA outcomes in younger patients, with a strong female predominance and a substantial representation of DDH. Included studies comprised large national and multinational arthroplasty registries, regional registries, institutional registries, retrospective and prospective cohort studies, and focused single-center case series. Together, these sources captured outcomes across the full spectrum of DDH severity (Crowe I–IV), as well as non-dysplastic comparator diagnoses such as primary osteoarthritis, avascular necrosis, post-traumatic arthritis, and inflammatory arthritis.

Most studies did not restrict inclusion strictly to women aged ≤50 years, but women consistently represented the majority of DDH cohorts, and younger age strata (often <65 years) were commonly reported. Several registry-based analyses provided age-stratified outcomes that allowed inference relevant to the population aged ≤50 years, while institutional series frequently focused on younger, more complex DDH reconstructions, particularly Crowe III–IV hips. Studies addressing pregnancy and childbirth after THA were comparatively few and largely observational, with limited diagnosis-specific stratification.

Across the literature, outcomes were variably reported as hip-based (e.g., revision per hip, dislocation per hip) or patient-based (e.g., complications per patient, PROMs per patient), particularly in registry studies and bilateral cases. Where ambiguity existed, outcomes were interpreted according to the reporting unit used by the original study and were labeled accordingly in synthesis tables.

Overall, the included evidence captures: (1) instability and dislocation risk across DDH and non-DDH diagnoses, (2) revision rates and mechanisms over short-, mid-, and long-term follow-up, (3) the influence of reconstructive strategies and implant-era factors, and (4) limited but clinically relevant data on pregnancy after THA in young women. The resulting body of evidence is well suited for a mechanism-oriented narrative synthesis, while also revealing consistent limitations in reporting granularity.

### 4.2. Characteristics of Included Studies

The included studies comprised a mix of population-based registry analyses and single-center retrospective and prospective cohort studies, predominantly from Europe, East Asia, and North America (Table 1 and Table 2). Most investigations focused on primary THA performed for developmental DDH, with several large registry studies including non-DDH comparator groups, most commonly primary osteoarthritis. Sample sizes ranged from small institutional series of fewer than 50 hips to national registry cohorts including tens of thousands of procedures. Follow-up duration varied widely, from short-term perioperative assessments to long-term survivorship analyses extending beyond 20 years. Across studies, outcomes were most frequently reported at the hip level, and detailed reporting of DDH severity, implant constructs, and instability definitions was inconsistent.

### 4.3. Evidence Gaps Observed

Several important evidence gaps emerged consistently across the included studies. First, DDH severity is frequently underreported or unavailable, particularly in large registry datasets, where diagnosis is typically based on surgeon-reported or administrative coding without radiographic confirmation. As a result, Crowe or Hartofilakidis classifications are absent in many of the largest and most influential population-level analyses, limiting phenotype-specific risk stratification.

Second, sex- and age-specific reporting remains limited. Although women comprise the majority of DDH cohorts and younger patients are commonly included, relatively few studies isolate outcomes specifically for women aged ≤50 years. Instead, inference often relies on subgroup analyses, age categories that extend beyond 50 years, or predominantly female cohorts without formal sex-stratified reporting.

Third, instability is inconsistently defined and reported. Some studies report any dislocation event, others recurrent instability, and others revision for instability only. Timing of instability (early vs. late) is variably captured, and cause-specific revision coding is incomplete in many registries. This heterogeneity complicates direct comparison of instability-driven failure pathways across studies and implant eras.

Fourth, implant-era and construct details are often incompletely described in registry studies. Key modifiers such as head size, liner type, dual-mobility use, offset restoration, and combined-version strategy are frequently unavailable, introducing confounding by indication and limiting causal inference regarding modifiable surgical factors.

Finally, pregnancy and childbirth after THA remain sparsely studied, particularly in DDH-specific populations. Existing studies are small, observational, and heterogeneous, with limited linkage between arthroplasty variables, dysplasia severity, pregnancy timing, delivery mode, and hip-specific outcomes. No study systematically integrates obstetric and arthroplasty registries, which represents a major gap for counseling young women with DDH.

### 4.4. Summary of Endpoints

Across diagnostic groups, the most consistently available endpoints were all-cause revision, instability/dislocation, and implant survivorship, particularly in registry-based studies that included broad age ranges and long-term follow-up. These outcomes were generally reported at the hip level, with time-to-event analyses using Kaplan–Meier or competing-risk methods.

For DDH cohorts, especially in institutional series enriched for younger patients and severe dysplasia (Crowe III–IV), additional endpoints included cause-specific revision, dislocation timing, technical complications (e.g., intraoperative fracture, nerve palsy), and radiographic outcomes related to hip-center position, offset, and component positioning. Functional outcomes (HHS, WOMAC, HOOS, mHHS) were frequently reported but inconsistently stratified by age or severity.

In non-DDH comparator groups, outcomes were dominated by registry-based revision and complication metrics, with fewer studies reporting detailed reconstructive or biomechanical endpoints. Comparative analyses between DDH and non-DDH diagnoses most commonly focused on revision risk, early dislocation, and short-term complications, with fewer studies addressing long-term instability mechanisms.

Age-stratified outcomes specific to women ≤ 50 years were available primarily through subgroup analyses within larger datasets and through younger-skewed DDH case series. Pregnancy-related endpoints, including hip symptoms during pregnancy, delivery mode, and postpartum hip outcomes, were reported at the patient level in a small number of studies and were rarely linked to DDH severity or implant characteristics.

Taken together, the available endpoints support a robust qualitative synthesis of instability- and revision-related mechanisms in young women with DDH, while underscoring the need for future studies that integrate radiographic severity, implant constructs, sex-specific analyses, and reproductive-life exposures.

## 5. Instability/Dislocation

### 5.1. How Instability/Dislocation Is Defined Across Studies

Across studies examining THA outcomes in younger patients, instability and dislocation were defined heterogeneously, reflecting differences in study design, data source, and endpoint focus [63]. Most institutional cohorts and single-center series defined instability as any postoperative hip dislocation, typically confirmed clinically and radiographically, regardless of whether the event was isolated or recurrent [20]. In these studies, outcomes were generally reported at the hip level, particularly when bilateral cases were included [64].

In contrast, registry-based studies most often captured revision for instability or dislocation rather than dislocation events themselves. As a result, registry analyses frequently underestimate the true incidence of instability by excluding non-revised dislocation events, closed reductions, or recurrent subluxation managed nonoperatively [65]. Timing definitions also varied: some registries distinguished early (e.g., ≤6 months) versus late revisions, while others reported cumulative revision risk over the full follow-up period without specifying the temporal distribution of instability events [66].

Few studies explicitly differentiated a single dislocation from recurrent instability, and only a minority reported dislocation severity, direction, or mechanism [20,64]. Importantly, several studies that focused on reconstructive technique or implant survivorship reported no instability events, but often did so without standardized definitions or adjudication processes [26,62]. Taken together, these inconsistencies limit direct cross-study comparisons and emphasize the need to interpret instability outcomes within the context of each study’s design and reporting framework [63,65].

### 5.2. Frequency and Timing

Early instability. When timing was reported, instability in younger women most commonly occurred in the early postoperative period, typically within the first 3–12 months after THA [64,67]. Large registry studies demonstrated that patients with DDH experienced a disproportionately higher risk of early revision for dislocation than patients with non-dysplastic diagnoses, even after adjustment for age and sex [17,21]. This early excess risk may reflect the complex anatomy and reconstructive challenges inherent to dysplastic hips, including altered acetabular orientation, femoral version abnormalities, and difficulty restoring offset and soft-tissue tension [64,68]. Institutional series focusing on severe DDH (Crowe III–IV) reported variable early dislocation rates, ranging from 0% to approximately 10–12%, depending on implant strategy, head size, and reconstructive technique [43,61,64]. Studies that emphasized restoration of the anatomic hip center, careful version control, and stability-focused constructs frequently reported no early dislocations, whereas cohorts with smaller femoral head sizes or less optimized offset restoration demonstrated higher early instability rates [43,61,69]. Notably, early instability was uncommon in non-DDH comparator groups within similar age ranges when contemporary implants and techniques were used [21,70]. 

Late and recurrent instability. Late instability and recurrent dislocation were less frequently reported, particularly in younger cohorts, largely because many studies lacked long-term follow-up or focused on revision rather than dislocation events [21,67]. Registry data suggest that while DDH is associated with a higher early risk of revision for dislocation, this excess risk does not consistently persist into the mid- or long-term once early failures are excluded [17,21]. In long-term institutional series of DDH treated with modern cementless fixation, late dislocation was uncommon, and most instability-related revisions occurred within the first few postoperative years [61,62]. Recurrent instability was rarely analyzed as a distinct endpoint. When reported, it was typically embedded within broader revision or reoperation outcomes, making it difficult to separate the natural history of instability from treatment effects such as liner exchange, constrained components, or conversion to dual-mobility constructs. This lack of granularity represents a major gap in understanding how early instability events translate into long-term revision risk in young women [65,67].

### 5.3. DDH Versus Non-DDH Comparisons

Direct comparisons between DDH and non-DDH diagnoses consistently indicate that DDH is associated with a higher risk of instability-related failure, particularly in the early postoperative period [17,21]. Registry-based analyses demonstrate significantly increased relative risks or hazard ratios for early revision due to dislocation in DDH compared with primary osteoarthritis, even after adjustment for demographic factors and fixation method [17,21]. These findings are echoed by large administrative database studies that report higher short-term dislocation rates in younger DDH patients than in age-matched non-DDH cohorts [70].

However, the magnitude of this risk difference is strongly influenced by implant era and construct selection. Institutional cohorts employing larger femoral heads, meticulous version control, restoration of offset, and stability-enhancing constructs, most notably dual-mobility cups, often report instability rates in DDH that approach or equal those of non-dysplastic hips. In contrast, studies using smaller head sizes, conventional fixed-bearing constructs, or less standardized reconstructive strategies tend to report higher instability rates in DDH [43,66,69].

Importantly, many studies lack detailed reporting of DDH severity, limiting the ability to determine whether observed differences are driven by dysplasia itself or by the disproportionate contribution of severe (Crowe III–IV) hips. Moreover, non-DDH comparator groups are heterogeneous, often combining primary osteoarthritis with avascular necrosis, inflammatory arthritis, or post-traumatic diagnoses, each with distinct instability profiles [21,64].

Overall, the comparative evidence suggests that instability risk in DDH is closely linked to reconstructive factors, including component positioning, version management, offset restoration, and implant selection. Table 3, Table 4 and Table 5 summarize instability definitions, timing, rates, effect estimates, and key risk modifiers across studies, highlighting both the consistent early vulnerability of dysplastic hips and the substantial opportunity for risk mitigation through contemporary surgical and implant strategies [43,68]. Factors associated with instability risk after THA in patients with DDH include dysplasia severity, component positioning, femoral version, offset restoration, femoral head size, dual-mobility constructs, and reconstructive strategy (Table 4).

#### Modifiers and Predictors

Approach. Surgical approach was variably reported and inconsistently analyzed as an independent predictor of instability [65,73]. Registry-based studies frequently recorded approach but rarely demonstrated a robust, diagnosis-specific association between approach and instability-related revision after adjustment for confounders [73,74]. In institutional cohorts, the posterolateral approach was most commonly used, including in series of severe dysplasia, without uniformly high instability rates [43,62]. More recent single-center studies using a standardized direct anterior or posterolateral approach reported low dislocation rates in both DDH and non-DDH cohorts, suggesting that approach alone is unlikely to be the dominant driver of instability risk in young women. Instead, approach appears to act as a contextual factor whose effect is mediated by component positioning, soft-tissue management, and surgeon experience [65].

Component orientation, version management, and offset restoration. Component orientation and restoration of native hip biomechanics emerged as the most consistently cited modifiable predictors of instability across study designs [64,65,75]. Dysplastic anatomy introduces substantial challenges related to acetabular version, femoral anteversion, and offset restoration, particularly in Crowe III–IV hips [64,68,75]. Institutional series emphasized the importance of placing the acetabular component at or near the anatomic hip center, optimizing combined anteversion, and avoiding excessive limb lengthening [61,62,75]. Studies reporting meticulous version control, often facilitated by modular femoral stems, navigation, or robotic assistance, demonstrated very low instability rates despite severe dysplasia [43,66,76]. Conversely, registry studies, which lack radiographic detail, consistently showed higher early instability-related revision rates in DDH [17,21], underscoring that inadequate biomechanical restoration rather than diagnosis alone likely underlies much of the observed risk. Offset restoration and soft-tissue tensioning were frequently discussed qualitatively but rarely quantified, representing a notable gap in the literature [65].

Head size and liner strategy. Femoral head size was one of the most consistently reported implant-related factors associated with instability [63,65]. Earlier-era studies employing smaller head sizes (≤28 mm) reported higher dislocation rates in DDH cohorts, whereas more contemporary series using 32–36 mm heads demonstrated markedly lower instability [61,77]. Several registry analyses identified small head size as an independent predictor of revision for dislocation, regardless of underlying diagnosis [10,73]. Liner strategy was less consistently described. Fixed-bearing polyethylene liners predominated across most studies, with limited reporting on constraint or elevated-rim liners. Where reported, instability events were more common in constructs with smaller effective head sizes, reinforcing the importance of head–neck ratio and jump distance in dysplastic hips [63,65].

Dual-mobility use. Dual-mobility (DM) constructs were increasingly represented in recent institutional cohorts and were consistently associated with very low or absent instability rates in DDH populations, including high-risk Crowe III–IV hips [43,66,69]. However, DM use was almost exclusively reported in non-randomized settings, often selected specifically for patients perceived to be at higher risk of instability [66].

As a result, interpretation of DM effectiveness is complicated by confounding by indication. While the near-zero dislocation rates observed in DM cohorts are compelling, they cannot be directly compared with fixed-bearing constructs without adjustment for underlying risk. Moreover, registry datasets often lack the granularity to identify DM use reliably, limiting large-scale comparative analyses [21,73]. Despite these limitations, the consistency of findings across modern cohorts suggests that DM constructs may represent an effective instability-mitigation strategy in young women with dysplasia when used judiciously [63,66,69].

### 5.4. Practical Takeaway

Associations between modifiable surgical and implant factors and instability should be interpreted cautiously because the available evidence is predominantly observational and may be influenced by patient selection, surgeon preference, implant era, and confounding by indication. Across studies, several themes emerge with relative consistency. First, DDH, particularly severe dysplasia, is associated with a higher early risk of instability, especially when assessed through revision-based endpoints [17,21,64]. Second, this risk is highly modifiable: meticulous component positioning, restoration of offset, optimization of version, and use of larger effective head sizes substantially mitigate instability risk [43,65,75]. Third, contemporary series demonstrate that instability rates in DDH can approach those of non-dysplastic hips when modern techniques and implants are employed [43,66,69].

In contrast, evidence regarding the independent effects of surgical approach, liner constraint, and specific stem designs remains mixed, largely due to inconsistent reporting and limited adjustment for confounders. Overall, instability in young women should be viewed not solely as an inherent consequence of DDH but also as a function of reconstructive strategy and implant selection [65,73].

## 6. Revision Outcomes and Mechanisms

### 6.1. How Revision Is Defined and Reported

Revision outcomes were defined heterogeneously across studies, with important distinctions between registry-based and institutional cohorts. Registries uniformly defined revision as removal or exchange of one or more implant components, enabling standardized capture of all-cause and cause-specific revision but excluding reoperations without component exchange. Consequently, events such as closed reductions for dislocation, liner dissociation managed nonoperatively, or soft-tissue procedures were not captured [21,73,78].

Institutional cohorts often used broader definitions, occasionally including liner exchanges, head exchanges, or reoperations for instability, though definitions were not always explicit. Outcomes were most commonly reported at the hip level, particularly in studies including bilateral procedures [43,62,79]. These differences in definition and ascertainment substantially influence reported revision rates and must be considered when making comparisons across study designs [65,78].

### 6.2. All-Cause Revision: DDH Versus Non-DDH

Across large registry studies, all-cause revision rates in young women with DDH were similar to or modestly higher than those observed in non-DDH cohorts, particularly cohorts with primary osteoarthritis. When differences were observed, they were most pronounced in the early postoperative period and attenuated with longer follow-up [17,21]. Several registries reported comparable 5- to 10-year survivorship between DDH and non-DDH diagnoses after adjustment for age and sex [17,21].

Institutional cohorts demonstrated wide variability in revision rates, reflecting differences in DDH severity, implant era, and reconstructive complexity. In severe DDH (Crowe III–IV), revisions were more commonly related to aseptic loosening, osteolysis, or mechanical complications than to instability [61,62]. Importantly, instability-related revision accounted for a small proportion of all revisions in both DDH and non-DDH cohorts, even when early dislocation risk was elevated [17,21,78].

Supplementary Tables S1–S3 summarize study characteristics, instability outcomes, and revision patterns across the included studies, and highlight that although DDH increases reconstructive complexity, long-term implant survival in young women is often comparable to that in non-dysplastic hips when contemporary techniques are used.

Cause-specific revision coding was also inconsistent. Registry datasets depend on surgeon-reported primary reasons for revision, which may incompletely capture multifactorial failure mechanisms or misclassify events such as instability-associated loosening or liner-related complications. Accordingly, when cause attribution was incomplete or variably defined, revision mechanisms should be interpreted cautiously, and estimates should be considered approximate rather than definitive.

### 6.3. Cause-Specific Revision

Instability-driven revision. Across both registry and institutional studies, instability-related revision represented a minority of all revisions in women aged ≤50 years. Registry analyses demonstrated that while DDH was associated with an increased early risk of revision for dislocation, this excess risk diminished over time and accounted for a small proportion of long-term revisions. Importantly, because registries capture only revisions and not nonoperative dislocations, the true burden of instability is likely underestimated, particularly in the early postoperative period [8]. Institutional cohorts reported very low rates of instability-related revision, even in severe dysplasia, particularly when modern constructs, larger head sizes, or dual-mobility components were used. These findings suggest that although instability is a clinically relevant early complication, it is not the dominant long-term failure mechanism in young women undergoing THA for DDH.

Aseptic loosening. Aseptic loosening emerged as the most common cause of revision across studies with mid- to long-term follow-up. In DDH cohorts, acetabular loosening was particularly prominent, reflecting altered load transfer, bone stock deficiency, and technical challenges associated with dysplastic anatomy [80]. Several long-term institutional series demonstrated that loosening-related revisions often occurred beyond 10 years postoperatively, underscoring their relevance for lifetime implant durability in young patients. Registry studies comparing DDH and non-DDH diagnoses reported similar or modestly increased risks of aseptic loosening in DDH, although differences were often attenuated after adjustment for age and fixation method. These findings suggest that while dysplasia contributes to mechanical risk, implant fixation strategy and biomechanical restoration may be more important determinants of long-term fixation failure than diagnosis alone.

Infection, periprosthetic fracture, and other causes. Revision for infection and periprosthetic fracture occurred infrequently in women age ≤ 50 years across both DDH and non-DDH cohorts and did not demonstrate consistent diagnosis-specific differences. When reported, infection-related revisions were typically early and isolated events, while periprosthetic fractures occurred sporadically and were often associated with trauma or technical factors rather than underlying dysplasia [81]. Other causes of revision, including liner wear, osteolysis, implant breakage, or pain, were variably reported and often reflected implant era rather than patient diagnosis. These mechanisms collectively accounted for a smaller proportion of revisions but remain relevant contributors to lifetime revision risk given the long duration of implant use in young women [82].

### 6.4. Implant-Era Effects

Comparisons of revision and instability outcomes across studies are strongly influenced by implant-era effects. Earlier cohorts, often spanning the 1990s to early 2000s, predominantly employed smaller femoral head sizes, conventional polyethylene liners, and less modular implants, and they lacked access to contemporary stability-enhancing constructs such as dual-mobility cups. These legacy designs are associated with higher instability and wear-related failure rates, particularly in dysplastic hips [71].

In contrast, more recent cohorts reflect advances in bearing materials, fixation techniques, head size, and surgical planning, resulting in lower early instability and improved mid-term survivorship. As a result, apparent discrepancies between registry-era studies and modern institutional series likely reflect technological evolution rather than true differences in diagnosis-specific risk. Failure to account for implant era may therefore exaggerate or obscure the contemporary risk profile of DDH in young women [83].

### 6.5. Early Failure Mechanisms and Lifetime Revision Burden

Taken together, the evidence suggests that instability tends to occur early, when it occurs, and is increasingly preventable with contemporary reconstructive strategies. In contrast, aseptic loosening and mechanical failure appear to predominate in the long-term revision landscape, particularly in women with DDH who face decades of implant loading [84].

For young women undergoing THA, the primary lifetime burden is therefore less likely to arise from recurrent instability than from cumulative exposure to revision for fixation- or wear-related mechanisms. This distinction has important implications for surgical decision-making: strategies that minimize early instability must be balanced against those that optimize long-term fixation and bone preservation [82]. Ultimately, DDH should be viewed as a condition requiring not only strategies to ensure short-term stability but also durable biomechanical reconstruction to reduce lifetime revision risk. These distinct failure pathways and their relationship to lifetime revision burden are summarized in Table 6.

## 7. Modifiable Levers to Reduce Instability and Instability-Driven Revision

### 7.1. Surgical Planning Levers

Hip center/reconstruction strategy in DDH. Hip center position is a high-impact, modifiable surgical factor influencing stability in THA for DDH. The literature generally emphasizes placing the acetabular component at the anatomic hip center (low and medial) when feasible, given its role in restoring abductor lever arm, optimizing soft-tissue tension, and improving global hip biomechanics [85]. High hip center reconstruction is described as an alternative strategy in severe dysplasia, potentially reducing surgical complexity by avoiding excessive medialization or bone grafting and decreasing the need for aggressive femoral shortening, though at the cost of less favorable abductor mechanics and altered joint reaction forces [85,86,87]. A systematic review suggests that anatomic hip center placement may be associated with lower dislocation rates, whereas high hip center placement may be associated with fewer neurological complications [87]. Importantly, “acceptable” high hip center techniques are typically described as high but not lateral; lateralization is consistently linked to worse biomechanics and higher instability risk. Postoperative hip center position significantly affects gait biomechanics, with laterally placed centers correlating with increased internal rotation and proximally placed centers with greater extension [88]. Taken together, hip center planning is a primary preoperative planning consideration: when an anatomic center is safely achievable, prioritizing low/medial placement is generally aligned with stability goals; when not, a controlled high center strategy should minimize lateralization and anticipate soft-tissue consequences.

Femoral version and offset restoration considerations. Patients with DDH often require femoral stems that decouple metaphyseal fit from implanted version to accommodate abnormal femoral anatomy [86]. Abnormal femoral version is a recognized contributor to instability and highly variable in this population (with femoral anteversion frequently increased; female DDH patients average 30º anteversion compared with 22º in males) [89]. While preservation of native femoral anteversion has been associated with favorable outcomes, excessive increases in anteversion are associated with higher instability rates, lower functional scores, and knee dysfunction [90]. This makes “version control” a key intraoperative stability lever, particularly in DDH patterns where combined anteversion can unintentionally become excessive. Restoration of femoral offset represents a key modifiable surgical lever to mitigate instability, as biomechanical studies demonstrate that increased offset improves soft-tissue tension and jump distance, thereby increasing resistance to both anterior and posterior dislocation [91]. Offset restoration should be framed as a stability tool (tension/jump distance) rather than only a leg-length or gait objective; inadequate offset can “unmask” borderline component positioning by reducing functional jump distance and permitting earlier impingement. Where anatomy is severe, modular or version-adjustable stems can be strategically used to achieve a safer combined anteversion while maintaining (or increasing) offset.

### 7.2. Implant Construct Levers

Head size and liner geometry. Larger femoral heads increase dislocation resistance by approximately 22% compared to smaller heads [91]. Additionally, larger head diameters, particularly ≥40 mm, are associated with lower instability rates compared with smaller heads [72]. Mechanistically, larger heads increase jump distance and delay impingement, improving stability margins in positions that typically provoke dislocation. Liner geometry affects both prosthetic and bony impingement patterns. During standard ranges of motion, flexion produces both prosthetic and bony impingement, while extension results in prosthetic impingement when lipped liners are used [92,93]. Lipped liners also provide stability improvements, increasing posterior dislocation resistance [91]. However, lipped liners can trade posterior stability for reduced extension/external rotation clearance depending on lip orientation, so liner selection and lip position should be integrated with planned component version and anticipated functional positions.

Dual-mobility. Dual-mobility (DM) acetabular constructs were developed to simultaneously increase head-neck ratio, jump distance, and effective constraint, offering enhanced stability in both primary and revision THA, particularly for patients at elevated instability risk. Early and mid-term outcomes demonstrate lower dislocation rates with DM bearings compared to conventional single-articulation bearings in revision cohorts, suggesting potential utility in instability-prone settings [94]. Systematic reviews support reduced rates of dislocation and revision for DM constructs relative to standard bearings in mixed THA populations, although specific evidence in DDH patients remains sparse and long-term data are limited [95]. Thus, DM is best positioned as a risk-modifying construct for instability-prone anatomy or revision scenarios, while acknowledging that DDH-specific, long-horizon comparative data are still developing.

Bearing choice. Bearing surface selection can indirectly influence instability by enabling larger head sizes and alternative construct designs, such as dual-mobility articulations, which are associated with reduced dislocation risk while preserving functional range of motion [95]. Highly cross-linked polyethylene liners permit the use of larger diameters with reduced wear, improving stability while reducing the wear concerns associated with conventional polyethylene [96]. Wear characteristics and head size compatibility therefore remain central in construct-selection strategies aimed at minimizing instability in young, high-demand DDH patients [96]. In practical terms, bearing choice should be linked to a stability objective (safe head size, acceptable wear profile, and compatibility with DM when indicated), rather than treated as a purely wear-driven decision.

### 7.3. Clinician Tool

Table 7 provides a point-of-care instability prevention checklist designed for women aged ≤50 years undergoing THA, with prompts tailored to the unique anatomy and functional demands common in DDH. It organizes key decisions into a quick “risk snapshot” (e.g., dysplasia severity, prior surgery, hypermobility, spinopelvic issues, high-demand activities) and then guides the clinician through the major intraoperative levers that reduce instability—hip center strategy, a documented combined-version plan, restoration of offset/soft-tissue tension, and functional range-of-motion (ROM) impingement testing before closure. It also standardizes construct choices (head size, liner type, dual-mobility consideration), captures the surgical approach and capsular management plan, and closes with counseling tailored to young women on activity modification, red flags, and early follow-up. Each item includes a simple evidence rating (supported/limited/consensus) and a one-line notes field to make the checklist usable in real time and facilitate subsequent review.

## 8. Pregnancy and Childbirth After THA

### 8.1. Reported Outcomes

Pain and function. Among women who became pregnant after THA, pregnancy-associated hip symptoms are common but usually transient. Approximately 60% reported hip pain during pregnancy [12], with 21% having persistent pain postpartum and 4% describing severe pain [97]. Despite these symptoms, functional outcomes remained favorable: one study reported Harris Hip Scores improving from 94 points pre-pregnancy to 97 points at follow-up, along with significant gains in range of motion (217º pre-pregnancy to 241º post-pregnancy) [13]. Overall, available data suggest that many patients experience discomfort during pregnancy without a clear signal of meaningful long-term functional decline.

Instability and revision. Across available studies, pregnancy and mode of delivery were not associated with increased risk of dislocation, fracture, or loosening [97,98]. Similarly, no consistent association has been reported between childbirth and implant failure endpoints. Revision rates did not differ between women who become pregnant after THA and matched non-pregnant controls [13,99]. One study reported revision rates of 7.1% in the pregnancy group compared with 5.6% in non-pregnant controls, a difference that was not statistically significant [99], while another study found no association between childbirth and revision risk [12]. These findings are reassuring, but they are derived largely from small, retrospective cohorts with limited event counts, so they may be underpowered to detect rare complications.

Timing after THA. The mean interval from THA to pregnancy ranges from 2.5 to 3 years across small retrospective studies [13,100], with limited reporting on trimester-specific symptom onset or postpartum recovery. However, these studies did not analyze whether shorter or longer intervals correlate with differing complication rates. As a result, current evidence cannot define an “optimal” interval from THA to conception from an implant-safety standpoint, beyond general principles of recovery, rehabilitation, and stability.

Pregnancy and obstetric outcomes. Pregnancy-related outcomes were inconsistently reported. One large population-based study reported potentially concerning findings, including increased rates of stillbirth, preterm birth, small for gestational age, and emergency cesarean delivery among women with prior THA [15]. In contrast, smaller cohort studies found no increased pregnancy complications compared to the general population [100,101]. This divergence may reflect differences in study design, confounding (e.g., underlying disease severity and comorbidity), and outcome ascertainment, underscoring that obstetric risk signals are not yet settled. Additionally, a maternal history of DDH constitutes a positive family history, a recognized risk factor for DDH in offspring; female sex is also independently associated with increased DDH risk. These factors should be considered when counseling women with DDH regarding appropriate neonatal hip screening [3].

### 8.2. What Is Not Reported

Available studies suggest that pregnancy after THA is generally well tolerated [98,102], with 12–17% of women of childbearing age having at least one pregnancy post-THA [97]. However, substantial gaps remain in the literature, particularly with respect to DDH-specific outcomes and standardized reporting.

Although DDH is the most common indication for THA in young women, no published studies stratify pregnancy or childbirth outcomes by DDH severity, such as Crowe or Hartofilakidis classification. This is a major limitation because DDH-related factors (severity, prior osteotomy or other surgery, and challenges in version and offset restoration) plausibly modify symptom burden and stability risk during pregnancy and postpartum. Existing DDH-focused literature primarily addresses long-term THA outcomes and does not address pregnancy-specific outcomes [55,103,104,105,106]. In addition, studies lack uniform definitions for complications, use varying follow-up periods, and employ different outcome measures, making meta-analysis challenging. Standardized endpoints (e.g., patient-reported pain trajectories by trimester, functional PROMs, imaging/loosening criteria, and instability events with clear definitions) are needed to allow meaningful synthesis across cohorts.

Long-term postpartum outcomes beyond the immediate perioperative periods are poorly characterized. In particular, the potential impact of breastfeeding, postpartum weight retention, and subsequent pregnancies on implant longevity remains unexplored. Additional unreported domains include postpartum activity demands (infant carrying/lifting), return-to-sport and return-to-work timelines, pelvic floor/biomechanical changes, and whether postpartum rehabilitation strategies influence symptoms or instability risk.

### 8.3. Translation to Practice

Table 8 provides a practical, stage-based counseling framework for women aged ≤50 years with a prior THA who are planning pregnancy or childbirth (including a DDH-specific add-on). It structures conversations and documentation across four stages (preconception, pregnancy, labor/delivery planning, and the first 6–12 months postpartum), so clinicians consistently address baseline stability, implant/instability history, timing and conditioning, symptom monitoring and movement guidance, fall/load management, clear escalation triggers, and coordinated obstetric and anesthesia positioning precautions. It also prompts individualized counseling on mode of delivery and postpartum caregiving mechanics (lifting/carrying/feeding positions) with a graded return-to-activity plan. Each item is paired with a simple evidence rating (supported/limited/consensus) and a one-line notes field to keep counseling actionable, repeatable, and reviewable, while explicitly flagging that DDH anatomy may narrow the stability margin even when overall outcomes are generally reassuring.

### 8.4. Key Research Needs Specific to Pregnancy After THA in DDH Versus Non-DDH

Future research should focus on DDH-specific pregnancy outcomes after THA using prospective, multicenter cohort designs with standardized, pregnancy-relevant endpoints and direct comparison groups (DDH vs. non–DDH etiologies). Studies should pre-specify DDH phenotype and severity using validated classification systems (e.g., Crowe or Hartofilakidis) and test whether risks differ by grade, because the current literature does not clarify whether pregnancy-related pain trajectories, instability events, obstetric complications, or revision risk meaningfully vary across dysplasia severity or relative to non-dysplastic indications. Equally important is detailed contemporary surgical information, including hip center strategy (anatomic vs. high), combined anteversion targets, offset restoration, and prior dysplasia-related procedures, so analyses can link pregnancy tolerance to the reconstructive choices that may influence the stability margin.

Evidence-based guidance on the optimal interval between THA and conception remains lacking: existing studies primarily report descriptive timing without correlating interval length to instability, revision, or symptom burden. This gap is particularly relevant in DDH, where altered hip biomechanics, higher baseline instability risk [13,100], and more frequent need for complex reconstruction may influence physiologic tolerance to pregnancy-related weight gain, ligamentous laxity, and postural change. Future cohorts should therefore treat time to conception as an exposure variable (not just a descriptor) and evaluate associations across clinically relevant intervals (e.g., <1 year, 1–2 years, >2 years) while adjusting for age, activity demands, and implant factors.

Methodologically, future studies should adopt uniform definitions and core outcome sets tailored to pregnancy: trimester-specific pain and function, standardized PROMs, objective documentation of instability (subluxation sensation vs. true dislocation), imaging-based loosening criteria where appropriate, and detailed delivery data, including mode of delivery, anesthesia type, and hip positioning/ROM precautions, to determine whether specific intrapartum positions or maneuvers are associated with hip symptoms or instability. Longer-term follow-up should include implant survivorship and reoperation stratified by pregnancy history, number of pregnancies, and time since last pregnancy, rather than treating pregnancy as a binary exposure.

The postpartum period also needs focused study, as it likely concentrates many relevant stressors (rapid activity escalation, repetitive lifting/carrying, sleep deprivation, deconditioning). Research should specifically test the cumulative effects of breastfeeding, postpartum weight retention, and multiple pregnancies on symptoms, function, and implant longevity, particularly in dysplastic hips where stability margins may be narrower. Finally, studies should evaluate whether stability-enhancing constructs (e.g., dual mobility, larger head sizes, liner geometry, and version-adjustable stems) modify pregnancy-related risks in DDH, especially when anatomic constraints limit ideal component positioning, thereby moving the field from reassurance based on sparse retrospective data to actionable, risk-stratified counseling and surgical decision-making.

## 9. Discussion

### 9.1. Principal Findings

This narrative review synthesizes contemporary evidence on prosthesis longevity in women aged 50 years or younger undergoing primary total hip arthroplasty, with a deliberate comparison of between developmental dysplasia of the hip and non-dysplastic diagnoses through a mechanism-based lens. Three consistent themes emerged.

First, in young women, long-term outcomes are determined less by early implant survivorship and more by which failure mechanism occurs first and whether that first failure initiates a sequence of repeat procedures. In this population, early complications can have disproportionate lifetime consequences because any reoperation increases cumulative revision burden across decades of implant exposure [107].

Second, DDH should not be viewed simply as osteoarthritis occurring at a younger age. Rather, it is a distinct structural and biomechanical phenotype characterized by acetabular deficiency, altered femoral morphology and version, and soft-tissue adaptations that influence reconstruction and stability. As a result, comparisons between dysplasia and non-dysplastic diagnoses are most clinically meaningful when phenotype, severity, and reconstructive strategy are considered, rather than relying on diagnosis labels alone [68].

Third, the evidence base on pregnancy and childbirth after total hip arthroplasty is generally reassuring for implant-related endpoints such as dislocation and revision, but it remains limited and heterogeneous, with limited diagnosis-specific and severity-stratified reporting. Some population-level studies suggest higher rates of selected obstetric complications among women with prior total hip arthroplasty, while smaller series report outcomes comparable to general obstetric population. These mixed findings underscore the need for multidisciplinary counseling that is transparent about uncertainty and grounded in what is known versus what remains speculative.

### 9.2. Clinical Implications

What to tell women aged ≤50 years with DDH about instability and revision risk. Counseling for young women with dysplasia should be framed around two ideas. Overall modern total hip arthroplasty outcomes are generally favorable, but the margin for instability may be narrower in dysplasia, and instability can lead to repeat procedures. For patients with decades of expected implant use, an early instability event can be more consequential than a transient complication, because it can prompt additional operations that shape lifetime revision burden [108]. A practical counseling message is that an important early surgical priority is preventing instability early, because early instability can trigger subsequent procedures. This framing also helps align patient expectations with the clinical reality that survivorship alone does not capture the lived impact of recurrent instability, repeat imaging, restrictions, anxiety about movement, and time away from work or caregiving. Counseling should explicitly acknowledge uncertainty. Dysplasia severity classifications are inconsistently reported across studies and are often not linkable in registries, which limits precise, phenotype-specific prognostication. Therefore, counseling should be risk stratified when phenotype and severity are known and explicitly framed as general guidance when they are not.

How modifiable choices can reduce instability-driven revision. The most actionable implication of this review is that instability prevention can be translated into a structured plan that links preoperative assessment, intraoperative execution, and postoperative counseling. Several modifiable levers recur across the literature and align with dysplasia-specific reconstructive challenges. Reconstruction strategy and biomechanics are important. Hip center planning and restoration of offset influence abductor mechanics, soft-tissue tension, and impingement tolerance, all of which contribute to functional stability during daily activities. These choices are particularly important in dysplasia because acetabular deficiency and femoral morphology can constrain component placement and tensioning [109]. Version control is central. Dysplasia-related femoral anatomy can predispose to suboptimal combined version if the version is not deliberately planned and controlled. A documented combined-version plan, paired with intraoperative assessment of functional stability through a ROM testing, provides a practical safeguard against unrecognized instability risk [110]. Construct choices function as stability tools. Head size strategy, liner geometry, and selective use of dual-mobility constructs can increase the stability margin by increasing jump distance and reducing impingement-related dislocation risk. These decisions should be framed as risk modifiers rather than preferences, while acknowledging that observational comparisons are influenced by confounding because higher-risk hips are more likely to receive stability-enhancing constructs. Together, these implications support a continuity-of-care model from preoperative planning through intraoperative execution in which phenotype and severity are documented when available; a stability plan that includes hip center, combined version, and offset is defined; constructs that preserve stability margin are chosen; and stability through functional testing is verified before closure.

How to counsel about pregnancy and childbirth with limited evidence. Patient-centered counseling should balance reassurance, symptom preparedness, and coordinated delivery planning. Available studies suggest that pregnancy after total hip arthroplasty is feasible, and most reports do not show a consistent increase in dislocation or revision associated with pregnancy or delivery mode [97]. However, hip discomfort during pregnancy appears common and may persist in a minority of patients postpartum, which should be anticipated and proactively addressed. Because obstetric outcomes have been inconsistently reported and signals differ across study designs, counseling should emphasize coordination with obstetrics and anesthesia. The practical focus should be on communicating ROM limits, avoiding forced extremes during labor and delivery positioning, and establishing clear escalation triggers for new mechanical symptoms [15]. For patients with developmental dysplasia of the hip, counseling can include the additional consideration that individual anatomy and reconstructive choices may reduce tolerance for extreme positions, even if overall implant safety data are reassuring. This point should be labeled as consensus-based guidance rather than a quantified risk estimate, given the scarcity of dysplasia severity-stratified pregnancy outcomes.

### 9.3. Strengths of This Narrative Review

This review is women-centered and mechanism-oriented, organizing heterogeneous evidence around clinically meaningful pathways that directly affect long-term outcomes, particularly instability and revision. It treats developmental dysplasia of the hip as a reconstructive phenotype rather than a binary label, which better reflects surgical decision-making and patient counseling needs. It also translates evidence into actionable frameworks designed to standardize how clinicians assess risk, document modifiable decisions, and counsel young women in a repeatable, reviewable way [111].

### 9.4. Limitations

Several limitations temper inference. Substantial heterogeneity exists across studies in age thresholds, outcome definitions, follow-up horizons, and reporting granularity, which limits direct comparisons and precludes reliable quantitative pooling. Developmental dysplasia of the hip definitions and severity reporting are inconsistent, and many registries do not capture radiographic severity, restricting phenotype-specific counseling. Observational studies are also vulnerable to confounding by indication, particularly when stability-enhancing constructs are preferentially used in higher-risk hips [112,113].

Implant-era effects further complicate interpretation because older series include legacy bearings, smaller head sizes, and different fixation strategies that may not generalize to current practice [114,115,116,117,118]. Finally, the pregnancy literature is limited by small sample sizes, inconsistent endpoint selection, and minimal dysplasia stratification, which constrains guidance on optimal timing of conception, severity modifiers, and postpartum exposures.

Despite these limitations, the convergent clinical message remains clear. In women 50 years or younger, particularly those with developmental dysplasia of the hip, a key modifiable early prevention target is minimizing instability through deliberate reconstructive planning and stability-focused construct choices, while long-term implant longevity also depends on durable fixation, biomechanical reconstruction, and mitigation of wear-related failure [112,113,119,120].

## 10. Future Research Agenda

### 10.1. Highest-Priority Studies

The highest priority for future research is the development of large, diagnosis-aware datasets and prospective studies that directly examine the mechanisms most relevant to young women undergoing total hip arthroplasty. First, linkage of arthroplasty registries with obstetric and maternal health databases is needed to evaluate pregnancy incidence, delivery outcomes, implant survivorship, and revision risk at the population level, with explicit stratification by developmental dysplasia of the hip and non-dysplastic diagnoses.

Second, multicenter registry or cohort studies should move beyond binary diagnostic labels and examine how dysplasia severity interacts with construct strategy and surgical decisions to influence instability and instability-driven revision, thereby enabling assessment of effect modification rather than isolated predictors [18,74,75,81]. Third, prospective cohorts with predefined and uniform instability endpoints, patient-reported outcomes, and structured postpartum follow-up are essential to reduce heterogeneity, clarify symptom trajectories, and determine whether pregnancy timing, number of pregnancies, or reconstructive variables are associated with long-term implant performance.

### 10.2. Standardization Recommendations

Progress in this field depends on consistent data capture and transparent reporting, making the adoption of a minimum standardized dataset essential [20,32]. Future studies should uniformly record patient demographics and comorbidities, primary diagnosis with validated dysplasia severity classifications when applicable, prior hip procedures, and detailed surgical and implant variables, including approach, hip center strategy, version targets or measurements, offset restoration, fixation type, head size, liner geometry, and use of dual-mobility or constrained constructs [30,119,120,121,122]. Outcome reporting should employ clear, predefined definitions of dislocation, recurrent instability, revision, and reoperation, with standardized follow-up intervals and explicit distinction between hip-based and patient-based analyses [112,113]. Patient-reported pain and function measures, return-to-activity timelines, and pregnancy-specific domains such as timing of conception, delivery mode, postpartum activity demands, and instability or revision events temporally related to pregnancy should also be incorporated. Consistent use of these core elements improve cross-study comparability, enable more reliable meta-analysis, and support more precise counseling and prevention strategies for young women undergoing total hip arthroplasty [59,123,124].

## 11. Conclusions

In women undergoing THA at age 50 years or younger, prosthesis longevity is determined less by early implant survivorship than by the mechanisms that precipitate reoperation and revision over decades of implant exposure. Developmental dysplasia of the hip is a distinct structural and biomechanical phenotype whose anatomy and reconstructive demands shape instability risk and revision pathways differently from non-dysplastic hip disease. Altered femoral version, acetabular deficiency, and challenges in restoring offset and soft-tissue tension can narrow the stability envelope and influence revision pathways. Accordingly, comparisons between DDH and non-DDH populations are most meaningful when interpreted through the lens of phenotype severity and reconstructive strategy rather than diagnosis alone.

The major early clinical priority in young women undergoing THA is the prevention of instability and the revision cascade it can initiate. Durable outcomes depend on deliberate, modifiable surgical decisions, including characterization of dysplasia severity, careful planning of combined version, restoration of offset and soft-tissue tension, intraoperative assessment of functional impingement and stability, and selective use of stability-enhancing constructs when anatomy or patient-specific risk factors warrant, as well as durable fixation and bone preservation. Contemporary evidence suggests that instability risk in DDH is highly modifiable and that outcomes may approach those of non-dysplastic hips when modern reconstructive principles are applied.

Pregnancy after THA appears feasible and generally reassuring regarding dislocation and revision risk, although the available evidence remains limited, heterogeneous, and largely devoid of DDH-specific or severity-stratified analyses. A high-priority next step is the development of standardized, phenotype-aware reporting frameworks and linked arthroplasty-obstetric datasets that capture instability events, cause-specific revision, reproductive outcomes, and DDH severity. Such efforts will enable more precise risk stratification, individualized counseling, and targeted prevention strategies for young women undergoing THA across the full spectrum of dysplastic and non-dysplastic hip disorders.

## Figures and Tables

**Table 1:** Comparative studies (DDH versus OA/other).

Study	Country/Data	Design	Sample (Key)	Age (y)	DDH Definition	Severity	Comparator	FU
Thillemann 2008 † [17]	Denmark; Reg	Reg cohort	Dysplasia n = 890 hips	NR	RegDx	NR	OA	4.6 y
Siddiqi 2020 † [18]	USA; Reg	PM cohort	DDH 603 (73% F)	51	RegDx	NR	OA	30 d
Rajan 2025 [19]	USA; Reg	PM cohort	DDH 18–50: 2802	45	RegDx	NR	OA	2 y
Mortazavi 2022 [20]	Iran; SC	Ret cohort	DDH 127/143 F	42	Rad	Crowe I–IV	OA/other	40 mo
Engesaeter 2012 † [21]	Nordic; Reg	Reg cohort	DDH 12,068	55	RegDx	NR	OA	6.4 y
Boyle 2012 † [22]	NZ; Reg	Reg cohort	DDH 1205	49	RegDx	NR	OA	~10 y
Bus 2024 † [23]	Netherlands; Reg	Reg cohort	DDH 3032 (<55)	44	RegDx	NR	OA/Perthes	10 y
Clark 2025 † [24]	USA; Reg	PM cohort	DDH 38 hips	52	Rad	Crowe I–IV	OA	5.2 y
Ashraf 2014 † [25]	USA; Reg	Matched cohort	DDH 354 hips	49	Rad	Crowe 0–IV	OA	≥2 y
Chen 2018 † [26]	China; SC	Pros cohort	39/45 F	~50	Rad	Crowe II–III	Healthy	~5 y
Kawasaki 2017 † [27]	Japan; SC	Pros imaging	mostly F	~63	Rad	Crowe I–III	ONFH	12 mo
Galea 2018 † [28]	USA/Japan; SC	Pros cohort	114/123 hips F	55	Rad	Crowe I–IV	Nondysplastic	13.8 y
Gharanizadeh 2025 † [29]	Iran; MC	Ret cohort	83% F	43	Rad	Crowe III–IV	Other dx	90 d

Abbreviations: Reg = registry/database; SC = single-center; MC = multicenter; PM = propensity matched; Rad = radiographic; RegDx = registry or administrative diagnosis; OA = osteoarthritis; ONFH = osteonecrosis of the femoral head; NR = not reported; FU = follow-up. † Not restricted to women aged ≤50 years; relevant subgroups extracted where available.

**Table 2:** DDH-only and within-DDH comparisons.

Study	Country/Data	Design	Sample (Key)	Age (y)	DDH Definition	Severity	Within-DDH Comparison	FU
Zhang 2024 [30]	China; SC	PM cohort	DDH only	~47	Rad	I–IV	Robotic vs. manual	≥6 mo
Wang 2012 [31]	China/USA; Reg	Ret cohort	611/820 F	50	Rad	I–IV	Head/version factors	~3 y
Vigdorchik 2020 [32]	USA; MC	Ret CS	56/79 F	45	Rad	I–IV	CT templating	3.1 y
Zeng 2017 [33]	China; SC	Ret CS	37/45 F	41	Rad	IV	Cohort	9.8 y
Wang 2017 [34]	China; SC	Ret cohort	49/62 F	47	Rad	IV	Cohort	10 y
Tu 2022 [35]	China; SC	Ret CS	8/12 F	33	Rad	IV	Cohort	72 mo
Ors 2022 [36]	Turkey; SC	Ret CS	81/91 F	43	Rad	IV	Cohort	8.4 y
Mu 2016 [37]	China; SC	Ret CS	58 pts	36	Rad	IV	Cohort	70 mo
Viamont-Guerra 2020 [38]	France; SC	Ret CS	22/29 hips F	49	Rad	III–IV	Cohort	8.4 y
Ukaj 2025 [39]	Kosovo; MC	Pros CS	19/22 F	49	Rad	IV	Cohort	≥6 mo
Liu 2021 [40]	China; SC	Ret cohort	82% F	~45	Rad	III–IV	DAA vs. PLA	~3 y
Shen 2020 [41]	China; SC	Ret CS	54/69 F	~46	Rad	II–III	Hip center height	8.9 y
Fukushi 2018 † [42]	Japan; SC	Ret cohort	86/100 F	66	Rad	I–III	Hip center height	12 mo
Kim 2023 † [43]	Korea; SC	Ret cohort	126/172 F	54	Rad	I–IV	DM vs. fixed	≥2 y
Sato 2022 † [44]	Japan; SC	PM cohort	84 vs. 84	66	Rad	I–II	Robotic vs. manual	3 mo
Yacovelli 2021 † [45]	USA; SC	PM cohort	DDH only	~40	Rad	I–II	Prior PO	3.8 y
Osawa 2016 † [46]	Japan; SC	Matched	46/48 F	57	Rad	I–II	Post-PAO vs. primary	5.4 y
Osawa 2019 † [47]	Japan; SC	Matched	49/55 F	57	Rad	I–III	CT simulation	—
Minoda 2006 † [48]	Japan; SC	Matched	all F	~56	Rad	II–III	Chiari vs. none	3.0 y
Ikezaki 2024 † [49]	Japan; SC	PM cohort	~87% F	~57	Rad	I	Shelf vs. none	~7–9 y
Benad 2022 † [50]	France; SC	Case–control	61 vs. 63	~48	Rad	NR	Shelf vs. none	13 y
Asai 2025 † [51]	Japan; SC	PM cohort	PAO 50 hips	56	Rad	I–III	Post-PAO vs. primary	13 y
Holzapfel 2012 † [52]	Germany; MC	Matched	~75% F	~53	Rad	Harto II	Socket vs. graft	~6 y
Matsushita 2022 † [53]	Japan; SC	Ret CS	179/201 F	62	Rad	I–IV	Cohort	11.4 y
Kristoffersson 2021 † [54]	Sweden; SC	Ret CS	27/50 F	NR	Rad	I–IV	Templating	Immediate
Fahlbusch 2023 † [55]	Germany; SC	Ret cohort	91/110 F	51	Clin + Rad	I–IV	Cohort	23 y
Di Martino 2021 † [56]	Italy; Reg	Reg cohort	DDH 5761	~60	RegDx	NR	Stem strata	to 17 y
Castagnini 2022 † [57]	Italy; Reg	Reg cohort	DDH 6429	~60	RegDx	NR	Stem strata	to 19 y
DeNovio 2025 † [58]	USA; Reg	Ret cohort	24 pts	48	Rad	IV	Cohort	18 y
Colo 2016 † [59]	Netherlands; SC	Ret CS	18 pts	48	Rad	I–IV	Cohort	20 y
Caylak 2021 † [60]	Turkey; SC	Ret CS	43/50 F	42	Rad	IV	Cohort	~11 y
Biant 2009 † [61]	Australia; SC	Pros CS	17/22 F	45	Rad	III–IV	Cohort	~10 y
Berninger 2019 † [62]	Germany; SC	Ret cohort	39/50 F	59	Rad	II–IV	Cohort	38 mo

Abbreviations: SC = single-center; MC = multicenter; Reg = registry/database; PM = propensity matched; CS = case series; FU = follow-up; OA = osteoarthritis; PAO = periacetabular osteotomy; PO = pelvic osteotomy; DDH definition: Rad = radiographic; RegDx = registry/ICD; Clin + Rad = clinical + radiographic; NR = not reported. Outcome units were classified as hip-based (per implant) or patient-based (per individual) according to the original study reporting. When the unit was not explicitly stated, implant-level outcomes (e.g., revision, dislocation, survivorship) were interpreted as hip-based, whereas complication and patient-reported outcomes were considered patient-based. † Not restricted to women ≤50 years; relevant subgroups extracted where available.

**Table 3:** Comparative instability and revision outcomes.

Study	Population	Dislocation	Revision	Key Finding
Thillemann 2008 [17]	Registry DDH vs. OA	↑ early in DDH	NR	Higher early instability signal
Siddiqi 2020 [18]	NSQIP DDH vs. OA	NR	Similar	No early difference (30 d)
Rajan 2025 [19]	DDH (≤50 years) vs. OA	Slight ↑	Slight ↑	Modest risk elevation
Mortazavi 2022 [20]	DDH vs. OA/other	↑ in DDH	↑	High-grade DDH drives risk
Engesaeter 2012 [21]	Nordic registry	↑ in DDH	↑	Consistent registry signal
Boyle 2012 [22]	NZ registry	Slight ↑	↑	Younger DDH higher revision
Bus 2024 [23]	LROI < 55	Mixed	↑	Age modifies risk
Clark 2025 [24]	Matched DDH vs. OA	Similar	Similar	Contemporary parity
Galea 2018 [28]	DDH vs. nondysplastic	↑ with severity	↑	Severity-dependent risk

DDH = developmental dysplasia of the hip; OA = osteoarthritis; ONFH = osteonecrosis of the femoral head; NR = not reported; arrows indicate the relative direction of risk within each study and do not represent pooled effect estimates.

**Table 4:** Within-DDH instability modifiers.

Domain	Factor	Direction	Evidence	Key Source(s)
Anatomy	High Crowe grade	↑ risk	Moderate	Mortazavi 2022 [20]; Galea 2018 [28]
Implant	Head < 32 mm	↑ risk	Strong	Wang 2012 [31]
Implant	Dual mobility	↓ risk	Moderate	Kim 2023 [43]
Implant	Larger head	↓ risk	Moderate	Hailer 2012 [10]; Howie 2012 [71]; Pitz-Gonçalves 2023 [72]
Technique	Malcombined version	↑ risk	Strong	Wang 2012 [31]
Technique	Inadequate offset	↑ risk	Moderate	Mortazavi 2022 [20]
Technique	High hip center (excessive)	↑ risk	Mixed	Fukushi 2018 [42]; Shen 2020 [41]
Technique	Accurate cup positioning	↓ risk	Moderate	Zhang 2024 [30]
Strategy	Robotic assistance	↓ outliers	Emerging	Zhang 2024 [30]; Sato 2022 [44]
Strategy	Direct anterior approach	Mixed	Limited	Liu 2021 [40]
Prior surgery	Prior pelvic osteotomy	Mixed	Limited	Yacovelli 2021 [45]; Osawa 2016 [46]; Osawa 2019 [47]

DDH = developmental dysplasia of the hip. Evidence grading: Strong = consistent signal across multiple studies or adjusted analyses; Moderate = supported by ≥1 comparative study; Limited = single or small cohort signal; Emerging = early or technology-driven signal; Interpretation: direction reflects the reported association with instability risk within DDH cohorts. ↑ = increased; ↓ = decreased.

**Table 5:** Severe DDH (Crowe III–IV) cohorts.

Study	Severity	N	Dislocation	Revision	Takeaway
Zeng 2017 [33]	Crowe IV	45	Low	Low	Modern techniques effective
Wang 2017 [34]	Crowe IV	62	Acceptable	Acceptable	Complex but durable
Mu 2016 [37]	Crowe IV	71 hips	Low	Low	Subtrochanteric strategy works
Ors 2022 [36]	Crowe IV	91	Low	Low	Experienced centers
Ukaj 2025 [39]	Crowe IV	22	Low	NR	Short-term safe
Caylak 2021 [60]	Crowe IV	50	Low	Low	Long-term acceptable
DeNovio 2025 [58]	Crowe IV	28 hips	Low	Low	Survivorship maintained
Biant 2009 [61]	Crowe III–IV	22	Low	Low	Specialized reconstruction

DDH = developmental dysplasia of the hip; NR = not reported.

**Table 6:** Conceptual pathways of early instability, instability-related revision, and long-term implant failure after total hip arthroplasty (THA) in young women with developmental dysplasia of the hip (DDH).

Pathway	Contributing Factors/Mechanisms	Clinical Event	Potential Consequence
Early instability	DDH anatomy; component position and combined version; femoral offset and soft-tissue tension; femoral head size; implant construct	Dislocation/instability	May resolve without revision or progress to recurrent instability
Instability-related revision	Recurrent or severe instability	Revision for instability	Component exchange or revision → increased lifetime revision burden
Long-term implant failure	Long duration of implant use; wear; osteolysis; mechanical failure; aseptic loosening	Late prosthesis failure	All-cause revision

**Table 7:** Instability Prevention Checklist for Women Aged ≤50 Years Undergoing THA (DDH versus Non-DDH).

Domain	Item No.	Checklist Item	Response/Selection	Evidence Grading *	Notes
Patient risk snapshot	—	DDH severity: ☐ Crowe ☐ Hartofilakidis ☐ NR	☐ Yes ☐ No	—	______
Patient risk snapshot	—	Prior hip surgery	☐ Yes ☐ No	—	______
Patient risk snapshot	—	Marked extremes of ROM/hypermobility	☐ Yes ☐ No	—	______
Patient risk snapshot	—	High-demand function (caregiving/sport/work)	☐ Yes ☐ No	—	______
Patient risk snapshot	—	Spinopelvic stiffness/imbalance suspected or known	☐ Yes ☐ No	—	______
Patient risk snapshot	—	Other risk factor	☐ Yes ☐ No	—	______
Intraoperative levers	1	Reconstruction strategy (DDH): anatomic versus high hip center versus augmentation/graft	☐ Anatomic ☐ High ☐ Graft	___	______
Intraoperative levers	2	Version plan (combined anteversion) documented and assessed intraoperatively	☐ Yes ☐ No	___	______
Intraoperative levers	3	Offset and soft-tissue tension restored (target and verification)	☐ Yes ☐ No	___	______
Intraoperative levers	4	Impingement and stability tested through functional ROM before closure	☐ Yes ☐ No	___	______
Construct choices	5	Head size strategy	☐ ≤28 mm ☐ 32 mm ☐ 36 mm ☐ ≥40 mm ☐ Limited	___	______
Construct choices	6	Liner strategy	☐ Standard ☐ Elevated rim ☐ Other	___	______
Construct choices	7	Dual-mobility consideration for high-risk anatomy/history	☐ Used ☐ Considered ☐ Not indicated	___	______
Approach/soft tissue	8	Capsular/soft-tissue strategy documented (repair/closure plan)	☐ Yes ☐ No	___	______
Immediate counseling	9	Activity plan tailored to risk (caregiving/lifting/sport/work)	☐ Yes ☐ No	___	______
Immediate counseling	10	Red flags explained and early follow-up plan established	☐ Yes ☐ No	___	______

Abbreviations: DDH = developmental dysplasia of the hip; THA = total hip arthroplasty; ROM = range of motion; NR = not reported. * Evidence Grading: S = supported by published evidence; L = limited evidence; C = consensus-based recommendation.

**Table 8:** Pregnancy and Childbirth After THA in Women Aged ≤50 Years (DDH versus Non-DDH): Counseling Checklist.

Domain	Item No.	Counseling Item	Response	Evidence Grading *	Notes
Preconception	1	Confirm hip stability baseline (no episodes of giving way, sensation of subluxation, or recurrent pain)	☐ Yes ☐ No	___	______
Preconception	2	Review implant factors relevant to stability (surgical approach and construct; history of instability)	☐ Yes ☐ No	___	______
Preconception	3	Timing discussion (interval after THA before pregnancy; shared plan documented)	☐ Yes ☐ No	___	______
Preconception	4	Activity/conditioning plan (core/hip strengthening, fall prevention, lifting mechanics)	☐ Yes ☐ No	___	______
During pregnancy	5	Symptom monitoring plan (new mechanical pain, sensation of instability, functional decline)	☐ Yes ☐ No	___	______
During pregnancy	6	Movement guidance (avoid provocative extreme hip flexion and rotation)	☐ Yes ☐ No	___	______
During pregnancy	7	Falls and load management (fatigue, balance, footwear, assistive device if needed)	☐ Yes ☐ No	___	______
During pregnancy	8	Escalation triggers reviewed (clunking/subluxation, true dislocation, inability to bear weight)	☐ Yes ☐ No	___	______
Labor and delivery	9	Multidisciplinary delivery plan documented (obstetrics and anesthesia teams aware of THA)	☐ Yes ☐ No	___	______
Labor and delivery	10	Positioning precautions discussed (avoid forced extremes; communicate ROM limits)	☐ Yes ☐ No	___	______
Labor and delivery	11	Mode-of-delivery counseling individualized (vaginal versus cesarean delivery; rationale documented)	☐ Yes ☐ No	___	______
Postpartum	12	Early postpartum hip check-in (pain, function, instability symptoms)	☐ Yes ☐ No	___	______
Postpartum	13	Safe caregiving mechanics (lifting, carrying, floor-to-stand transitions, feeding positions)	☐ Yes ☐ No	___	______
Postpartum	14	Return-to-activity progression (graded plan; avoid abrupt high-flexion/rotation loads)	☐ Yes ☐ No	___	______
DDH-specific	15	Document DDH phenotype/severity and counsel that anatomy may narrow the stability margin	☐ Yes ☐ No	___	______

Abbreviations: DDH = developmental dysplasia of the hip; THA = total hip arthroplasty; ROM = range of motion. * Evidence Grading: S = supported by published evidence; L = limited evidence; C = consensus-based recommendation.

## Data Availability

No new data were created or analyzed in this study. Data sharing is not applicable to this article.

## Supplementary Materials

The following supporting information can be downloaded at: https://www.mdpi.com/article/10.3390/jcm15187220/s1, Table S1. Characteristics of included studies (women aged ≤50 undergoing THA for DDH versus non-DDH); Table S2. Instability/dislocation outcomes in women aged ≤50 years: DDH versus non-DDH (primary endpoint); Table S3. Revision outcomes and revision mechanisms in women aged ≤50 years undergoing THA: DDH versus non-DDH. The studies summarized in Supplementary Tables S1–S3 are cited in the main reference list [17,18,19,20,21,22,23,24,25,26,27,28,29,30,31,32,33,34,35,36,37,38,39,40,41,42,43,44,45,46,47,48,49,50,51,52,53,54,55,56,57,58,59,60,61,62,123,125,126,127].

## References

[1] Shan L., Shan B., Graham D., Saxena A. (2014). Total hip replacement: A systematic review and meta-analysis on mid-term quality of life. Osteoarthr. Cartil..

[2] Engesæter I.Ø., Lehmann T., Laborie L.B., Lie S.A., Rosendahl K., Engesæter L.B. (2011). Total hip replacement in young adults with hip dysplasia: Age at diagnosis, previous treatment, quality of life, and validation of diagnoses reported to the Norwegian Arthroplasty Register between 1987 and 2007. Acta Orthop..

[3] Loder R.T., Skopelja E.N. (2011). The epidemiology and demographics of hip dysplasia. ISRN Orthop..

[4] Pun S. (2016). Hip dysplasia in the young adult caused by residual childhood and adolescent-onset dysplasia. Curr. Rev. Musculoskelet. Med..

[5] Yang S., Cui Q. (2012). Total hip arthroplasty in developmental dysplasia of the hip: Review of anatomy, techniques and outcomes. World J. Orthop..

[6] Bicanic G., Barbaric K., Bohacek I., Aljinovic A., Delimar D. (2014). Current concept in dysplastic hip arthroplasty: Techniques for acetabular and femoral reconstruction. World J. Orthop..

[7] Muddaluru V., Boughton O., Donnelly T., O’Byrne J., Cashman J., Green C. (2023). Developmental dysplasia of the hip is common in patients undergoing total hip arthroplasty under 50 years of age. SICOT-J.

[8] Salman L.A., Alzobi O.Z., Al-Ani A., Hantouly A.T., Al-Juboori M., Ahmed G. (2024). The outcomes of total hip arthroplasty in developmental dysplasia of the hip versus osteoarthritis: A systematic review and meta-analysis. Eur. J. Orthop. Surg. Traumatol..

[9] Kunutsor S.K., Barrett M.C., Beswick A.D., Blom A.W., Whitehouse M.R., Wylde V. (2019). Risk factors for dislocation after primary total hip replacement: A systematic review and meta-analysis of 125 studies involving approximately five million hip replacements. Lancet Rheumatol..

[10] Hailer N.P., Weiss R.J., Stark A., Kärrholm J. (2012). The risk of revision due to dislocation after total hip arthroplasty depends on surgical approach, femoral head size, sex, and primary diagnosis. Acta Orthop..

[11] Hoskins W., Bingham R., Hatton A., de Steiger R.N. (2020). Standard, large-head, dual-mobility, or constrained-liner revision total hip arthroplasty for a diagnosis of dislocation: An analysis of 1275 revision total hip replacements. J. Bone Jt. Surg. Am. Vol..

[12] Sierra R.J., Trousdale R.T., Cabanela M.E. (2005). Pregnancy and childbirth after total hip arthroplasty. J. Bone Jt. Surg. Br. Vol..

[13] McDowell C.M., Lachiewicz P.F. (2001). Pregnancy after total hip arthroplasty. J. Bone Jt. Surg. Am. Vol..

[14] Galanis A., Dimopoulou S., Karampinas P., Koutalos A., Papakostidis C., Malizos K. (2024). Assessing the effects and challenges of total hip arthroplasty before pregnancy and childbirth: A systematic review. J. Funct. Morphol. Kinesiol..

[15] Kuitunen I., Artama M., Eskelinen A., Skyttä E.T., Huhtala H., Uotila J. (2019). Pregnancy outcome in women after total hip replacement: A population-based study. Eur. J. Obstet. Gynecol. Reprod. Biol..

[16] Sukhera J. (2022). Narrative reviews: Flexible, rigorous, and practical. J. Grad. Med. Educ..

[17] Thillemann T.M., Pedersen A.B., Johnsen S.P., Søballe K. (2008). Inferior survival of total hip arthroplasty in patients with hip dysplasia compared with primary osteoarthritis: A nationwide Danish study. Acta Orthop..

[18] Siddiqi A., White P.B., Sloan M., Fox D., Piuzzi N.S., Sankar W.N., Sheth N.P. (2020). Total Hip Arthroplasty for Developmental Dysplasia of Hip vs Osteoarthritis: A Propensity Matched Pair Analysis. Arthroplast. Today.

[19] Rajan T., Garde A., Lavu M.S., Porto J.R., Kamath A.F. (2025). Total hip arthroplasty outcomes for developmental dysplasia of the hip versus primary osteoarthritis: Older age increases medical, not surgical complications. J. Clin. Orthop. Trauma.

[20] Mortazavi S.M.J., Ghadimi E., Ardakani M.V., Razzaghof M., Ghasemi M.A., Nili A., Vafaei A., Moharrami A., Rasta S. (2022). Risk factors of dislocation after total hip arthroplasty in patients with developmental dysplasia of the hip. Int. Orthop..

[21] Engesæter L.B., Engesæter I.Ø., Fenstad A.M., I Havelin L., Kärrholm J., Garellick G., Pedersen A.B., Overgaard S. (2012). Low revision rate after total hip arthroplasty in patients with pediatric hip diseases. Acta Orthop..

[22] Boyle M.J., Frampton C.M.A., Crawford H.A. (2012). Early results of total hip arthroplasty in patients with developmental dysplasia of the hip compared with patients with osteoarthritis. J. Arthroplast..

[23] Bus M.P.A., Gademan M.G.J., Fiocco M., Nelissen R.G.H.H., de Witte P.B. (2024). Pediatric hip disorders are not associated with an increased 10-year revision risk after total hip arthroplasty under the age of 55: Results from the Dutch Arthroplasty Register. Acta Orthop..

[24] Clark S.C., Wright B.H., Feroe A.G., Couch C.G., Taunton M.J., Hevesi M. (2026). Patient-Reported Outcome Measures After Direct Anterior Total Hip Arthroplasty Are Comparable Between Patients With Developmental Dysplasia of the Hip and Osteoarthritis: A Propensity-Matched Analysis. J. Am. Acad. Orthop. Surg..

[25] Ashraf A., Larson A.N., Maradit-Kremers H., Kremers W.K., Lewallen D.G. (2014). Hospital costs of total hip arthroplasty for developmental dysplasia of the hip. Clin. Orthop. Relat. Res..

[26] Chen G., Nie Y., Xie J., Cao G., Huang Q., Pei F. (2018). Gait Analysis of Leg Length Discrepancy—Differentiated Hip Replacement Patients With Developmental Dysplasia: A Midterm Follow-Up. J. Arthroplast..

[27] Kawasaki M., Hasegawa Y., Okura T., Ochiai S., Fujibayashi T. (2017). Muscle Damage After Total Hip Arthroplasty Through the Direct Anterior Approach for Developmental Dysplasia of the Hip. J. Arthroplast..

[28] Galea V.P., Laaksonen I., Donahue G.S., Fukui K., Kaneuji A., Malchau H., Bragdon C. (2018). Developmental Dysplasia Treated With Cementless Total Hip Arthroplasty Utilizing High Hip Center Reconstruction: A Minimum 13-Year Follow-up Study. J. Arthroplast..

[29] Gharanizadeh K., Ravanbod H., Poursalehian M., Medhat A., Aminian A., Rajei M., Hassanzadeh M., Abolghasemian M. (2025). High Risk of Venous Thromboembolism With Aspirin Prophylaxis After THA for High-riding Developmental Dysplasia of the Hip: A Retrospective, Comparative Study. Clin. Orthop. Relat. Res..

[30] Zhang S., Ma M., Kong X., Zhou Y., Chen J., Chai W. (2024). Robotic-assisted total hip arthroplasty in patients with developmental dysplasia of the hip. Int. Orthop..

[31] Wang L., Trousdale R.T., Ai S., An K.-N., Dai K., Morrey B.F. (2012). Dislocation After Total Hip Arthroplasty Among Patients With Developmental Dysplasia of the Hip. J. Arthroplast..

[32] Vigdorchik J.M. (2020). The use of robotic-assisted total hip arthroplasty in developmental dysplasia of the hip. Arthroplast. Today.

[33] Zeng W.-N., Liu J.-L., Wang F.-Y., Zhang X., Fan H.-Q., Chen G.-X., Guo L., Duan X.-J., Zhou Q., Yang L. (2017). Total hip arthroplasty for patients with Crowe type IV developmental dysplasia of the hip: Ten years results. Int. J. Surg..

[34] Wang D., Li L.-L., Wang H.-Y., Pei F.-X., Zhou Z.-K. (2017). Long-Term Results of Cementless Total Hip Arthroplasty With Subtrochanteric Shortening Osteotomy in Crowe Type IV Developmental Dysplasia. J. Arthroplast..

[35] Tu Q., Ding H.-W., Chen H., Shen J.-J., Miao Q.-J., Liu B., Yu G.-W., Huang X.-H., Zhu C.-R., Tang Y. (2022). Preliminary application of 3D-printed individualised guiding templates for total hip arthroplasty in Crowe type IV developmental dysplasia of the hip. Hip Int..

[36] Ors C., Caylak R., Togrul E. (2022). Total Hip Arthroplasty With the Wagner Cone Femoral Stem in Patients With Crowe IV Developmental Dysplasia of the Hip: A Retrospective Study. J. Arthroplast..

[37] Mu W., Yang D., Xu B., Mamtimin A., Guo W., Cao L. (2016). Midterm Outcome of Cementless Total Hip Arthroplasty in Crowe IV–Hartofilakidis Type III Developmental Dysplasia of the Hip. J. Arthroplast..

[38] Viamont-Guerra M.-R., Chen A.F., Stirling P., Nover L., Guimarães R.P., Laude F. (2020). The Direct Anterior Approach for Total Hip Arthroplasty for Severe Dysplasia (Crowe III and IV) Provides Satisfactory Medium to Long-Term Outcomes. J. Arthroplast..

[39] Ukaj S., Krasniqi S., Ukaj D., Dervishaj F., Dyla D., Muçaj S., Tahirbegolli B. (2025). Total hip arthroplasty for Crowe type IV developmental dysplasia of the hip using a dual mobility acetabular cup. Sci. Rep..

[40] Liu Z., Bell C.D., Ong A.C., Zhang J., Li J., Zhang Y. (2021). Clinical evaluation of direct anterior approach total hip arthroplasty for severe developmental dysplasia of the hip. Sci. Rep..

[41] Shen J., Sun J., Ma H., Du Y., Li T., Zhou Y. (2020). High Hip Center Technique in Total Hip Arthroplasty for Crowe Type II–III Developmental Dysplasia: Results of Midterm Follow-up. Orthop. Surg..

[42] Fukushi J.-I., Kawano I., Motomura G., Hamai S., Kawaguchi K.-I., Nakashima Y. (2018). Does hip center location affect the recovery of abductor moment after total hip arthroplasty?. Orthop. Traumatol. Surg. Res..

[43] Kim Y.H., Park J.W., Kim J.S. (2023). Contemporary total hip arthroplasty with dual-mobility cups in developmental dysplasia of the hip. J. Arthroplast..

[44] Sato K., Sato A., Okuda N., Matsubara M., Koga H. (2023). A propensity score-matched comparison between Mako robotic arm-assisted system and conventional technique in total hip arthroplasty for patients with osteoarthritis secondary to developmental dysplasia of the hip. Arch. Orthop. Trauma Surg..

[45] Yacovelli S., Abdelaal M., Fillingham Y., Sutton R., Madding R., Parvizi J. (2021). Prior Pelvic Osteotomy Affects the Outcome of Subsequent Total Hip Arthroplasty. J. Arthroplast..

[46] Osawa Y., Hasegawa Y., Seki T., Amano T., Higuchi Y., Ishiguro N. (2016). Significantly Poor Outcomes of Total Hip Arthroplasty After Failed Periacetabular Osteotomy. J. Arthroplast..

[47] Osawa Y., Seki T., Takegami Y., Kusano T., Ishiguro N., Hasegawa Y. (2019). Failed periacetabular osteotomy leads to acetabular defects during subsequent total hip arthroplasty. Arch. Orthop. Trauma Surg..

[48] Minoda Y., Kadowaki T., Kim M. (2006). Total hip arthroplasty of dysplastic hip after previous Chiari pelvic osteotomy. Arch. Orthop. Trauma Surg..

[49] Ikezaki T., Kawai T., Okuzu Y., Goto K., Kuroda Y., Matsuda S. (2024). Effects of prior shelf procedure on subsequent conversion total hip arthroplasty. BMC Musculoskelet. Disord..

[50] Benad K., Martinot P., Dartus J., Girard J., Putman S., Migaud H. (2022). Influence of shelf acetabuloplasty on the outcomes of total hip arthroplasty in hips with dysplasia: A case-control study. Int. Orthop..

[51] Asai H., Osawa Y., Takegami Y., Funahashi H., Imagama S. (2026). Minimum 10-Year Outcomes of Total Hip Arthroplasty After Periacetabular Osteotomy. J. Arthroplast..

[52] Holzapfel B.M., Greimel F., Prodinger P.M., Pilge H., Nöth U., Gollwitzer H., Rudert M. (2012). Total hip replacement in developmental dysplasia using an oval-shaped cementless press-fit cup. Int. Orthop..

[53] Matsushita Y., Otani T., Hayama T., Fujii H., Kawaguchi Y., Saito M. (2022). A Modified Modular Stem in Primary Total Hip Arthroplasty for Developmental Dysplasia of the Hip: Average 11-year Follow-Up in Cases With Previously Reported 3-year Clinical Results. J. Arthroplast..

[54] Kristoffersson E., Otten V., Crnalic S. (2021). The accuracy of digital templating in cementless total hip arthroplasty in dysplastic hips. BMC Musculoskelet. Disord..

[55] Fahlbusch H., Budin M., Volk A., von Rehlingen Prinz F., Linke P., Citak M., Gehrke T., Ohlmeier M. (2023). Long-term outcomes of total hip arthroplasty in patients with developmental dysplasia of the hip: A minimum 21-year follow-up. Arch. Orthop. Trauma Surg..

[56] Di Martino A., Castagnini F., Stefanini N., Bordini B., Geraci G., Pilla F., Traina F., Faldini C. (2021). Survival rates and reasons for revision of different stem designs in total hip arthroplasty for developmental dysplasia: A regional registry study. J. Orthop. Traumatol..

[57] Castagnini F., Cosentino M., Bordini B., Montalti M., Biondi F., Faldini C., Traina F. (2023). Titanium modular stems in total hip arthroplasty due to developmental dysplasia: A registry comparison with single-taper implants. Hip Int..

[58] DeNovio A.C.-J., Bedard N.A., Trousdale R.T., Abdel M.P., Hannon C.P., Berry D.J. (2025). Long-Term Outcomes of Total Hip Arthroplasty With Subtrochanteric Osteotomy for Crowe IV Dysplasia. J. Arthroplast..

[59] Colo E., Rijnen W.H.C., Gardeniers J.W.M., van Kampen A., Schreurs B.W. (2016). Satisfying results of primary hip arthroplasty in patients with hip dysplasia at a mean followup of 20 years. Clin. Orthop. Relat. Res..

[60] Caylak R., Ors C., Togrul E. (2021). Minimum 10-Year Results of Cementless Ceramic-On-Ceramic Total Hip Arthroplasty Performed With Transverse Subtrochanteric Osteotomy in Crowe Type IV Hips. J. Arthroplast..

[61] Biant L.C., Bruce W.J., Assini J.B., Walker P.M., Walsh W.R. (2009). Primary total hip arthroplasty in severe developmental dysplasia of the hip. J. Arthroplast..

[62] Berninger M.T., Hungerer S., Friederichs J., Stuby F.M., Fulghum C., Schipp R. (2019). Primary Total Hip Arthroplasty in Severe Dysplastic Hip Osteoarthritis With a Far Proximal Cup Position. J. Arthroplast..

[63] Pai F.Y., Ma H.H., Chou T.A., Huang T.W., Chen C.F. (2021). Risk factors and modes of failure in the modern dual-mobility implant: A systematic review and meta-analysis. BMC Musculoskelet. Disord..

[64] Ding Z.C., Zhang B., Yang M.H., Zhou Y.G. (2020). Risk of dislocation after total hip arthroplasty in patients with Crowe type IV developmental dysplasia of the hip. Orthop. Surg..

[65] Flick T.R., Ross B.J., Sherman W.F. (2021). Instability After Total Hip Arthroplasty and the Role of Advanced and Robotic Technology. Orthop. Clin. North Am..

[66] Ciriello V., D’Apolito R., Sculco P.K., Sculco T.P. (2023). Is modular dual-mobility superior to standard bearings for reducing dislocation after primary total hip arthroplasty?. J. Clin. Med..

[67] Berry D.J., von Knoch M., Schleck C.D., Harmsen W.S. (2004). The cumulative long-term risk of dislocation after primary total hip arthroplasty. J. Bone Jt. Surg. Am. Vol..

[68] Hartofilakidis G., Karachalios T. (2004). Total hip arthroplasty for congenital hip disease. J. Bone Jt. Surg. Am. Vol..

[69] Yang J., Bryan A.J., Drabchuk R., Tetreault M.W., Calkins T.E., Della Valle C.J. (2022). Use of a monoblock dual-mobility acetabular component in primary total hip arthroplasty in patients at high risk of dislocation. Hip Int..

[70] Sloan M., Sheth N.P. (2018). Projected volume of primary total joint arthroplasty in young patients. J. Bone Jt. Surg. Am. Vol..

[71] Howie D.W., Holubowycz O.T., Middleton R., Large Articulation Study Group (2012). Large femoral heads decrease the incidence of dislocation after total hip arthroplasty: A randomized controlled trial. J. Bone Jt. Surg. Am. Vol..

[72] Pitz-Gonçalves L.I., Deckard E.R., Meneghini R.M. (2023). Large femoral heads and select dual-mobility bearings are associated with reduced instability in contemporary posterior approach total hip arthroplasty. J. Arthroplast..

[73] Jameson S.S., Baker P.N., Mason J., Rymaszewska M., Gregg P.J., Deehan D.J., Reed M.R. (2013). Independent predictors of failure up to 7.5 years after 35 386 single-brand cementless total hip replacements. Bone Jt. J..

[74] Heckmann N., McKnight B., Stefl M., Trasolini N.A., Ike H., Dorr L.D. (2018). Late Dislocation Following Total Hip Arthroplasty. J. Bone Jt. Surg..

[75] Lewinnek G.E., Lewis J.L., Tarr R., Compere C.L., Zimmerman J.R. (1978). Dislocations after total hip-replacement arthroplasties. J. Bone Jt. Surg. Am. Vol..

[76] Kayani B., Konan S., Tahmassebi J., Pietrzak J.R.T., Haddad F.S. (2019). Robotic-arm assisted total hip arthroplasty improves accuracy of acetabular component placement. Bone Jt. J..

[77] Lachiewicz P.F., Soileau E.S. (2013). Changing indications and results of dual-mobility components. J. Am. Acad. Orthop. Surg..

[78] Evans J.T., Evans J.P., Walker R.W., Blom A.W., Whitehouse M.R., Sayers A. (2019). How long does a hip replacement last?. Lancet.

[79] Bozic K.J., Kurtz S.M., Lau E., Ong K., Berry D.J. (2009). The epidemiology of revision total hip arthroplasty. J. Bone Jt. Surg. Am. Vol..

[80] Hauer G., Rasic L., Klim S., Leitner L., Leithner A., Sadoghi P. (2024). Septic complications are on the rise and aseptic loosening has decreased in total joint arthroplasty: An updated complication-based analysis using worldwide arthroplasty registers. Arch. Orthop. Trauma Surg..

[81] Feng X., Gu J., Zhou Y. (2022). Primary total hip arthroplasty failure: Aseptic loosening remains the most common cause of revision. Am. J. Transl. Res..

[82] Oltean-Dan D., Apostu D., Tomoaia G., Kerekes K., Păiuşan M.G., Bardas C.A., Benea H.R.C. (2022). Causes of revision after total hip arthroplasty in an orthopedics and traumatology regional center. Med. Pharm. Rep..

[83] Zijlstra W.P., de Hartog B., van Steenbergen L.N., Scheurs B.W., Nelissen R.G.H.H. (2017). Effect of femoral head size and surgical approach on risk of revision for dislocation after total hip arthroplasty. Acta Orthop..

[84] Schmucker A.M., Hupert N., Mandl L.A. (2019). The impact of frailty on short-term outcomes after elective hip and knee arthroplasty in older adults: A systematic review. Geriatr. Orthop. Surg. Rehabil..

[85] Qian H., Wang X., Wang P., Zhang G., Dang X., Wang K., Liu R. (2023). Total hip arthroplasty in patients with Crowe III/IV developmental dysplasia of the hip: Acetabular morphology and reconstruction techniques. Orthop. Surg..

[86] Greber E.M., Pelt C.E., Gililland J.M., Anderson M.B., Erickson J.A., Peters C.L. (2017). Challenges in total hip arthroplasty in the setting of developmental dysplasia of the hip. J. Arthroplast..

[87] Stirling P., Viamont-Guerra M.R., Strom L., Chen A.F., Saffarini M., Nover L., Laude F. (2021). Does cup position at the high hip center or anatomic hip center in total hip arthroplasty for developmental dysplasia of the hip result in better Harris Hip Scores and revision incidence? A systematic review. Clin. Orthop. Relat. Res..

[88] Hu Y., Zou D., Sun Q., Jiang M., Li H., Tsai T.Y., Zhang J. (2022). Postoperative hip center position associated with the range of internal rotation and extension during gait in hip dysplasia patients after total hip arthroplasty. Front. Bioeng. Biotechnol..

[89] Boughton O.R., Uemura K., Tamura K., Takao M., Hamada H., Cobb J.P., Sugano N. (2019). Gender and disease severity determine proximal femoral morphology in developmental dysplasia of the hip. J. Orthop. Res..

[90] Satılmış A.B., Cengiz T., Ülker A., Mutlu T. (2025). The impact of femoral anteversion correction on clinical outcomes in total hip arthroplasty for adult developmental dysplasia of the hip. J. Clin. Med..

[91] Myers C.A., Huff D.N., Mason J.B., Rullkoetter P.J. (2022). Effect of intraoperative treatment options on hip joint stability following total hip arthroplasty. J. Orthop. Res..

[92] Vigdorchik J.M., Sharma A.K., Madurawe C.S., Elbuluk A.M., Baré J.V., Pierrepont J.W. (2020). Does prosthetic or bony impingement occur more often in total hip arthroplasty? A dynamic preoperative analysis. J. Arthroplast..

[93] Ullmark G. (2017). The unstable total hip arthroplasty. EFORT Open Rev..

[94] Li W.T., Kozick Z., Sherman M., Restrepo C., Smith E.B., Courtney P.M. (2020). Dual mobility bearing articulations result in lower rates of dislocation after revision total hip arthroplasty. J. Am. Acad. Orthop. Surg..

[95] Pituckanotai K., Arirachakaran A., Tuchinda H., Putananon C., Nualsalee N., Setrkraising K., Kongtharvonskul J. (2018). Risk of revision and dislocation in single, dual mobility, and large femoral head total hip arthroplasty: A systematic review and network meta-analysis. Eur. J. Orthop. Surg. Traumatol..

[96] Neupane G., Madhusudhan R., Shrestha A., Vaishya R. (2020). Large diameter head in primary total hip arthroplasty: A systematic review. Indian J. Orthop..

[97] Garcia E.G., Prosser G.H., Bucher T.A. (2023). Pregnancy, hip pain, and total hip replacement. J. Bone Jt. Surg. Am. Vol..

[98] Lally L., Mandl L.A., Huang W.T., Goodman S.M. (2015). Pregnancy does not adversely affect postoperative pain and function in women with total hip arthroplasty. J. Clin. Rheumatol..

[99] Stea S., Bordini B., De Clerico M., Traina F., Toni A. (2007). Safety of pregnancy and delivery after total hip arthroplasty. J. Women’s Health.

[100] Meldrum R., Feinberg J.R., Capello W.N., Detterline A.J. (2003). Clinical outcome and incidence of pregnancy after bipolar and total hip arthroplasty in young women. J. Arthroplast..

[101] Yazici Y., Erkan D., Zuniga R., Bateman H., Salvati E.A., Magid S.K. (2003). Pregnancy outcomes following total hip arthroplasty: A preliminary study and review of the literature. Orthopedics.

[102] Oliviero A., Aicale R., Maffulli N. (2022). Pregnancy and parturition after hip arthroplasty. Surgeon.

[103] Drobniewski M., Gonera B., Olewnik Ł., Borowski A., Ruzik K., Triantafyllou G., Borowski A. (2024). Challenges and long-term outcomes of cementless total hip arthroplasty in patients under 30: A 24-year follow-up study with a minimum 8-year follow-up, focused on developmental dysplasia of the hip. J. Clin. Med..

[104] Chaudhry F., Bridger A., Daud A., Greenberg A., Safir O.A., Gross A.E., Kuzyk P.R. (2025). Retrospective review of outcomes of total hip arthroplasty in developmental dysplasia of the hip in adults. J. Arthroplast..

[105] Seo L.J., Gabor J., Novikov D., Feng J.E., Schwarzkopf R., Vigdorchik J.M. (2019). Outcomes in 385 developmental dysplastic hips requiring total hip arthroplasty. Arch. Orthop. Trauma Surg..

[106] Esmaeili S., Ghaseminejad-Raeini A., Ghane G., Soleimani M., Mortazavi S.M.J., Shafiei S.H. (2024). Total hip arthroplasty in patients who have Crowe type IV developmental dysplasia of the hip: A systematic review. J. Arthroplast..

[107] Bayliss L.E., Culliford D., Monk A.P., Glyn-Jones S., Prieto-Alhambra D., Judge A., Cooper C., Carr A.J., Arden N.K., Beard D.J. (2017). The effect of patient age at intervention on risk of implant revision after total replacement of the hip or knee: A population-based cohort study. Lancet.

[108] Pidhaietskyi V., Pidhaietskyi M. (2024). Systematisation of the causes that required revision hip replacement, methods of their solution, treatment results in Ukraine. BMC Surg..

[109] De Martino I., Triantafyllopoulos G.K., Sculco P.K., Sculco T.P. (2014). Dual mobility cups in total hip arthroplasty. World J. Orthop..

[110] Dorr L.D., Malik A., Dastane M., Wan Z. (2009). Combined anteversion technique for total hip arthroplasty. Clin. Orthop. Relat. Res..

[111] Baethge C., Goldbeck-Wood S., Mertens S. (2019). SANRA—A scale for the quality assessment of narrative review articles. Res. Integr. Peer Rev..

[112] Ioannidis J.P.A. (2016). Why most clinical research is not useful. PLoS Med..

[113] Swarup I., Marshall A.C., Lee Y.Y., Figgie M.P. (2016). Implant survival and patient-reported outcomes after total hip arthroplasty in young patients with developmental dysplasia of the hip. Hip Int..

[114] Dudkiewicz I., Salai M., Ganel A., Blankstein A., Chechik A. (2002). Total hip arthroplasty in patients younger than 30 years of age following developmental dysplasia of hip (DDH) in infancy. Arch. Orthop. Trauma Surg..

[115] Tokunaga K., Aslam N., Zdero R., Schemitsch E.H., Waddell J.P. (2011). Effect of prior Salter or Chiari osteotomy on THA with developmental hip dysplasia. Clin. Orthop. Relat. Res..

[116] Crnogaca K., Sulje Z., Delimar D. (2022). Previous corrective osteotomies of femur and pelvis are a risk factor for complications following total hip arthroplasty in hip dysplasia. J. Orthop..

[117] Bernasek T.L., Haidukewych G.J., Gustke K.A., Hill O., Levering M. (2007). Total hip arthroplasty requiring subtrochanteric osteotomy for developmental hip dysplasia: 5- to 14-year results. J. Arthroplast..

[118] Zhang J., Wang L., Mao Y., Li H., Ding H., Zhu Z. (2014). The use of combined anteversion in total hip arthroplasty for patients with developmental dysplasia of the hip. J. Arthroplast..

[119] Zhang J., Wei J., Mao Y., Li H., Xie Y., Zhu Z. (2015). Range of hip joint motion in developmental dysplasia of the hip patients following total hip arthroplasty with the surgical technique using the concept of combined anteversion: A study of Crowe I and II patients. J. Arthroplast..

[120] Shahbazi P., Jalilvand A.H., Ghaseminejad-Raeini A. (2023). Risk factors for dislocation following total hip arthroplasty in developmental dysplasia of the hip: A systematic review and meta-analysis. Int. Orthop..

[121] Hayashi S., Hashimoto S., Kuroda Y., Nakano N., Matsumoto T., Ishida K., Shibanuma N., Kuroda R. (2021). Robotic-arm assisted THA can achieve precise cup positioning in developmental dysplasia of the hip: A case control study. Bone Jt. Res..

[122] Liu Z.Y., Zhang J., Wu S.T., Li Z.Q., Xu Z.H., Zhang X., Zhou Y., Zhang Y. (2020). Direct anterior approach in Crowe type III-IV developmental dysplasia of the hip: Surgical technique and 2 years follow-up from Southwest China. Orthop. Surg..

[123] Kaiser D., Ried E., Zingg P.O., Rahm S. (2022). Acetabular reconstruction with femoral head autograft in primary total hip arthroplasty through a direct anterior approach is a reliable option for patients with secondary osteoarthritis due to developmental dysplasia of the hip. Arch. Orthop. Trauma Surg..

[124] Palumbo B.T., Salomon K., Sullivan A., Simon P., Lyons S., Bernasek T.L. (2022). Total hip arthroplasty with subtrochanteric osteotomy for developmental hip dysplasia: A long-term follow-up study. Arthroplast. Today.

[125] Solarino G., Vicenti G., Piazzolla A., Maruccia F., Notarnicola A., Moretti B. (2021). Total hip arthroplasty for dysplastic coxarthrosis using a cementless Wagner Cone stem. J. Orthop. Traumatol..

[126] Lari A., Ayyad S.H., Khan T., Alemairi T., El-Bakoury A., Hammad A.S. (2026). Outcomes and Complications of Total Hip Arthroplasty for Crowe Type IV Dysplasia of the Hip: An International Multicenter Study. J. Arthroplast..

[127] Chai W., Xu C., Guo R.-W., Kong X.-P., Fu J., Tang P.-F., Chen J.-Y. (2022). Does robotic-assisted computer navigation improve acetabular cup positioning in total hip arthroplasty for Crowe III/IV hip dysplasia? A propensity score case-match analysis. Int. Orthop..

