# Supplementary material for: Long-Term Prosthesis Survival After Total Hip Arthroplasty in Young Women with Developmental Dysplasia of the Hip: A Narrative Review of Instability, Revision Risk, and Reproductive Health Considerations
Source: J Clin Med. 2026 Sep 17;15(18):7220. doi: 10.3390/jcm15187220 (PMC13607231; doi:10.3390/jcm15187220)
Supplement: Supplementary file 1 [file jcm-15-07220-s001.zip › jcm-4521851-supplementary.pdf]

Supplementary Table S1. Characteristics of included studies (women ≤50 undergoing THA for DDH vs non-DDH).

First author (Year)	Country / Data source	Study design	Study period (years of THA)	N (women ≤50)	Age metric (mean/median; range)	DDH definition (clinical/radiographic/registry)	DDH severity reported? (Crowe/Hartofilakidis/other)	Non-DDH comparator group(s)	Follow-up (f/u; mean/median; range)	Implant era / key constructs (e.g., DM use, head size range, bearing)	Notes (key inclusion/exclusion; overlaps)
Zhang 2024[85]	China; single-center joint registry	Retrospective propensity-matched cohort	2018–2022	NR (mean age ~47; majority female)	Mean ± SD (~47 years)	Radiographic	Y; Crowe I–IV	None (DDH only; robotic vs manual)	≥6 months; mean/median NR	Cementless THA; MAKO robotic; Trident cup; Accolade II/Trilock/S-ROM stems; DM/head size NR	Single-center; DDH only; includes Crowe I–IV; no non-DDH controls
Zeng 2017 [101]	China; single-center tertiary hospital	Retrospective case series	2005–2008	NR (37/45 female; mean age 40.6)	Mean 40.6 (range 18–62)	Radiographic	Y; Crowe IV [illegible]	None	Mean 9.8 years (ra[illegible]	Cementless THA; Pinnacle cup; S-ROM stem; routine subtrochanteric shortening osteotomy; ceramic bearings	Crowe IV only; all underwent femoral shortening osteotomy; no control group; 10-yr outcomes
Yacovelli 2021 [112]	USA; single-center academic institution	Retrospective propensity-matched cohort (1:3)	2000–2018	NR (87.[illegible]	Mean ± SD: 38.0 (prior PO), 41.7 (controls)	Radiographic DDH with OA	Y; Crowe I–II only	None (DDH with vs without prior pelvic osteotomy)	Mean 3.8 years (±2–3)	Cementless THA; mixed manufacturers; ~32 mm heads; mostly polyethylene liners; limited DM	Prior pelvic osteotomy vs none; excludes Crowe III–IV; higher reoperation rate after PO
Wang 2017 [102]	China; single-center tertiary	Retrospective cohort (prospecti	2002–2010	NR explicitly; 49/62 women;	Mean 46.5 years (range 19–73)	Radiographic DDH	Y; Crowe IV (Hartofilakidis type III) only	None		Cementless THA; Pinnacle acetabular cup; S-ROM modular femoral stem; transverse subtrochanteric	Crowe IV only; all underwent femoral shortening osteotomy; cup placed at anatomic

	academic hospital (West China Hospital)	vely followed case series)		mean age 46.5					Mean 10 years (range 6.6–13.2)	shortening osteotomy; ceramic-on-ceramic (majority) and metal-on-poly bearings; head sizes 28–36 mm	hip center; no control group; long-term survivorship reported
Wang 2012 [100]	China & USA; single-center joint registry (Shanghai Ninth People's Hospital with Mayo Clinic collaboration)	Retrospective registry-based cohort study	2000–2009	NR explicitly; 611/820 female; mean age ~50	Mean ± SD: 49.8 ± 13.1 years (range 12–86)	Radiographic DDH with OA	Y; Crowe I–IV	None (DDH only)	Mean ~3 years; minimum 6 months	Cementless cups; mixed femoral stems (including S-ROM); head sizes 22, 28, ≥32 mm; focus on head size and component anteversion; DM not reported	Large DDH-only cohort (820 hips); primary outcome = dislocation; no difference by Crowe severity or subtrochanteric osteotomy; larger head size (≥32 mm) significantly reduced dislocation; anterior dislocation predominant (≈70%)
Vigdorchik 2020 [84]	USA; two high-volume academic centers (Hospital for Special Surgery & NYU Langone)	Retrospective multicenter case series (Level III)	2010–2015	NR explicitly; 56/79 female; mean age 45	Mean 45 years (range 26–64)	Radiographic DDH; CT-based 3D templating	Y; Crowe I–IV and Hartofilakidis A–C (III–IV analyzed together; no subgroup analysis)	None	Mean 3.1 years (range 2.0–4.6)	Cementless THA; MAKO robotic-assisted system; S-ROM modular femoral stems (all cases); CT-based planning; head size & bearing type planned but not stratified; DM not reported	DDH only; primary THA; revision cases excluded; focus on component positioning, ROM, leg-length discrepancy; no complications or revisions at short-term f/u
Viamont-Guerra 2020 [106]	France (with Brazil/US collaborators); single-	Retrospective consecutive case series (Level IV)	1993–2016	NR explicitly; 22/29 hips female; mean age ~49	Mean ± SD: 49 ± 16 years (range 17–75)	Radiographic DDH with secondary OA	Y; Crowe III–IV only	None	Mean 8.4 years (range 2–20)	Mixed cementless/cemented THA; direct anterior approach on traction table; true acetabulum placement; femoral head autograft in ~90%; subtrochanteric shortening osteotomy in	Severe DDH only; primary THA; no dislocations or infections; revisions for late PE wear/loosening; long-term functional improvement;

	center tertiary referral hospital									~17%; mixed cups/stems; polyethylene liners predominated; DM not reported	heterogeneous implants due to long study period
Ukaj 2025 [107]	Kosovo; two centers (University Clinical Center of Kosovo & Otrila Hospital)	Prospective case series	2016–2022	NR explicitly; 19/22 female; mean age ~49	Mean ± SD: 49.2 ± 12.1 years	Radiographic DDH	Y; Crowe IV only	None	Minimum 6 months (primary outcomes at 6 months)	Cementless THA; posterior approach; dual-mobility acetabular cup (Quattro); anatomic hip center placement; universal subtrochanteric femoral shortening osteotomy with compression plating; HA-coated femoral stem; head size NR	Unilateral Crowe IV only; no prior osteotomies; no dislocations; 100% negative Trendelenburg by 6 months; short-term f/u; small cohort; focus on DM to reduce instability
Tu 2022 [103]	China; single-center tertiary military hospital	Retrospective consecutive case series	2007–2015	NR explicitly; 8/12 female; mean age 33	Mean 33.3 years (range 20–46)	Radiographic DDH	Y; Crowe IV only	None	Mean 72.4 months (range 38–135)	Cementless THA; posterolateral approach; true acetabulum placement; 3D-printed individualized guiding templates (acetabulum + femoral osteotomy); Z-shaped subtrochanteric shortening osteotomy in 7/12 hips; structural bone grafting as needed; head size/bearing NR; DM not reported	Severe unilateral Crowe IV only; CT-based preop simulation; one early dislocation and one transient sciatic nerve palsy; no loosening or revisions at final f/u; no control group
Thillemann 2008 [26]	Denmark; nationwide Danish Hip Arthroplasty Registry	Registry-based retrospective cohort study	1995–2006	NR exp	Age categories reported (10–49, 50–59, 60–69, 70–79, ≥80); mean/median NR	Registry diagnosis of acetabular dysplasia (childhood hip disorder)	No (Crowe/Hartofilakidis not available in registry data)	Y; primary osteoarthritis THA (reference cohort)	Mean 4.6 years (range 1 day–12 years); early (0–6 mo) and late (0.5–12 yr) analyses	Mixed cemented, cementless, and hybrid fixation; femoral head sizes mainly 28–30 mm (since 1997); ~18% of DDH cases >30 mm; bearings not specified	Large population-level study; outcome = revision risk; acetabular dysplasia associated with ↑ early revision due to dislocation (RR≈1.9 overall; RR≈2.8 for dislocation); no increased long-term revision risk; DDH analyzed as registry diagnosis without radiographic severity

Solarino 2021 [126]	Italy; single-center tertiary referral hospital	Retrospective consecutive case series (Level IV)	1993–2016	NR explicitly; 63/87 patients female; mean age 53	Mean 53 years (range 27–88)	Radiographic DDH with secondary osteoarthritis	Y; Crowe I–III (Crowe IV excluded)	None	Mean 11.7 years (range 2.2–21.8)	Cementless THA; direct lateral approach; Wagner cone monoblock femoral stem (Zimmer-Biomet); hemispherical cementless cups (mostly Zimmer-Biomet); head sizes 28–36 mm; ceramic-on-poly, metal-on-poly, ceramic-on-ceramic bearings; no routine osteotomy	DDH Crowe I–III only; focus on femoral stem survivorship; no stem revisions for aseptic/septic loosening; no dislocations; heterogeneous implants due to long study period; Crowe IV hips excluded
Siddiqi 2020 [91]	USA An	Registry-based retrospective propensity-matched cohort study	2008–2016	NR explicitly; DDH cohort 603 patients (≈73.5% female); matched cohort 557 DDH	Mean ± SD (matched): 51.2 ± 11.6 years (DDH); age categories reported (<70 vs ≥70)	Registry diagnosis via ICD-9/ICD-10 codes (congenital hip dislocation, hip dysplasia, pelvic malformation)	No (severity not stratifiable in NSQIP)	Y; primary osteoarthritis THA (propensity-matched 1:1)	30-day postoperative outcomes only	Mixed contemporary THA (national registry); implant type, fixation, head size, bearing, DM use not available	Large national registry; excludes Perthes & SCFE; outcomes focus on operative time, transfusion, LOS, 30-day complications; DDH associated with longer OR time and higher transfusion rate; true risk of severe DDH likely underestimated due to lack of severity data
Shen 2020 [109]	China; single-center tertiary academic hospital (PLA General Hospital, Beijing)	Retrospective consecutive case series	2003–2013	NR explicitly; 54/69 patients female; mean age ~46	Mean ± SD: 46.5 ± 12.6 (Group A), 46.4 ± 11.0 (Group B)	Radiographic DDH	Y; Crowe II–III only	None (within-cohort comparison by hip center height)	Mean 8.9 ± 1.8 years (range 6.0–14.1)	Cementless THA; high hip center technique (≥22 mm above inter-teardrop); posterolateral approach; Betacup/Duraloc/Pinnacle/Trident cups; Corail/S-ROM/other stems; predominantly ceramic-on-ceramic bearings (~92%); no routine bone graft	Crowe II–III DDH only; compared moderate vs higher hip center (22–28 mm vs ≥28 mm); no difference in midterm function or survivorship; 2 revisions total (1 dislocation, 1 osteolysis); supports HHC as alternative to true acetabulum reconstruction
Sato 2022 [111]	Japan; single-center academic hospital	Retrospective propensity score–matched cohort	2019–2020	NR explicitly; matched cohorts 84 Mako	Mean ± SD: 66 ± 8 years (both groups); age range NR	Radiographic DDH with secondary osteoarthritis	Y; Crowe I–II predominantly (Crowe III–IV excluded)	None (comparison: robotic-assisted vs manual THA within DDH)	Short-term; postoperative CT at 3 months (no mid/long-term f/u)	Cementless THA; MAKO robotic-arm assisted system vs conventional technique; uniform implants (Accolade II femoral stem, Trident acetabular cup); lateral	DDH with OA only; excluded non-DDH diagnoses and other implant systems; primary outcome = accuracy of cup

	(Nissan Tamagawa Hospital, Tokyo)	study (1:1)		/ 84 manual; 83/84 female in each group						(modified Watson-Jones) approach; head size/bearing not specified; DM not reported	inclination, anteversion, and center of rotation on CT; no dislocations or complications reported at early f/u
Rajan 2025 [92]	USA; national multi-institutional database (TriNetX research network)	Registry-based retrospective propensity-matched cohort study	2014–2023	DDH cohort age 18–50: n = 2802 pre-match (≈59% female); matched n = 2701	Mean ± SD: 45.1 ± 8.1 y (DDH 18–50); comparison group ≥50 y mean 66.2 ± 8.2	Administrative diagnosis of OA secondary to DDH (ICD-9/10 codes)	No (Crowe/Hartofilakidis not available in database)	Y; primary osteoarthritis THA (propensity-matched)	90 days, 1 year, and 2 years postoperative	Contemporary THA; implant fixation, head size, bearing, DM use not available in registry	Largest DDH THA cohort to date; compares DDH vs primary OA and age-stratified DDH (<50 vs ≥50); DDH associated with ↑ dislocation and heterotopic ossification vs OA; younger DDH patients had fewer medical but similar surgical complications; limited by lack of radiographic severity and implant data
Osawa 2019 [114]	Japan; single-center academic hospital (Nagoya University)	Retrospective matched cohort study with CT-based simulation	2010–2017	NR explicitly; 49/55 patients female; mean age ~57	Mean ± SD: 56.6 ± 6.4 years (range 27–80)	Radiographic DDH with secondary osteoarthritis after PAO	Y; Crowe I–III (Crowe II–III analyzed together)	Y; primary THA for hip dysplasia without prior osteotomy (matched by age, sex, Crowe)	Preoperative CT-based simulation study (no clinical f/u outcomes)	CT-based socket placement simulation; anatomical vs elevated hip center (+10 mm); standardized cup orientation (45° inclination, 20° anteversion); no clinical implant survivorship data	Focus on acetabular wall defects after failed PAO; THA after PAO associated with significantly reduced anterior, posterior, and superior acetabular sector angles and medial wall thickness vs controls; elevation of hip center improved socket coverage; morphologic/technical study rather than outcomes-based
Osawa 2016 [113]	Japan; single-center academic hospital	Retrospective matched case–control study	2000–2014	NR explicitly; osteotomy group	Mean ± SD: 56.5 ± 6.4 years (range 44–74)	Radiographic DDH with secondary osteoarthritis	Y; Crowe I–II (control group restricted to Crowe I–II)	Y; primary THA for DDH without prior osteotomy (matched by age, sex, time of surgery)	Mean 5.4 years (range 1–14)	Mixed cementless and cemented THA; posterior approach; target cup position 45° inclination / 20° anteversion; Trident/TriAD/Secur-Fit	Conversion THA after failed PAO (ERAO/RAO); concomitant valgus osteotomy excluded; poorer HHS, ROM, and

	(Nagoya University)			46/48 female; mean age ~56						cups; Super Secur-Fit, Exeter, Omnifit stems; head sizes ~48–49 mm; bearings mainly ceramic-on-ceramic or metal/ceramic-on-poly; no DM	Lewinnek safe-zone placement vs primary THA; no revisions; 1 dislocation in each group; highlights technical difficulty and inferior functional outcomes after PAO
Ors 2022 [104]	Turkey; single-center private tertiary orthopedic hospital	Retrospective consecutive case series	2011–2015	NR explicitly; 81/91 patients female; mean age 43	Mean ± SD: 43.0 ± 13.8 years	Radiographic DDH	Y; Crowe IV only	None	Mean 8.4 years (range 6–10.4 years; 72–125 months)	Cementless THA; Wagner cone femoral stem; Trilogy IT acetabular cup; posterolateral approach; transverse subtrochanteric shortening osteotomy in all hips; autograft augmentation of osteotomy site; head sizes 28 mm (60%) and 32 mm (40%); ceramic bearings	Large Crowe IV-only cohort (127 hips); 55% intraoperative femoral cracks (cabled); no adverse effect on outcomes; dislocation rate 3.9%; 10-yr survivorship 94.5% (any component) and 96.9% (femoral); highlights tradeoff between rigid fixation and fracture risk
Mu 2016 [105]	China; single-center tertiary academic hospital (First Affiliated Hospital of Xinjiang Medical University)	Retrospective consecutive case series	1999–2009	NR explicitly; 59/78 women initially; final cohort 58 patients (71 hips); mean age 35.8	Mean 35.8 years (range 16–56)	Radiographic DDH	Y; Crowe IV / Hartofilakidis type III only	None	Mean 70.5 months (~5.9 years; range 39–98 months)	Cementless THA; Zweymüller components (BICON-PLUS threaded acetabular cup, SL-PLUS rectangular stem; Smith & Nephew); true acetabulum placement; transverse subtrochanteric shortening osteotomy in 61/71 hips; head sizes 22 or 28 mm; conventional polyethylene liner	Crowe IV only; no control group; high intraoperative fracture rate (~28%); polyethylene wear and nerve palsy notable complications; 98-month survivorship 91.4%; emphasizes technical demands and trade-offs of Zweymüller implants in severe DDH
Mortazavi 2022 [18]	Iran; single tertiary referral academi	Retrospective cohort study	2014–2019	NR explicitly; DDH cohort 127/143	Mean ± SD: 42.2 ± 6.1 years (range 25–71)	Radiographic DDH	Y; Crowe I–IV	Y; primary OA and other etiologies (AVN, post-traumatic, inflammatory)	Mean 40.4 months (range 6–59 months)	Cementless hemispherical cups (Continuum, Trilogy, Trident, Pinnacle); multiple femoral stems (Fitmore, Wagner SL, Wagner Cone,	Focused on post-THA dislocation risk in DDH; overall DDH dislocation rate 12%; independent predictors: higher

	c center (Tehran University of Medical Sciences)			female; mean age 42						M/L Taper, Accolade, Corail, Omnifit); direct anterior approach; neutral liners only; head sizes variable (<32 vs ≥32 mm); no dual mobility	Crowe grade (II–IV), intra-operative fracture, postoperative acetabular offset ≤16 mm; failure of offset restoration emphasized; femoral shortening osteotomy not independently associated with dislocation
Minoda 2006 [115]	Japan; single-center municipal hospital (Suita Municipal Hospital, Osaka)	Retrospective matched cohort study (Chiari osteotomy vs no prior surgery)	1998–2003	NR explicitly; 10/10 female in Chiari group; 20/20 female in control group	Mean ± SD: 55.4 ± 7.2 (Chiari), 56.3 ± 4.6 (control)	Radiographic DDH	Y; Crowe II–III (no Crowe I or IV included)	No (comparator = DDH without prior pelvic osteotomy)	Mean 3.0 years (range 2–5)	Cementless THA; posterolateral approach; Omnifit or Anca-Fit components; anatomic cup placement ± bulk femoral-head autograft fixed with PLLA screws; head size ~46 mm cups; DM not reported	Focus on biomechanical effects of prior Chiari pelvic osteotomy; longer operative time and greater blood loss in Chiari group; reduced joint force but more vertical joint force vector; no loosening or revisions at short-term f/u; clinical outcomes equivalent between groups
Matsushita 2022 [120]	Japan; single-center academic hospital (Jikei University School of Medicine, Tokyo)	Retrospective consecutive case series with long-term f/u	2006–2009	NR explicitly; 179/201 female at ≥5-year f/u; age <50 subgroup not stratified	Mean age at surgery 61.6 years (range 35–84)	Radiographic DDH with secondary osteoarthritis	Y; Crowe I–IV (Type I most common; Types III–IV minority)	None	Mean 137 months (~11.4 years; range 60–175 months)	Cementless THA; S-ROM-A modular femoral stem (DePuy); posterolateral approach with posterior soft-tissue repair; bearing surfaces mainly CoCr-on-polyethylene (CPE or HXLPE); head sizes mostly 28–36 mm; no dual mobility	Large DDH-only cohort (220 hips; 201 with ≥5-yr FU); focus on modular stem anteversion adjustment; stem survivorship 97.4% at 14 years; complications included mild osteolysis (~20%), distal stem fracture (1.5%), and rare trunnionosis; no association between large anteversion adjustment (±20°) and osteolysis
Liu 2021 [108]	China (Southwest China);	Retrospective comparative cohort	2016–2018	NR explicitly; 18/22 (82%)	Mean ± SD: 42.0 ± 13.6 years (DAA);	Radiographic DDH with secondary osteoarthritis	Y; Crowe III–IV only	No (comparator = DDH Crowe III–IV treated with	Mean 28.5 months (DAA; range 26–45) vs 39.0 months (PLA; range 26–48)	Cementless THA; DAA vs PLA; S-ROM modular stems (~95%); occasional Trilock/Corail; ceramic-on-	Focused on surgical approach for severe DDH; excluded infection,

	single-center academic hospital (Xinqiao Hospital, Army Medical University)	study (DAA vs posterolateral approach)		female in DAA cohort; mean age ~42	47.8 ± 17.6 years (PLA)			posterolateral approach)		ceramic (78%) or ceramic-on-poly; head sizes mainly 28–32 mm; true acetabular placement; transverse subtrochanteric shortening osteotomy in ~44% (DAA)	neuromuscular disease, unilateral THA in bilateral DDH; DAA showed greater HHS/WOMAC improvement, faster abductor/flexor strength recovery, improved limp recovery; no increase in dislocation or revision vs PLA; short-term f/u only
Lari 2025 [127]	Multinational; 4 centers in Kuwait, UK, UAE, and Egypt	Retrospective multicenter case series	2013–2022	29/37 women (78.4%); mean age 35 (majority ≤50)	Mean ± SD: 35 ± 11.9 years (range 20–60)	Radiographic DDH	Y; Crowe IV only	None	Mean 7.8 ± 2.8 years (range 3–12)	Predominantly cementless THA (94.6%); true acetabulum reconstruction; transverse subtrochanteric shortening osteotomy in 64.9% (mean 2.8 cm, usually plate-augmented); mixed surgical approaches (posterior 48.6%, lateral 40.5%, DAA 10.8%); mixed femoral stems (tapered 62%, conical 38%); head sizes median 28 mm (range 22–36); ceramic-on-poly most common; dual mobility 5.4%	Large contemporary Crowe IV series; unilateral and staged bilateral cases included; no dislocations or infections; complications in 21% of patients (mainly periprosthetic fracture, osteotomy nonunion); 97.3% implant survivorship at ~8 yrs; highlights variability in technique and higher complication burden despite good functional gains
Kristoffersson 2021 [121]	Sweden; single-center academic hospital (Umeå University Hospital)	Retrospective consecutive case series	2008–2015	NR explicitly; 27/50 patients female; age ≤50 not stratified	Median age NR; overall age range 23–68 years	Radiographic DDH (CE angle <25°)	Y; Crowe I–IV and Hartofilakidis A–C	None	Immediate postoperative radiographic f/u (1–3 days); no clinical mid/long-term f/u	Cementless THA; posterior approach; multiple acetabular cups (Pinnacle, Reflection, Trilogy, Continuum, etc.); predominantly Corail femoral stems; occasional Wagner cone, Synergy, custom stem; femoral-head autograft for acetabular coverage in ~13%; femoral osteotomy in 2 Crowe IV hips	Focus on accuracy of digital preoperative templating (component size, center of rotation, LLD); majority Crowe I hips (~75%); exact templating accuracy ~43% (cup) and ~38% (stem), improving to ~80% within ±1 size; morphologic/planning study rather than outcomes- or survivorship-focused

Kim 2023 [29]	South Korea; single-center academic hospital (Pusan National University Yangsan Hospital)	Retrospective comparative cohort study (DM vs fixed-bearing cups)	2015–2020	Not stratified by ≤50; overall 126/172 female (73.3%); mean age 54	Mean ± SD: 54.4 ± 12.3 years	Radiographic DDH with secondary osteoarthritis	Y; Crowe I–IV (Crowe I most common; IV rare ~3%)	No (comparison within DDH: dual-mobility vs fixed-bearing THA)	Minimum 2 years (mean ≈ 2 years)	Cementless THA; posterolateral approach; DM cups (G7 DM) vs fixed-bearing cups (Trilogy, G7); femoral stems: Microplasty, VerSys, Wagner cone (narrow canals); head sizes 28–36 mm (FB); third-generation highly cross-linked polyethylene DM liners; occasional structural bone graft	Excluded revision THA, infection, conversion THA, inadequate FU; primary outcome = dislocation (FB 9% vs DM 0%, p = 0.003); no difference in mHHS at 2 yrs; short-term f/u; supports DM use to reduce instability in dysplastic hips
Kawasaki 2017 [97]	Japan; single-center academic hospitals (Aichi Koseiren Konan Kosei Hospital / Nagoya University)	Prospective comparative imaging study	2013–2014	NR; DDH cohorts predominantly female (D1: 33/37; D2–3: 12/13); mean age >60	Mean ± SD: 66.8 ± 8.7 (Crowe I), 65.1 ± 7.9 (Crowe II–III), 59.1 ± 15.9 (controls)	Radiographic DDH with secondary osteoarthritis	Y; Crowe I, II–III	Y; osteonecrosis of femoral head treated with DAA THA	12 months (MRI and clinical)	Cementless THA; direct anterior approach; G7 acetabular cup (Biomet); Taperloc Complete Microplasty femoral stem; standard fixed-bearing articulation; no dual mobility	MRI-based muscle damage analysis (Gmini, TFL, obturator internus); excluded prior hip surgery and non-DDH OA; DDH associated with significant postoperative fatty atrophy and ↓ muscle CSA (esp. obturator internus) despite excellent HHS; morphologic/biologic study—no survivorship or revision outcomes
Kaiser 2022 [88]	Switzerland; single-center academic institution (Balgrist University Hospital, Zurich)	Retrospective consecutive case series	2006–2018	Not explicitly stratified; 17/24 patients female (19/29 hips); mean age 43	Mean age 43 years (range 18–75)	Radiographic DDH with secondary osteoarthritis	Y; Hartofilakidis grade A (n=19 hips) and B (n=10 hips); Crowe not reported	None	Mean 35 months (range 12–137 months); minimum 12 months	Cementless primary THA; direct anterior approach on traction table; acetabular reconstruction using bulk femoral head autograft fixed with screws; press-fit cups (Versafit, Fitmore); mixed femoral stems (Quadra-H, Fitmore, Exafit, CMK); head sizes 22 or 28 mm; dual mobility used in 1 elderly patient	DDH Hartofilakidis A–B only; excluded posterior/lateral approaches and cemented cups; no dislocations or acetabular loosening; 100% graft incorporation by 12 months; focus on restoring anatomic center of rotation via DAA with autograft

											augmentation; short- to mid-term outcomes only
Ikezaki 2024 [116]	Japan; single-center academic hospital (Kyoto University Hospital)	Retrospective propensity-matched cohort study (1:1)	Shelf group: 1997–2020; control group: 2006–2020	Shelf group: 40/46 female (87%); matched control: 39/46 female; age ≤50 not isolated	Mean ± SD: 56.6 ± 9.1 years (shelf) vs 56.4 ± 9.4 (control)	Radiographic DDH with secondary osteoarthritis	Y; Crowe I only (matched cohorts)	No (comparator = Crowe I DDH without prior surgery)	Mean radiographic FU: 9.2 ± 6.6 years (shelf) vs 6.0 ± 3.7 (control); minimum ≥1 year	Mixed cemented, cementless, and hybrid THA; lateral or anterolateral approach; anatomic hip center placement (40° inclination / 20° anteversion); frequent acetabular bone grafting (~87% shelf); no dual mobility	Conversion THA after prior shelf acetabuloplasty; no infections, dislocations, or revisions; shelf group had significantly lower cup CE angle and bone coverage index despite similar grafting rates; prior shelf did **not** improve acetabular bone coverage at THA
Holzapfel 2012 [119]	Germany; two academic orthopedic centers (University of Würzburg & Technical University of Munich)	Retrospective matched cohort study	1998–2008	Not stratified by ≤50; 28/40 female (cranial socket group), 32/40 female (control); mean age ~53	Mean age: 53.1 y (cranial socket) vs 52.3 y (control)	Radiographic DDH with acetabular roof deficiency	Y; Hartofilakidis type II only	No (comparator = DDH Hartofilakidis II treated with bulk femoral head autograft + hemispherical cup)	Mean 5.6 years (cranial socket; range 26–133 mo) vs 6.9 years (control; range 30–151 mo)	Cementless THA; cranially extended oval press-fit acetabular cup ("cranial socket") vs standard hemispherical press-fit cup + bulk femoral autograft; UHMWPE liners; head sizes 28–36 mm; minimally invasive anterolateral or anterior approach	Focus on acetabular roof deficiency reconstruction; no aseptic/septic loosening in either group; similar HHS and SF-36; shorter operative time with cranial socket; anatomical hip center restoration more accurate with bulk autograft; mid-term outcomes only
Gharanizadeh 2025 [99]	Iran; two arthroplasty centers (university-affiliated + private hospital, Tehran)	Retrospective comparative cohort study	June–November 2021	High-riding DDH: 39/47 female (83%); age ≤50 common but not isolated	Mean ± SD: 43 ± 15 years (high-riding DDH) vs 53 ± 17 (controls)	Radiographic DDH	Y; Crowe III–IV ("high-riding DDH")	Y; primary THA for other diagnoses (Crowe I–II DDH, OA, AVN, RA, fracture)	Minimum 3 months; outcomes assessed within 90 days post-op	Contemporary THA; lateral approach; true acetabulum placement; frequent shelf/impaction bone grafting; femoral shortening osteotomy in most Crowe IV; aspirin-only VTE prophylaxis (≈80–81 mg BID); implant specifics (head size, bearing, DM) not detailed	Focus on VTE risk; excluded prior VTE, chronic anticoagulation, bilateral THA, <3-mo FU; symptomatic VTE significantly higher in Crowe III–IV DDH (17% vs 1%); limb lengthening independently associated with VTE; authors recommend stronger prophylaxis

											than aspirin for high-riding DDH
Galea 2018 [98]	USA & Japan; single high-volume academic center (Massachusetts General Hospital / Harvard)	Prospective consecutive cohort study with long-term f/u	2000–2002	Not stratified by ≤50; overall 114/123 hips female (92.7%)	Mean age at surgery 55.2 years (range 33–74)	Radiographic DDH with secondary osteoarthritis	Y; Crowe I–IV	Y; nondysplastic hips and Crowe I vs Crowe II–IV (severity-based comparison)	Mean 13.8 years (range 13.0–15.5); 100% f/u	Cementless THA; high hip center (HHC) reconstruction at native/pseudoacetabulum; Trilogy acetabular shell + Longevity highly cross-linked polyethylene liner; 26 mm ceramic head; mostly Anatomic femoral stem; posterolateral approach; no dual mobility	Longest f/u HHC DDH series; excellent 13-yr PROMs (median HHS 91.9); 99.2% survivorship (1 femoral loosening); no dislocations or osteolysis; Crowe II–IV showed small ROM decline but similar pain/function vs Crowe I/OA; supports safety of HHC in DDH with modern HXLPE
Fukushi 2018 [110]	Japan; single-center academic hospital (Kyushu University Hospital)	Retrospective comparative cohort study	2012–2016	Not stratified by ≤50; 86/100 female; mean age 65.5	Mean ± SD: 65.5 ± 9.9 years (range 40–86)	Radiographic DDH (lateral center-edge angle <25°)	Y; Crowe I–III (Crowe I: 76, II: 15, III: 9; no Crowe IV)	None (within-DDH comparison by hip center height)	12 months postoperative (muscle testing at 1, 6, and 12 months)	Cementless THA; posterolateral approach with piriformis sparing; PerFix910 femoral stem + AMS acetabular cup (Kyocera); head sizes 26 mm (8%) and 32 mm (92%); no dual mobility	Focus on biomechanical recovery rather than survivorship; superior hip center placement >15 mm delayed abductor moment recovery at 6 months but not at 12 months; HHS improved from 51 → 94.5; excluded contralateral hip disease, neurologic disorders, RA/ONFH; Level III evidence
Fahlbusch 2023 [66]	Germany; single high-volume tertiary arthroplasty center (Helios ENDO-Klinik Hambur	Retrospective single-center cohort study with very long-term f/u	1997–2000	Not stratified by ≤50; overall 91/110 patients female (83%); mean age ~51	Mean ± SD: 51 ± 9.5 years (range 21–65)	Clinical + radiographic DDH confirmed intra-operatively	Y; Crowe I–IV (I 57%, II 27%, III 7%, IV 9%)	None	Mean 23 ± 1.3 years (range 21–25); minimum 21 years	Predominantly cemented THA; posterior approach; cemented Mark III polyethylene cup ± femoral-head autograft acetabuloplasty (47%); femoral shortening osteotomy in 10%; femoral stems: Mark III standard, dysplasia stems, or CFP short stem; ceramic (72%) or	Longest f/u DDH THA series; overall revision rate 18% (mostly aseptic loosening); 10-yr survivorship 98.3%, ~20-yr 84.2%, final 81.8%; dislocation rate 7%; worse survivorship with prior osteotomy and higher Crowe grade; highlights durability limits of older cemented

	g / UKE Hamburg)									metal heads; no dual mobility	reconstructions in young DDH patients
Engesaeter 2012 [25]	Denmark, Norway, Sweden; Nordic Arthroplasty Register Association (NARA)	Multinational registry-based retrospective cohort study	1995–2009	Not stratified by ≤50; DDH subgroup 12,068 THAs, 72.5% female; mean age 56	Mean ± SD: 55.1 ± 13 years (pediatric hip disease); age categories (<30 to >80)	Registry diagnosis of DDH (surgeon-reported; validated in prior registry studies)	No (Crowe/Hartofilakidis not available in registry data)	Y; primary osteoarthritis THA (288,435 hips)	Mean ~6.4 years for DDH (reverse Kaplan–Meier); up to 14 years	Mixed cemented, uncemented, hybrid, reverse-hybrid fixation; uncemented more common in DDH; head size, bearing, DM not available	Very large population-level study (370,630 THAs); after adjustment for age, sex, fixation, overall revision risk for pediatric hip disease ≈ OA; DDH had higher early (≤6 mo) revision risk driven by dislocation (RR ~1.9); no long-term revision difference vs OA; severity and surgical technique not capturable
Di Martino 2021 [122]	Italy; Emilia-Romagna regional arthroplasty registry (RIPO)	Registry-based retrospective cohort study	2000–2017	Not stratified by ≤50; total DDH THAs = 5,761; female ~71–83% depending on stem group	Mean age by cohort ≈ 58–63 years	Registry diagnosis of developmental dysplasia (surgeon-reported)	No (Crowe/Hartofilakidis not available)	None (comparisons among DDH stem designs only)	Mean 6.5–10.7 years depending on cohort; up to 17 years	Cementless THA only; stems stratified by design (anatomic, tapered wedge/rectangular, conical) and modularity; mixed bearings (CoC, CoP, MoP, some MoM); head sizes <36 mm vs ≥36 mm; mixed cups	Large registry stem-focused survivorship analysis; NM-CON stems had highest failure risk driven by cup aseptic loosening; dislocation and stem loosening rates similar across groups; lack of radiographic severity and technique detail limits causal inference
DeNovio 2025 [124]	United States; Mayo Clinic institutional total joint registry	Retrospective single-center cohort study (long-term f/u of previously	1992–2005	28 hips (24 patients); 83% female; mean age 48 (≤50 common but not isolated)	Mean 48 years (range 30–72)	Radiographic Crowe IV DDH	Y; Crowe IV only	None	Mean 18 years (range 12–28); survivorship to 20 years	Cementless THA with subtrochanteric shortening osteotomy (mostly transverse); posterior approach (93%); porous-coated acetabular cups with screws at anatomic hip center; femoral stems predominantly extensively porous-coated; structural femoral-head autograft in	Landmark ≥20-year Crowe IV series; 20-yr survivorship free of any revision 79%; revisions occurred ≤6 yrs only (mostly femoral aseptic loosening); no sciatic nerve injury; dislocation survivorship 85%; demonstrates durable long-term fixation with

		published series)								~36%; highly cross-linked polyethylene liners; no dual mobility	modern cementless THA + SSO
Colo 2016 [90]	Netherlands; single tertiary academic center (Radboud University Medical Center, Nijmegen)	Retrospective single-center case series with competing-risk analysis	1984–1995	24 hips (18 patients) with ≥15-yr FU; 17/18 patients female; 13 hips (54%) <50 yrs	Mean age 48 years (range 26–74)	Radiographic DDH confirmed intraoperatively	Y; Crowe I–IV (I = 5, II = 8, III = 9, IV = 2)	None	Mean 20 years (range 16–29); minimum 15 years	Cemented primary THA; acetabular impaction bone grafting with morselized auto/allograft ± metal mesh; cemented Elite (DePuy) or Müller cups; anatomic or elevated hip center; femoral components cemented; head size NR; no dual mobility	Long-term outcomes of cemented impaction bone grafting for DDH; cumulative cup revision 7% at 15–20 yrs (aseptic loosening 4%); no dislocations or infections; HHS improved 37 → 83; highlights durable acetabular reconstruction but small cohort and heterogeneous severity
Clark 2025 [95]	United States; Mayo Clinic institutional joint replacement registry	Retrospective propensity-matched cohort study (1:4)	2010–2022	Dysplasia: 38 hips (≈68% female); age ≤50 common but not isolated	Mean ± SD: 51.7 ± 13.4 yrs (DDH) vs 54.0 ± 9.0 yrs (OA)	Radiographic DDH	Y; Crowe I–IV (I 86.8%, II 5.3%, III 2.6%, IV 5.3%)	Y; primary osteoarthritis THA	Mean 5.2 ± 2.8 yrs (DDH) vs 5.1 ± 2.8 yrs (OA)	Cementless direct anterior THA; press-fit cups with screws (more screws in DDH); mixed collared/uncollared stems; mean head size ~35 mm; occasional acetabular bone grafting (5.2%) and femoral shortening osteotomy in Crowe IV; no dual mobility	PROM-focused study; excluded prior PAO, fracture, AVN, neurologic disease; PROMs (mHHS, HOOS-Pain, FJS-12, VAS) and revision rates comparable between DDH and OA; majority low-grade dysplasia limits inference for severe DDH
Chen 2018 [23]	China; single-center academic hospital (West China Hospital, Sichuan University)	Prospective cohort study with healthy control comparison	~2012–2013 (patients assessed at 5-year midterm f/u)	39/45 female (86.7%); age ≤50 not isolated (substantial ≤50 representation)	Mean ± SD: 55.1 ± 7.9 (LLD <10 mm), 48.0 ± 10.8 (LLD 10–20 mm), controls 51.3 ± 7.5	Radiographic DDH with secondary osteoarthritis	Y; Crowe II–III only	Y; healthy age-matched controls	Mean ~57 months (~4.8–5 years) postoperative	Cementless THA; posterolateral approach; DePuy acetabular cup + femoral stem; ceramic-on-ceramic bearing; no subtrochanteric shortening osteotomy	Focus on gait analysis and leg-length discrepancy (LLD); patients grouped by postoperative LLD (<10 mm vs 10–20 mm); no neurologic disease or prior hip surgery; despite LLD ≤20 mm, gait efficiency remained inferior to controls; LLD <10 mm associated with better hip ROM and

											bilateral gait symmetry; supports aggressive limb-length equalization in Crowe II–III DDH
Chai 2022 [128]	China; single high-volume academic center (PLA General Hospital, Beijing)	Retrospective propensity score–matched comparative cohort study (1:1)	January 2017 – August 2019	Matched cohort: 54 hips (27 robotic, 27 conventional); 100% female in matched groups; age ≤50 common but not isolated	Mean ± SD: 43.0 ± 8.9 yrs (robotic) vs 44.6 ± 9.5 yrs (conventional)	Radiographic DDH	Y; Crowe III–IV only	No (comparison = robotic-assisted vs conventional THA within Crowe III–IV DDH)	Minimum 2 years; clinical outcomes assessed at 1- and 2-year f/u	Cementless THA; posterolateral approach; robotic-assisted (Mako) vs manual; true acetabulum reconstruction when possible; frequent subtrochanteric shortening osteotomy; Trident cups (robotic) vs Pinnacle/Combicup (manual); modular S-ROM femoral stem; head size NR; no dual mobility	Focus on acetabular cup positioning accuracy; robotic THA achieved significantly higher Lewinnek/Callanan safe-zone placement (≈96% vs 37%); no difference in LLD, HHS, or WOMAC at 2 yrs; no dislocations or nerve injuries; short-term outcomes only; addresses technical precision rather than survivorship
Caylak 2021 [125]	Turkey; single-center tertiary orthopedic hospital	Retrospective consecutive case series	2004–2009	NR explicitly; 43/50 patients female; majority ≤50	Mean 41.5 years (range 23–61)	Radiographic DDH	Y; Crowe IV only	None	Mean 131.9 months (~11 years); minimum 10 years	Cementless THA; ceramic-on-ceramic bearings; true acetabulum reconstruction; transverse subtrochanteric femoral shortening osteotomy in all hips; mixed femoral stems (CDH, Synergy, Wagner Cone); head size NR; no dual mobility	Long-term Crowe IV series; significant improvements in HHS and WOMAC; femoral stem 10-yr survivorship 94%, acetabular 98.5%; complications included dislocation and intraoperative femoral fracture; no nerve palsies despite large limb lengthening
Castagnini 2022 [123]	Italy; Emilia-Romagna Regional Arthroplasty Registry (RIPO)	Registry-based retrospective comparative cohort study	2000–2018	Not stratified by ≤50; total DDH THAs = 6,429; female 73.7%	Mean age: 61.0 yrs (ST) vs 58.6 yrs (DT); age categories (<65 vs ≥65)	Registry diagnosis of osteoarthritis secondary to DDH	No (Crowe/Hartofilakidis not available in registry data)	None (comparison within DDH: single-taper vs dual-taper stems)	Mean 9.0 yrs (ST) vs 9.3 yrs (DT); up to 19 yrs	Cementless THA; comparison of single-taper (head–neck modularity only) vs titanium dual-taper (neck–stem modularity); mixed cups and bearings; head sizes ≤28 mm vs >28 mm; no cobalt-chrome necks included	Large registry stem-modularity analysis in DDH; DT stems showed higher long-term survivorship (93.9% vs 91.6% at 15 yrs) and lower aseptic loosening but higher implant breakage (0.6%); no

				(ST) and 79.0% (DT)							difference in dislocation revisions; higher failure risk in younger males and with head size ≤28 mm; no metallosis observed
Bus 2024 [94]	Netherlands; Dutch Arthroplasty Register (LROI)	National registry-based retrospective cohort study with competing-risk analysis	2007–2019	DDH cohort: 3,032 THAs; 75% female; all patients <55 (≤50 not isolated)	Mean age: 44 ± 8.9 years (DDH group)	Registry diagnosis of secondary OA due to DDH (surgeon-reported)	No (Crowe/Hartofilakidis not available in registry data)	Y; primary osteoarthritis THA and Legg–Calvé–Perthes disease	Up to 10 years; minimum 2 years; competing-risk cumulative incidence reported	Mixed fixation (uncemented most common); mixed approaches; mixed bearings (CoP most common); head sizes predominantly 32–36 mm; no data on DM or surgical reconstruction techniques	Large contemporary registry of <55-yr patients; excluded MoM and hemiarthroplasty; DDH not associated with increased 10-yr aseptic cup revision or overall revision vs OA (10-yr overall revision 6.0%); diagnosis not radiographically validated; severity and technical complexity not captured
Boyle 2012 [93]	New Zealand; New Zealand National Joint Registry	Registry-based retrospective comparative cohort study	1999–2008	DDH cohort: 1,205 THAs; 74% female; 69% ≤55 yrs (≤50 not isolated)	Mean age: 49.3 yrs (DDH) vs 67.6 yrs (OA)	Registry diagnosis of developmental dysplasia of the hip	No (Crowe/Hartofilakidis not available in registry data)	Y; primary osteoarthritis THA (n = 40,589)	6-month PROMs; revision and mortality tracked via registry (up to ~10 yrs)	Mixed cemented, hybrid, and cementless THA; uncemented fixation more common in DDH; mixed surgical approaches; head size, bearing, DM not reported	Largest early registry comparison of DDH vs OA; no difference in 6-mo Oxford Hip Score or revision rate after adjustment; DDH associated with longer operative time and more bone grafting; early (6-mo) mortality slightly higher in DDH after multivariate adjustment; severity and reconstruction technique not captured
Biant 2009 [28]	Australia; single tertiary academic center (Concord	Prospective consecutive case series	~1997–2000	17/22 patients female; mean age 45 (≤50	Mean 45 years (range 23–74)	Radiographic DDH with secondary osteoarthritis	Y; Crowe III–IV only	None	Mean 10 years (range up to 10+ years); no loss to f/u	Cementless THA; modular S-ROM femoral stem; true acetabulum reconstruction; frequent femoral-head autograft (75%); femoral shortening osteotomy in 21%; mixed bearings (MoP,	Classic Crowe III–IV modular stem series; no S-ROM stem revisions or loosening at 10 yrs; proximal osteolysis in Gruen zones 1/7 only; acetabular revisions for

	Repatriation General Hospital, Sydney)			majority)						MoM, zirconium-on-poly, ceramic-on-poly, rare CoC); head sizes 22–32 mm; no dual mobility	wear/loosening occurred, stems durable; HHS improved 37 → 81; demonstrates advantage of modular stem for anteversion control in severe DDH
Berninger 2019 [22]	Germany; single tertiary academic orthopedic center (BG Trauma Center Murnau)	Retrospective single-center cohort study	2010–2014	39/50 patients female; ≤50 not isolated (mean age ~59)	Mean 59 years (range 34–85)	Radiographic DDH with secondary osteoarthritis	Y; Crowe II–IV	None	Mean 38 ± 16 months (range 10–66)	Cementless THA; intentional far proximal/high hip center cup placement (≥55 mm above inter-teardrop line); porous-coated titanium cups (Duraloc or Allofit); polyethylene liners; head sizes 28–36 mm; posterior approach; **no** femoral shortening osteotomy or structural bone grafting	Functional outcomes study of extreme high hip center strategy; subgrouped by cup height (55–65, 65–75, >75 mm); HHS and LEFS good for ≤75 mm but worse gait/ADL >75 mm; no dislocations or nerve palsy; one early cup loosening; short- to mid-term outcomes only
Benad 2022 [117]	France; single tertiary academic center (CHRU de Lille)	Retrospective continuous case–control study	1990–2010	124 THAs total; shelf group 61 THAs (79% female), control 63 THAs (73% female); ≤50 not isolated	Mean age at THA: 47 ± 10 yrs (shelf) vs 49 ± 8 yrs (control); range 23–75	Radiographic acetabular dysplasia (VCE ≤15° or VCA ≤15°); secondary OA	No formal Crowe/Hartofilakidis; prior congenital dislocation excluded	Y; DDH THA without prior shelf acetabuloplasty	Mean 13 ± 6 yrs (range 5–25); no loss to f/u	Mixed THA and resurfacing; posterolateral approach; mixed bearings (MoM, MoP, CoP, CoC); occasional acetabular reinforcement ring; autograft used if <30% coverage; head sizes mixed	Prior femoral osteotomy and other acetabular procedures excluded; shelf removal required intra-op in 77%; 15-yr survivorship similar between groups (89% shelf vs 83% control); functional scores slightly better without prior shelf

Ashraf 2014 [96]	United States; single tertiary referral center (Mayo Clinic institutional registry + cost database)	Retrospective matched cohort study (economic analysis)	2000–2008	DDH cohort: 354 hips; 81% female; mean age 49 (≤50 common but not isolated)	Mean ± SD: 49 ± 13 yrs (DDH) vs 54 ± 13 yrs (OA controls)	Radiographic DDH confirmed by chart and radiograph review	Y; Crowe 0–IV	Y; age-, sex-, and year-matched primary osteoarthritis THA (n = 1,029)	Minimum 2 yrs for inclusion; outcomes focused on index hospitalization and 90-day costs	Mixed fixation and bearing types; specialized implants frequently used in DDH; detailed implant constructs not stratified; no dual mobility reported	Cost-focused study; DDH associated with ~USD 450 higher index hospitalization cost vs OA, driven by OR and implant costs; Crowe III–IV hips had significantly higher costs and longer LOS; no difference in 90-day complication rates vs OA; severity (Crowe grade) directly correlated with cost
Asai 2025 [118]	Japan; single tertiary academic center (Nagoya University Hospital)	Retrospective propensity-matched comparative cohort study	2000–2014	PAO group: 50 hips (95.7% female); age ≤50 not isolated	Mean age at THA: 56 yrs (range 40–77)	Radiographic DDH (secondary OA)	Y; Crowe I–III	Y; primary THA without prior osteotomy (1:1 matched by age, sex, Crowe type)	Mean 13.0 yrs (range 10–21) PAO vs 13.6 yrs (10–22) control	Posterolateral approach; cementless cups (Trident, Secur-Fit AD, X3 RimFit); mixed cementless/cemented stems (Secur-Fit, Exeter); occasional bone grafting (18% PAO); no navigation/robotics; no dual mobility	Focus on THA after prior PAO/RAO/ERAO; functional outcomes slightly inferior vs primary THA but 10-yr survivorship comparable (96% PAO vs 98% control); cups placed more superiorly in PAO without increased loosening; Crowe IV excluded; emphasizes long-term (>10 yr) outcomes after acetabular osteotomy

Abbreviations: f/u = follow-up; Y = Yes; N = No; NR= Not Reported; NA= Not Applicable

Note: Outcomes were reported as hip-based (per operated hip) or patient-based (per individual) according to the original study design. When not explicitly stated, outcomes were classified as hip-based if revisions, dislocations, or survivorship were reported per implant, and as patient-based if complications, pregnancy-related outcomes, or patient-reported outcome measures were reported per individual.

Supplementary Table S2. Instability/dislocation outcomes in women ≤50: DDH vs non-DDH (primary endpoint).

First author (Year)	Data source / Design	N (women ≤50) DDH / non-DDH	Instability definition (dislocation/recurrent/any event)	Time window(s) reported (e.g., 90d, 1y, 5y)	Surgical approach (if reported)	Construct notes (head size, DM use, liner type)	Instability rate: DDH (n, %)	Instability rate: non-DDH (n, %)	Effect estimate for DDH vs non-DDH (HR/OR/RR; adjusted?)	Key modifiers/predictors reported (modifiable)	Comments (bias/era issues; DDH severity)
Zhang 2024 [85]	China; single-center joint registry; retrospective 1:1 matched cohort (robotic vs manual THA in DDH)	N (women ≤50) DDH / non-DDH: DDH only (robotic 147 + manual 147); women 256/294; ≤50 not isolated; non-DDH = NA	Dislocation event; additionally reports repeated dislocation requiring revision (manual group) and immediate postoperative unstable dislocation requiring	NR (events reported over entire f/u; minimum f/u ≥6 months)	Posterolateral approach	No DM/head size/liner details; robotic uses MAKO + Trident cup; manual uses Combicup or Pinnacle; cementless cups with screws; stems per above	1/147 (0.7%) dislocation; Manual: 5/147 (3.4%) dislocation	NA	NA (no non-DDH comparator; no OR/HR reported for dislocation)	Robotic improved cup positioning into defined “safe zone” vs manual (cup angle precision); dislocations in manual group noted outside safe zone	DDH severity captured (Crowe I–IV); interim short f/u; treatment choice not randomized (patient chose robotics), though matched analysis used

			second operation (robotic group)								
Zeng 2017 [101]	China; single-center retrospective case series	52 DDH hips / NA (non-DDH not included)	Early postoperative dislocation event	Early postoperative period (specific window not prespecified)	Posterior approach	Cementless Pinnacle cup; S-ROM modular stem; ceramic-on-ceramic predominates; no dual mobility; head size not reported	2/52 hips (3.8%) early postoperative dislocation	NA	NA	Subtrochanteric transverse shortening osteotomy used in all cases; modular stem allowed correction of femoral anteversion	Crowe IV only; high surgical complexity; older implant era (mid-2000s); instability events did not require revision
Yacovelli 2021 [112]	United States; single-center retrospective matched cohort	49 DDH with prior pelvic osteotomy / 147 DDH without prior osteotomy	Postoperative hip dislocation (single or recurrent event)	Entire f/u; no prespecified time window	Predominantly anterior; minority lateral, anterolateral, or posterior	Mean head size ~32 mm; ceramic heads predominant; polyethylene liners most common; dual mobility uncommon (≤5%)	1/49 (2.0%)	4/147 (2.7%)	OR 0.8 (95% CI 0.0–6.1), unadjusted; P = 1.000	Prior pelvic osteotomy; altered acetabular anatomy; increased use of screws and bone grafting; cup version differences	Crowe III–IV excluded; instability rates low in both groups; modern implants but long enrollment era; findings most applicable to low-grade DDH
Wang 2017 [102]	China; single-center retrospective cohort	76 DDH hips / NA	Postoperative hip dislocation event	Early postoperative period; events captured during f/u (no	Posterolateral approach	Cementless Pinnacle cup with screw fixation; S-ROM modular stem; ceramic-on-ceramic or metal-on-	3/76 hips (3.9%)	NA	NA	Subtrochanteric transverse shortening osteotomy to limit limb lengthening; modular stem to adjust femoral anteversion;	Crowe IV only; older implant era; dislocations treated successfully with closed reduction; no recurrent instability or

				prespecified window)		polyethylene bearings; head sizes 28–36 mm; no dual mobility				anatomic cup placement in true acetabulum	instability-related revision
Wang 2012 [100]	China; single-center retrospective cohort	820 DDH hips / NA	Postoperative hip dislocation requiring reduction by a physician (subluxations excluded)	Entire f/u; assessed at 3 months, 6 months, 1 year, 2 years, and 5 years	Anterior (28.8%), posterior (65.4%), lateral (5.9%)	Cementless cups; mixed stem fixation; S-ROM modular stem subgroup; femoral head sizes 22, 28, and ≥32 mm; no dual mobility	24/820 (2.93%)	NA	NA	Femoral head size (≥32 mm significantly reduced dislocation risk); combined anteversion outside 30°–45° associated with instability; anterior dislocation predominated (69.6%)	Crowe IV showed numerically higher but not statistically significant dislocation rate; older implant era; instability patterns strongly related to component version rather than DDH severity alone
Vigdorchik 2020 [84]	United States; multicenter institutional databases; retrospective cohort	79 DDH hips / NA	Postoperative instability events assessed; no dislocations or instability events observed	Entire f/u; no prespecified time windows	Posterior approach	CT-based preoperative planning included head diameter and liner type; S-ROM stem; no dual mobility reported	0/79 (0%)	NA	NA	None reported	DDH severity reported; no comparator group; short f/u limits instability risk assessment; robotic cohort from early adoption era

Viamont-Guerra 2020 [106]	France and Brazil; single-surgeon retrospective cohort	29 DDH hips / NA	Postoperative hip dislocation event	Entire f/u; no prespecified time windows	Direct anterior approach (Hueter) using traction table	Cemented and uncemented cups depending on era; polyethylene liners; femoral head autograft augmentation common; no dual mobility	0/29 (0%)	NA	NA	Anatomic cup placement in true acetabulum; femoral head autograft use; selective subtrochanteric shortening osteotomy; traction-table exposure	Crowe III–IV only; very low instability likely influenced by approach and reconstruction strategy; long implant era with evolving materials
Ukaj 2025 [107]	Kosovo; prospective institutional cohort	22 DDH hips / NA	Postoperative hip dislocation event	Early postoperative period; outcomes assessed through minimum 6-month f/u	Posterior approach	Dual mobility acetabular cup used in all cases; uncemented components; subtrochanteric femoral shortening osteotomy performed in all hips; liner and head size ranges not reported	0/22 (0%)	NA	NA	Use of dual mobility acetabular component; restoration of anatomic hip center; femoral shortening osteotomy to reduce nerve tension	Crowe IV only; instability risk likely mitigated by universal DM use; very short f/u limits inference on late instability
Tu 2022 [103]	China; retrospective institutional cohort	12 DDH hips / NA	Postoperative hip dislocation event requiring manual	Early postoperative period; one event at 2 weeks postoperatively	Posterolateral approach	Uncemented acetabular components; structural bone grafting used when required; femoral shortening osteotomy common; no	1/12 hips (8.3%)	NA	NA	Accurate cup placement using 3D-printed guiding templates; femoral shortening osteotomy to reduce nerve traction and instability risk	Crowe IV only; instability resolved with closed reduction; no recurrent dislocation; small cohort limits generalizability

			reduction			dual mobility; head size and liner type not reported				
Thillemann 2008 [26]	Danish Hip Arthroplasty Registry; nationwide retrospective cohort	≤50 not isolated; 890 acetabular dysplasia hips vs 53,694 primary osteoarthritis hips	Revision due to dislocation (closed reductions without revision not captured)	Early 0–6 months and late 0.5–12 years	NR	Femoral head size recorded in registry since 1997; majority 28–30 mm; all early dislocation-related revisions in dysplasia occurred with 28 mm heads; no dual mobility	14/890 (1.6%) early revision due to dislocation; total dislocation-related revisions 18/890 (2.0%)	303/53,689 (0.6%) early revision due to dislocation; total 699/53,694 (1.3%)	Adjusted RR for revision due to dislocation in acetabular dysplasia within 6 months 2.8 (95% CI 1.6–4.9)	Femoral head size; component positioning; fixation type	Instability captured only if leading to revision; early-era implants; lack of granular anatomy or approach data; DDH severity not stratified
Solarino 2021 [126]	Italy; single-center retrospective cohort	100 DDH hips / NA	Postoperative hip dislocation event	Entire f/u; no prespecified time windows	Direct lateral approach	Cementless hemispherical cups; Wagner cone femoral stem; polyethylene liners; femoral head sizes predominantly 28 mm and 32 mm; no dual mobility	0/100 (0%)	NA	NA	Direct lateral approach; avoidance of Crowe IV hips; combined anteversion maintained <55°	Crowe I–III only; absence of instability likely influenced by approach and exclusion of high-grade DDH; long enrollment era with evolving implants
Siddiqi 2020 [91]	United States; NSQIP nationwide registry;	≤50 not isolated; 557 DDH vs 557	Not directly captured; only reoperation	30 days postoperative only	NR	Implant constructs not available in database	NR	NR	Not estimable for instability specifically	Longer operative time; higher transfusion rate in DDH cohort	Registry captures perioperative risk but not true instability or revision; inability to stratify DDH

	retrospective propensity-matched cohort	primary OA	events recorded								severity likely biases results toward null; best interpreted as short-term safety comparison rather than survivorship study
Shen 2020 [109]	China; single-center retrospective cohort	DDH only; 69 patients (85 hips); ~78% female	Dislocation requiring revision surgery	Entire f/u period (minimum 6 years; mean ~9 years)	Posterolateral (modified Kocher–Langenbeck) approach	No dual-mobility; ceramic-on-ceramic predominant; conventional polyethylene used in revision case	1/85 hips (1.2%)	NA	Not estimable	Hip center height; lateralization >10 mm; bearing choice (polyethylene associated with osteolysis revision)	Midterm outcomes only; instability likely underestimated due to revision-only definition; Crowe II–III only; pre–dual-mobility era; results may not reflect contemporary constructs
Sato 2022 [111]	Japan; single-center retrospective propensity-matched cohort	DDH only; 168 hips / NA	Postoperative hip dislocation event	Early postoperative period through 3-month CT evaluation	Modified Watson–Jones approach	Cementless Trident acetabular cup and Accolade II femoral stem; no dual mobility reported; liner type and head size not specified	0/168 (0%)	NA	NA	Improved cup placement accuracy with robotic arm assistance; standardized CT-based preoperative planning	Instability likely underestimated due to very short f/u; Crowe III–IV excluded; study evaluates technical accuracy rather than clinical instability risk
Rajan 2025 [92]	United States; TriNetX national EHR	DDH 18–50 years n = 2802; primary	Prosthetic dislocation or subluxa	90 days, 1 year, and 2 years	NR	Implant constructs not available; no head size,	Event counts not provided; adjusted OR for	NR	Adjusted OR for prosthetic dislocation/ subluxation	Age; DDH etiology; anemia; heterotopic ossification; no	Administrative coding may undercapture true instability; revision indication

	registry; retrospective propensity-matched cohort	OA comparator derived from matched cohort	tion identified via ICD-10 codes			liner, or dual-mobility data	dislocation DDH vs OA 1.18 at 90 days, 1.13 at 1 year, 1.19 at 2 years		at 2 years 1.19 (95% CI 1.08–1.32)	implant-level modifiers available	not always linked to dislocation; lack of DDH severity, approach, and implant construct limits mechanistic interpretation
Osawa 2019 [114]	Japan; retrospective CT-based simulation with matched primary OA controls	56 DDH hips after PAO / 56 primary OA hips (matched by age, sex, Crowe class)	Not assessed	NA	NA (simulation study)	Standardized virtual cup size based on acetabular width; no clinical implant constructs evaluated; no dual mobility	NA	NA	NA	Hip center elevation (+10 mm) improved anterior, posterior, and superior acetabular sector angles and medial wall thickness, particularly in Crowe II–III	Not a clinical instability study; revision and dislocation risk cannot be inferred; useful for mechanistic explanation of acetabular deficiency after PAO rather than outcome comparison
Osawa 2016 [113]	Japan; single-center retrospective matched cohort	52 DDH hips after PAO / 104 primary DDH hips without PAO	Postoperative hip dislocation (closed reduction; no revision)	Entire f/u period; no prespecified windows	Posterior approach	Cementless and cemented acetabular components; polyethylene and ceramic bearings; no dual mobility; alignment guidewire used to target 45° inclination and 20° anteversion	1/52 hips (1.9%)	1/104 hips (1.0%)	Not estimable; event rates too low	Socket anteversion; proportion of cups in Lewinnek safe zone; altered acetabular morphology after PAO	Instability captured clinically rather than revision-based; mid-2000s implant era; prior PAO alters acetabular anatomy and cup positioning; Crowe III–IV not represented

Ors 2022 [104]	Turkey; single-center retrospective cohort	127 DDH hips / NA	Postoperative hip dislocation (single or recurrent), including liner dislocation	Entire f/u period; no prespecified early/late windows	Posterolateral approach	Cementless Wagner cone femoral stem; Trilogy IT acetabular shell; ceramic-on-ceramic bearing; head sizes 28 mm and 32 mm; no dual mobility; transverse subtrochanteric shortening osteotomy with autograft augmentation	5/127 hips (3.9%) experienced dislocation	NA	NA	Femoral shortening osteotomy technique; restoration of anatomic hip center; femoral anteversion correction; head size selection	Instability reported clinically rather than registry-based; no non-DDH comparator; implant era predates widespread dual mobility use; all hips Crowe IV
Mu 2016 [105]	China; single-center retrospective cohort	71 DDH hips / NA	Postoperative hip dislocation; includes events treated with closed reduction and those requiring revision	Entire f/u period; no prespecified early vs late window	Posterolateral approach	Cementless threaded cup with conventional polyethylene liner; femoral head sizes 22 mm and 28 mm; no dual mobility; high rate of subtrochanteric shortening osteotomy	2/71 hips (2.8%)	NA	NA	Femoral head size; cup inclination; polyethylene wear rate; femoral shortening osteotomy to reduce nerve tension	Crowe IV only; instability partly secondary to polyethylene wear; implant era predates highly cross-linked polyethylene and dual mobility constructs; outcomes may overestimate modern instability and revision risk
Mortazavi	Iran; single-center	DDH 171 hips /	Any postoperative	Entire f/u period	Direct anterior	Neutral liners only; no dual mobility;	21/171 hips (12.0%)	Primary OA 4/197 hips (2.03%);	DDH vs primary OA OR	Crowe severity (II–IV vs I); intraoperative	Dislocation defined clinically rather than

2022 [18]	retrospective cohort	primary OA 197 hips (plus other etiologies 367 hips)	hip dislocation (clinical diagnosis; includes closed reductions)	(mean ~3.5 years)	approach	femoral head sizes ≤32 mm associated with higher dislocation; Wagner Cone and Wagner SL stems associated with instability		other etiologies 8/367 hips (2.17%)	6.75 (p<0.001); DDH vs other etiologies OR 6.28 (p<0.001); multivariable predictors included higher Crowe grade, intraoperative fracture, postoperative acetabular offset ≤16 mm	fracture; femoral head size <32 mm; failure of offset restoration; Wagner Cone stem use; cup position outside Lewinnek safe zone	revision-based; direct anterior approach uncommon in severe DDH in other regions; no dual-mobility constructs; DDH severity strongly correlated with instability risk
Minoda 2006 [115]	Japan; single-center retrospective matched cohort	10 DDH hips after prior Chiari pelvic osteotomy / 20 DDH hips without prior surgery	Postoperative hip dislocation; none observed	Entire f/u period (mean ~3 years); no prespecified early/late windows	Posterolateral approach	Cementless acetabular components with frequent bulk femoral-head autograft; bearing surface not specified; no dual mobility; cup placed anatomically when possible	0/10 hips (0%)	0/20 hips (0%)	Not estimable	Altered hip biomechanics after Chiari osteotomy (more vertical joint force vector); cup position and offset restoration discussed as biomechanically relevant	Not an instability-focused study; very small sample; implant era predates modern bearings and dual-mobility constructs; results not generalizable to contemporary THA

Matsushita 2022 [120]	Single-center retrospective cohort of primary THA for developmental dysplasia of the hip (DDH)	220 hips total (201 hips with ≥5-year f/u); DDH only; female-predominant (179 women); women ≤50 not separately stratified; no non-DDH comparator	Postoperative hip dislocation (single or late events); recurrent instability not separately defined	Early postoperative period and cumulative mid-to long-term f/u (mean 11.4 years; range 5–14.6 years)	Posterolateral approach with posterior soft-tissue repair (external rotators and capsule repaired)	Metal-on-polyethylene predominates; head sizes commonly 28 mm and 36 mm; no dual-mobility constructs; conventional polyethylene and highly cross-linked polyethylene both used; modular S-ROM-A stem	Early dislocation 2/220 hips (0.9%); late dislocation 1 additional hip during f/u; cumulative dislocation rate ≈1.4%	NA (DDH-only cohort)	NR (no comparator group)	Stem anteversion adjustment based on intraoperative stability testing; posterior soft-tissue repair emphasized; no statistically significant predictors modeled for instability	Highly selected DDH population; no non-DDH control; dislocation events rare; Crowe classification reported but not linked to instability risk; modern modular stem era may limit generalizability
Liu 2021[108]	China; single-center retrospective comparative cohort	DDH only; DAA 23 hips / PLA 50 hips	Any postoperative hip dislocation	Entire f/u period (mean ~2–3 years)	Direct anterior approach vs posterolateral approach	Cementless cups; ceramic-on-ceramic and ceramic-on-polyethylene bearings; femoral head sizes predominantly 28 mm; no dual-mobility liners;	DAA 0/23 hips (0%); PLA 3/50 hips (6.0%)	NA	NA; descriptive comparison by surgical approach	Surgical approach; posterior soft-tissue preservation; accurate acetabular anteversion; limb-length equalization strategy	Short-term outcomes only; instability events low and approach-dependent; Crowe III–IV exclusively; no modern dual-mobility constructs; results reflect high-volume specialist DAA practice

						extensive soft-tissue releases and frequent subtrochanteric shortening osteotomy					
Lari 2025 [127]	International multicenter retrospective cohort study (4 centers, 3 countries)	49 hips (37 patients) with DDH only; 29 women (78.4%); mean age 35 years (range 20–60); majority ≤50; no non-DDH comparator	Postoperative hip dislocation (clinical diagnosis)	Entire f/u period (mean 7.8 years; range 3–12 years)	Posterior 48.6%, lateral 40.5%, direct anterior 10.8%	Cementless implants in 94.6%; median head size 28 mm (range 22–36); ceramic-on-polyethylene 78.4%, metal-on-polyethylene 16.2%, dual mobility 5.4%; tapered stems 62%, conical stems 38%; subtrochanteric shortening osteotomy in 64.9% (mostly plate-augmented)	0/49 hips (0%)	NA	NA	Accurate restoration of anatomic hip center; limb-length restoration <3–4 cm; combined anteversion; use of dual mobility in selected high-risk cases; femoral stem selection (tapered vs conical)	Crowe IV only; instability rare despite small head sizes; heterogeneous implants and approaches; modern era but limited dual-mobility utilization
Kristoffersson 2021[121]	Sweden; single-center retrospective observational study	56 hips in 50 patients with DDH only; 27 women (54%);	NR; instability or dislocation outcomes not	NA	Posterior approach	Multiple cementless acetabular components (Pinnacle, Reflection, Trilogy, Continuum,	NR	NA	NA	Component templating accuracy; center of rotation restoration; acetabular cup inclination and anteversion;	Study not designed to evaluate instability; absence of dislocation data limits applicability to instability outcomes; DDH severity skewed

	evaluating digital templating accuracy in cementless THA for DDH	age range 23–68 years; women ≤50 not separately stratified; no non-DDH comparator	assessed			Trident PSL, others); femoral components predominantly Corail; head size, bearing surface, and dual-mobility use not reported; no instability-focused construct analysis				femoral stem alignment; leg length discrepancy	toward Crowe I; implant era predates widespread dual-mobility use
Kim 2023 [29]	Single-institution retrospective cohort study (2015–2020)	Women ≤50 not isolated; cohort mean age 54.4 years; DDH only population (no non-DDH comparator)	Postoperative hip dislocation (any event)	Up to 2 years post-THA	Posterolateral approach for all cases	Dual-mobility (DM) vs fixed-bearing (FB) acetabular cups; DM cups used vitamin E-infused highly cross-linked polyethylene liners; FB heads 28–36 mm (most dislocations with 32 mm); DM effective bearing size ~42 mm	FB group: 7/78 hips (9.0%) DM group: 0/94 hips (0%)	NA (DDH-only cohort)	NR (no non-DDH comparator)	Use of dual-mobility construct; increased effective head size; polyethylene liner type	All patients had DDH; Crowe I–IV included; short f/u (2 years); retrospective design; no adjustment modeling; instability entirely absent in DM group
Kawasaki 2017 [97]	Japan; single-center	50 DDH hips in 50	NR; study did not	NA	Direct anterior approac	Cementless acetabular component	NR	NA	NA	NA for instability; study focused on muscle cross-	Not an instability study; no clinical

	prospective observational cohort study using MRI outcomes after THA	patients; predominantly female (D1: 33/37 female; D2–3: 12/13 female); mean age ~66 years; women ≤50 not isolated; DDH only; no non-DDH comparator for instability	assess postoperative dislocation or instability events		h (DAA) for all cases	(G7, Biomet); femoral stem Taperloc Complete Microplasty; head size, liner type, and bearing surface not reported; no dual-mobility constructs described				sectional area and fatty atrophy on MRI	instability outcomes collected; Crowe I–III DDH included; findings limited to MRI-based muscle damage after DAA
Kaiser 2022 [88]	Switzerland; single-center retrospective consecutive case series	29 hips in 24 patients with DDH only; 17 women (71%); mean age 43 years	Postoperative hip dislocation (any event)	Entire f/u period (mean 35 months; range 12–137 months)	Direct anterior approach (DAA)	Cementless acetabular components (Versafit or Fitmore cups); bulk femoral head autograft with screw fixation; femoral head sizes predominantly	0/29 hips (0%)	NA	NR (DDH-only cohort)	Anatomic restoration of center of rotation via medialization and distalization; acetabular press-fit stability; accurate cup anteversion under fluoroscopy	Hartofilakidis grade A–B only; no high-grade dysplasia; small sample size; no comparator group; instability events absent despite predominant 28 mm head use

	(2006–2018)	(range 18–75); majority ≤50; no non-DDH comparator				28 mm (28/29 hips) with one dual-mobility cup used in an elderly patient; polyethylene liners; press-fit cups with occasional screw augmentation					
Ikezaki 2024 [116]	Single-institution retrospective cohort study with 1:1 propensity-matched control group	Age not restricted to ≤50; Shelf group 46 hips (40 patients, 87% female); matched non-DDH Crowe I control group 46 hips (39 females)	Postoperative hip dislocation (any event)	Entire f/u; minimum ≥1 year	Lateral or anterolateral approach for all cases	Mixed cemented, cementless, and hybrid THA constructs; no dual-mobility use reported; standard fixed-bearing cups; frequent use of autologous bone grafting (87% shelf group, 80% control)	0/46 hips (0%)	0/46 hips (0%)	Not estimable (no instability events in either group)	Cup placement at anatomic hip center; acetabular bone coverage; bone grafting technique	Crowe I DDH only; prior shelf acetabuloplasty cohort; instability events absent in both groups; limited power for instability outcomes; conversion THA era spans 1997–2020
Holzapfel 2012 [119]	Germany; retrospective matched	Study group (cranial socket): 40 hips	Postoperative hip dislocation	Early postoperative period and	Anterolateral approach for	Cementless cobalt-chromium acetabular components	Cranial socket group: 1/40 hips (2.5%)	NA (DDH-only cohort)	NA	Cup inclination and anteversion; acetabular coverage; ability to	Hartofilakidis type II only; small sample; instability rare; implant era predates dual-

	cohort study comparing two acetabular reconstruction techniques in DDH	in 37 patients; 28 women; mean age 53.1 years Control group (bulk autograft + hemispherical cup): 40 hips in 35 patients; 32 women; mean age 52.3 years Women ≤50 not separately stratified; DDH only (Hartofilakidis type II)	(clinical diagnosis)	entire f/u (minimum 24 months)	both groups	with UHMWPE liners; femoral head sizes 28, 32, or 36 mm; no dual-mobility constructs; cranial socket (oval press-fit) vs hemispherical press-fit cup with bulk femoral-head autograft	Control (bulk autograft) group: 1/40 hips (2.5%)			restore anatomic center of rotation	mobility and modern bearings
Gharanizadeh	Iran; multicent	Total 134 hips (134	Postoperative hip	90-day postoperative	Lateral approac	Mixed cemented, cementless,	1/47 hips (2.1%)	0/87 hips (0%)	NR for instability (study not	Femoral shortening osteotomy; shelf/impaction	Primary study focus was VTE,

2025 [99]	er retrospective comparative cohort study (two arthroplasty centers)	patients); high-riding DDH (Crowe III–IV) 47 hips (83% female); control group 87 hips (51% female); women ≤50 not explicitly stratified	dislocation requiring clinical evaluation	period (minimum f/u ≥3 months)	h for all cases	and hybrid THA constructs; fixed-bearing acetabular components; no dual-mobility constructs reported; acetabular shelf or impaction grafting common in DDH group; frequent use of femoral shortening osteotomy in Crowe IV hips			powered or designed for dislocation outcomes)	grafting; operative duration; postoperative weightbearing restrictions	not instability; instability events rare; Crowe III–IV DDH only in exposure group; short f/u; outcomes hip-based
Galea 2018 [98]	Single-center prospective consecutive cohort study (2000–2002)	Age ≤50 not isolated; total 104 patients (123 hips); 112 hips DDH, 11 hips primary OA (non-DDH)	Postoperative hip dislocation (any event)	Up to 13-year f/u	Posterolateral approach for all cases	Cementless Trilogy acetabular shell with Longevity highly cross-linked polyethylene liner; 26 mm ceramic femoral head; no dual-mobility constructs	0/112 hips (0%)	0/11 hips (0%)	NR (no instability events)	Hip center height; component positioning (inclination, anteversion); polyethylene wear (none associated with instability)	Crowe I–IV included; instability outcomes hip-based; early 2000s implant era with small head sizes; no dislocations observed limits comparative inference

Fukushi 2018 [110]	Japan; single-center retrospective comparative cohort study focused on abductor muscle recovery after THA	100 hips in 100 patients with DDH-related secondary osteoarthritis only; 86 women (86%); mean age 65.5 years (range 40–86); women ≤50 not isolated; no non-DDH comparator for instability	NR; postoperative dislocation or instability events were not assessed	NA	Posterolateral approach for all cases; piriformis preserved	Cementless THA in all hips (PerFix910 stem and AMS cup, Kyocera); femoral head size 26 mm in 8 hips and 32 mm in 92 hips; bearing surface not specified; no dual-mobility constructs reported	NR	NA	NA	Vertical hip center elevation (V-shift >15 mm); restoration of hip center; prior surgery for DDH; femoral offset management	Study objective was biomechanical (abductor moment recovery), not instability; Crowe I–III DDH included (no Crowe IV); instability outcomes not collected; early 2010s implant era without modern instability-mitigating constructs
Fahlbusch 2023 [66]	Germany; single-center retrospective cohort study of primary THA for	126 patients (121 hips analyzed); 91 women (83%); mean	Postoperative hip dislocation (any event)	Entire f/u; minimum 21 years (events captured over full	Posterior approach in lateral decubitus position	Cemented polyethylene acetabular cups (Endo-Modell Mark III) in most cases; cementless cups used with CFP short	9 dislocations / 121 hips (7%)	NA	NR (no non-DDH comparator)	Prior pelvic or femoral osteotomy; acetabuloplasty; Crowe severity (I/II vs III/IV); fixation method (cemented vs cementless)	DDH-only cohort; surgeries performed 1997–2000 (pre-modern bearings and instability-mitigating constructs); Crowe I–IV included;

	DDH (1997–2000)	age 51 ± 9.5 years (range 21–65); women ≤50 not isolated; DDH-only cohort; no non-DDH comparator		observation period)	for all cases	stem; femoral heads metal or ceramic (ceramic 72%, metal 28%); head sizes not consistently specified; no dual-mobility constructs					instability events hip-based; retrospective design with loss to f/u
Engesaeter 2012 [25]	Nordic Arthroplasty Register Association (NARA); population-based registry study pooling Denmark, Norway, and Sweden	DDH group: 12,068 THAs (72.5% female); OA comparator: 288,435 THAs (59.6% female); age ≤50 not isolated (mean age DDH 56 years;	Revision due to dislocation (dislocation recorded as reason for revision)	Early postoperative period (first 6 months) and cumulative f/u up to 14 years	NR in registry data	Fixation type reported (cemented, uncemented, hybrid, reverse hybrid); head size, bearing surface, liner type, and dual-mobility use not available in registry	Dislocation revisions: 138/12,068 THAs (1.1%) over total f/u	Dislocation revisions: 2,485/288,435 THAs (0.86%)	Adjusted RR for revision due to dislocation: 1.18 (95% CI 0.98–1.42); first 6 months RR 1.9 (95% CI 1.5–2.5)	Younger age; uncemented fixation; early postoperative period (<6 months)	Registry-based diagnosis only (DDH Y/no); DDH severity not stratified; instability captured only if leading to revision (non-revised dislocations not captured)

		OA 69 years)									
Di Martino 2021 [122]	Italy; Emilia-Romagna regional arthroplasty registry (RIPO); retrospective registry cohort (2000–2017)	5,761 cementless THAs for DDH; 72–83% female across cohorts; mean age ~58–63 years depending on stem group; women ≤50 not isolated; DDH-only cohort	Revision surgery due to dislocation (registry-recorded reason for revision)	Early postoperative period and cumulative f/u up to 17 years	NR (registry data)	Cementless acetabular cups; multiple stem designs (anatomic, tapered wedge/rectangular, tapered cone; modular and non-modular); bearing surfaces ceramic-on-ceramic most common; head size ≥36 mm in ~25% overall; dual-mobility not reported	Revision for dislocation ranged from 0.2–0.6% by stem cohort; overall dislocation revision approximately 0.3–0.4%	NA	NA (DDH-only study)	Stem design; modularity; fixation geometry; acetabular reconstruction quality	Registry captures only dislocations leading to revision; conservative dislocations not captured; DDH severity not recorded; implant era spans early 2000s to mid-2010s
DeNovio 2025 [124]	Single-institution retrospective cohort study using an institutional total	Women ≤50 not isolated; mean age 48 years (range 30–72); 83% women;	Postoperative hip dislocation (any event, including closed or open	Dislocations reported across entire f/u period; survivorship free of	Posterior approach in 26 hips; anterolateral approach in 2 hips	Cementless THA with subtrochanteric shortening osteotomy; porous-coated acetabular components with dome screws; highly	Dislocations occurred in 4 hips total; 20-year survivorship free of dislocation 85% (approximat	NA (DDH-only cohort)	NR (no non-DDH comparator)	Surgical technique (subtrochanteric shortening osteotomy to reduce limb lengthening); restoration of anatomic hip center; use of	Crowe IV DDH exclusively; historical implant era (1992–2005); no dual-mobility use; small sample size; hip-based outcome reporting; instability events

	joint registry (Mayo Clinic); surgeries performed 1992–2005 with long-term f/u	DDH-only cohort (Crowe IV); no non-DDH comparator	reduction)	dislocation reported to 20 years		cross-linked polyethylene liners; no dual-mobility constructs reported; femoral stems primarily extensively porous-coated	ely 15% cumulative dislocation risk)			highly cross-linked polyethylene	managed primarily nonoperatively
Colo 2016 [90]	The Netherlands; single-center retrospective cohort study of primary THA for DDH (1984–1995)	28 hips in 22 patients; 17 women (77%); mean age 48 years (range 26–74); 54% of hips in patients <50 years; DDH-only cohort; no non-DDH comparator	Postoperative hip dislocation (any event)	Entire f/u period (minimum 15 years; mean 20 years)	Not explicitly reported	Cemented acetabular cups with impaction bone grafting; Elite (DePuy) and Müller cups; cemented stems; polyethylene liners; femoral head size not reported; no dual-mobility constructs	0/28 hips (0%)	NA	NA (DDH-only cohort)	Acetabular reconstruction technique (impaction bone grafting); restoration of center of rotation; avoidance of excessive limb lengthening	All Crowe grades I–IV included; no dislocations observed despite severe dysplasia; historical implant era; instability outcomes hip-based; surgical approach and head size not reported

Clark 2025 [95]	Single-institution retrospective propensity-matched cohort study using an institutional joint replacement registry (2010–2022)	Age ≤50 not isolated; mean age DDH 51.7 ± 13.4 years, OA 54.0 ± 9.0 years; 38 dysplastic hips and 152 osteoarthritis hips; 68.4% female in DDH cohort	Postoperative hip dislocation requiring clinical documentation or revision (not separately reported as a primary endpoint)	Entire f/u period; mean f/u 5.2 ± 2.8 years (DDH) and 5.1 ± 2.8 years (OA)	Direct anterior approach for all cases	Conventional fixed-bearing constructs; mean femoral head size 35.2 ± 2.5 mm (DDH) vs 34.8 ± 2.6 mm (OA); polyethylene liners; no dual-mobility constructs reported	Not explicitly reported; no dislocations described in results	Not explicitly reported	NR	Surgical approach standardization; acetabular fixation with increased screw use in DDH; femoral shortening osteotomy and bone grafting in higher Crowe grades	Instability not a primary outcome; low observed event rate; predominance of Crowe I hips (86.8%); experienced tertiary referral surgeons; potential under-detection of minor instability events
Chen 2018 [23]	China; single-center prospective observational cohort study with healthy control	45 DDH patients (39 women, 6 men); mean age 51–55 years; women ≤50 not isolated; DDH-	NR; postoperative instability or dislocation events were not assessed	NA	Posterolateral approach for all THAs	Cementless acetabular and femoral components (DePuy); ceramic-on-ceramic bearing; femoral head size not specified; no	NR	NA	NA	Postoperative leg length discrepancy (<10 mm vs 10–20 mm); restoration of hip center; avoidance of residual limb-length inequality	Study focused on gait kinematics and compensatory mechanisms; instability outcomes not collected; Crowe II–III only; outcomes hip-based; no revision or adverse event reporting

	comparison	only surgical cohort; separate healthy control group without THA				dual-mobility constructs					
Chai 2022 [128]	China; single high-volume academic center (PLA General Hospital, Beijing); retrospective propensity score–matched comparative cohort study (1:1), robotic-assisted vs conventi	DDH only; matched cohort of 54 hips (27 robotic-assisted, 27 conventional); 100% female in matched groups; mean age 43.0 ± 8.9 years (robotic) vs 44.6 ± 9.5 years	Postoperative hip dislocation reported as a postoperative complication		Posterolateral approach	Cementless THA; robotic-assisted Mako vs conventional/ manual THA; Trident cups in robotic group vs Pinnacle/Combicup in conventional group; modular S-ROM femoral stem; true acetabulum reconstruction when possible; frequent subtrochanteric shortening osteotomy; head size NR;	0/54 hips (0%); robotic 0/27 and conventional 0/27	NA	NA; no non-DDH comparator and no dislocation events	Robotic assistance/component-positioning accuracy; significantly greater Lewinnek/Callanan safe-zone cup placement with robotic-assisted THA; no specific instability predictor identified	Crowe III–IV DDH only; no dislocations reported in either group; comparison was robotic-assisted vs conventional THA within DDH rather than DDH vs non-DDH; short-term study focused primarily on acetabular cup-positioning accuracy rather than instability or survivorship

	onal THA	(conventional); women ≤50 not separately isolated; non-DDH = NA				no dual mobility					
Caylak 2021 [125]	Single-institution retrospective cohort study (2008–2011)	50 patients (43 women, 7 men) / 67 hips; DDH-only population (Crowe type IV); no non-DDH comparator; mean age 41.5 years (range 23–61)	Postoperative hip dislocation (any event requiring reduction)	Up to minimum 10 years post-THA (mean f/u 131.9 months)	Posterolateral approach for all cases	Cementless ceramic-on-ceramic (CoC) bearings; femoral head sizes 22 mm (2 hips), 28 mm (48 hips), 32 mm (17 hips); no dual-mobility constructs used	4/67 hips (6.0%)	NA (DDH-only cohort)	NR (no non-DDH comparator)	Femoral head size; component positioning; soft-tissue tensioning after subtrochanteric shortening osteotomy	All hips Crowe type IV DDH; complex reconstructions with transverse subtrochanteric shortening osteotomy; dislocations managed with closed reduction (2 hips) and open reduction (2 hips); retrospective design; no multivariable adjustment; instability outcomes hip-based rather than patient-based

Castagnini 2022 [123]	Italy; Emilia-Romagna Arthroplasty Registry (RIPO); population-based retrospective registry cohort (2000–2018)	6,429 cementless THAs performed for osteoarthritis secondary to DDH; 73.7% women in single-taper (ST) cohort and 79.0% women in dual-taper (DT) cohort; mean age 61.0 years (ST) and 58.6 years (DT); women ≤50 not isolated;	Revision due to recurrent dislocation or primary instability (registry-recorded reason for revision)	Entire f/u period; mean f/u 9.0 years (ST) and 9.3 years (DT); maximum f/u 19 years	Reported in registry but not analyzed or stratified in results	Single-taper vs titanium-on-titanium dual-taper femoral stems; bearing surfaces included ceramic-on-ceramic, ceramic-on-polyethylene, metal-on-polyethylene, and metal-on-metal; head sizes ≤28 mm and >28 mm; no dual-mobility constructs included	Recurrent dislocation revisions: 0.7% in ST cohort and 0.6% in DT cohort	NA	NA (DDH-only cohort)	Head size ≤28 mm; male sex; younger age; stem design (specific dual-taper models associated with higher instability)	Instability captured only if revised; conservatively treated dislocations not recorded; DDH severity not available; implant era predates widespread dual-mobility use; outcomes hip-based rather than patient-based

		DDH-only cohort									
Bus 2024 [94]	Dutch Arthroplasty Register (LROI); nationwide population-based retrospective registry cohort (2007–2019)	Patients <55 years included; DDH group n=3,032 THAs (25% male → ~75% female); non-DDH comparator = primary osteoarthritis (OA) n=20,645 THAs; women ≤50 not isolated	Revision for instability not analyzed as a separate endpoint; instability captured only if leading to revision for any reason	2-, 5-, and 10-year cumulative incidence	Multiple approaches reported (posterolateral most common); not analyzed as a predictor	Mixed fixation and bearing types; predominantly uncemented cups; femoral head sizes 22–28 mm, 32 mm, and 36 mm; no reporting of dual-mobility constructs as a distinct category	Not separately reported (only all-cause revision and aseptic cup revision available)	Not separately reported	NR for instability specifically	None specific to instability; models adjusted for age, sex, BMI, cup size, and previous surgery	Registry captures only revised instability; non-revised dislocations not included; DDH severity not available; outcomes hip-based rather than patient-based
Boyle 2012 [93]	New Zealand National Joint	DDH: 1,205 primary THAs	Revision for dislocation	Entire f/u period; early	Posterolateral, anterolateral,	Fixation method reported (cemented,	Dislocation revisions occurred but exact	Dislocation revisions occurred but exact	Adjusted HR for revision due to any	Operative approach; cementation type;	Instability captured only if revised; non-revised

	Registry; nationwide population-based registry cohort study (1999–2008)	(74% women; mean age 49.3 years); non-DDH comparator OA: 40,589 primary THAs (52% women; mean age 67.6 years); women ≤50 not isolated	recorded as reason for revision in registry	postoperative period analyzed separately; revision rates reported per 100 component-years	and anterior approaches recorded; approach included in multivariable models	uncemented, hybrid); bearing surface, head size, liner type, and dual-mobility use not recorded in registry	number not isolated; included within 42 total revisions (revision rate 0.79 per 100 component-years)	number not isolated; included within 1,031 total revisions (revision rate 0.61 per 100 component-years)	cause: 1.08 (95% CI 0.75–1.55); no significant difference for dislocation-specific revision	operative time; patient age and sex	dislocations not recorded; DDH severity not reported; registry diagnosis not radiographically validated; outcomes hip-based
Biant 2009 [28]	Australia; single-center prospective cohort study of primary THA for severe DDH	22 patients (28 hips); 17 women (77%); mean age 45 years (range 23–74); majority ≤50;	Postoperative hip dislocation (any event)	Entire f/u period with serial assessments to 10 years	Posterolateral approach with patient in lateral position	Cementless modular S-ROM femoral stem; cementless acetabular components (multiple DePuy designs); polyethylene liners; femoral head sizes 22 mm (1 hip), 26	0/28 hips (0%)	NA	NA	Femoral anteversion control via modular stem; acetabular placement at true hip center; use of femoral shortening osteotomy to avoid excessive lengthening	Severe DDH only; zero dislocations despite small head sizes; historical implant era; instability outcomes hip-based; technically demanding surgeries performed by experienced surgeon

		DDH-only cohort (Crowe III–IV); no non-DDH comparator				mm (3 hips), 28 mm (22 hips), 32 mm (2 hips); no dual-mobility constructs; frequent autologous femoral-head bone grafting; subtrochanteric shortening in 21%					
Berninger 2019 [22]	Germany; single-center retrospective cohort study of primary THA for severe dysplastic hip osteoarthritis with far proximal cup positioning	50 patients (61 hips); 39 women (78%); mean age 59 years (range 34–85); women ≤50 not isolated; DDH-only cohort (Crowe II–IV); no non-	Postoperative hip dislocation (any event)	Entire f/u period; minimum 10 months, mean f/u 38 ± 16 months	Posterior (Moore) approach in all cases	Cementless titanium porous-coated acetabular cups (Duraloc or Allofit); polyethylene liners (Marathon or Durasul); femoral head sizes predominantly 28–36 mm (mean ~30–32 mm); no dual-mobility constructs; occasional	0/61 hips (0%)	NA	NA	Cup height relative to interteardrop line; acetabular inclination (40–50° target); femoral head size; avoidance of femoral shortening osteotomy	DDH-only cohort; Crowe II–IV included; far proximal cup positioning up to >75 mm; instability outcomes hip-based; short-to-midterm f/u; single-center surgical expertise may limit generalizability

	(2010–2014)	DDH comparator				screw fixation of cups					
Benad 2022 [117]	Single-center retrospective case–control cohort study of THA for acetabular dysplasia, comparing hips with prior shelf acetabuloplasty versus dysplasia without prior surgery (procedures performed 1990–2010)	Women ≤50 not isolated; mean age at THA 47 ± 10 years (shelf group) and 49 ± 8 years (control); all hips had DDH; comparator group was DDH without prior shelf acetabuloplasty	Postoperative hip dislocation or chronic instability requiring revision	Entire f/u period; minimum 5 years, maximum 25 years	Posterolateral approach for all cases	Mixed implant era; no dual-mobility constructs reported; bearings included metal-on-metal, metal-on-polyethylene, ceramic-on-polyethylene, and ceramic-on-ceramic; femoral head sizes not consistently reported	Shelf group: 1 case of chronic posterior instability requiring revision (1/61 hips, ~1.6%)	Control group: 0 revisions for instability; dislocations reported but not leading to revision	NR; no adjusted or unadjusted comparative risk estimate provided	Prior shelf acetabuloplasty; acetabular reconstruction techniques; implant positioning; impingement management	All patients had DDH; instability events were rare; outcomes reflect older implant era; retrospective design; no multivariable adjustment; instability reported at hip-level rather than patient-level

Ashraf 2014 [96]	United States; single-institution matched retrospective cohort study using an institutional total joint registry and longitudinal administrative database (2000–2008)	354 patients with DDH undergoing primary THA matched to 1,029 patients with primary osteoarthritis; DDH cohort 81% women, mean age 49 years (SD 13); women ≤50 not isolated	NR; postoperative dislocation or instability events were not specifically assessed	Perioperative hospitalization and 90-day postoperative period only	NR	Implant type and bearing surfaces not detailed; specialized implants commonly used in DDH; no reporting of femoral head size or dual-mobility constructs	NR	NR	NA	DDH severity by Crowe classification; implant cost; operative time; length of stay	Study focused on hospital cost and early complications; instability outcomes not captured; DDH severity assessed using Crowe classification (0–IV); outcomes hip-based; short-term window only
Asai 2025 [118]	Japan; single-center retrospective propensity-	PAO group: 46 patients (50 hips), 95.7%	Postoperative hip dislocation (any event)	Entire f/u period with minimu	Posterolateral approach in lateral decubitus	Cementless acetabular cups (Trident, Secur-Fit AD, X3 RimFit); Secur-Fit or Exeter stems;	PAO group: 2/50 hips (4.0%)	Control group: 1/50 hips (2.0%)	NR; no adjusted or unadjusted comparative risk	Cup position (vertical and horizontal hip center); bone grafting for acetabular defects; prior osteotomy	Instability events rare in both groups; outcomes hip-based; no multivariable modeling for instability; DDH

	matched cohort study comparing THA after periacetabular osteotomy (PAO/RAO/ERAO) versus primary THA (January 2000–June 2014)	women; control group: 48 patients (50 hips), 91.7% women; mean age at THA 56 years in both groups; women ≤50 not isolated; DDH population only		m 10-year f/u	position for all cases	polyethylene liners (X3); head size not explicitly reported; no dual-mobility constructs; bone grafting used in 18% of PAO hips			estimate provided	anatomy; implant positioning	severity balanced via propensity matching

Abbreviations: f/u = follow-up; Y = Yes; N = No; NR= Not Reported; NA= Not Applicable

Note: Instability hierarchy for synthesis was defined as follows: dislocation event → recurrent instability → revision for instability. Dual-mobility (DM) and constrained constructs may reflect confounding by indication, as these implants are often used in higher-risk hips.

Supplementary Table S3. Revision outcomes and revision mechanisms in women ≤50 undergoing THA: DDH vs non-DDH.

**First author (Year)**	**Country / Data source**	**Design**	**N (women ≤50) DDH /**	**f/u (mean /median; range)**	**Survival metric / reporting (KM,**	**Timepoint(s) reported (1/5/10/15/20y)**	**All-cause revision DDH**	**All-cause revision non-DDH (n, %)**	**Effect estimate for DDH vs non-DDH (HR/O**	**Instability-related revision DDH (n, %)**	**Instability-related revision non-DDH (n, %)**	**Other major causes of revision reported (list + DDH vs non-**	**DDH severity reported? (Y/N; system)**	**Implant era / key constructs (bearing, head size, DM use)**	**Notes (definition of revision; overlap; key limitations)**

			non-DDH		competing risk, crude)		(n, %)		R/RR; adjusted?)			DDH if available)			
Zhang 2024 [85]	China; prospectively constructed single-center joint registry (PLA General Hospital system)	Retrospective propensity score–matched cohort (robotic-assisted vs manual THA in DDH)	256 (87%); ≤50 not explicitly stratified	Minimum f/u ≥6 months; mean/median/range not reported	NR	None prespecified; events reported over entire f/u	Manual 2/147 (1.4%); Robotic 1/147 (0.7%)	NA (DDH-only study)	NA	Manual 2/147 (1.4%) recurrent dislocation; Robotic 1/147 (0.7%) early unstable dislocation requiring head exchange	NA	Periprosthetic joint infection; aseptic loosening; periprosthetic femoral fracture; unexplained hip pain (no DDH vs non-DDH comparison)	Y; Crowe I–IV	2018–2022; posterolateral approach; cementless acetabular components (Trident for robotic, Pinnacle/Combi cup for manual); S-ROM modular stem for excessive femoral anteversion; head size, bearing surface, and dual mobility use not reported	DDH-only cohort; short-term f/u; revision defined as reoperation with component exchange; nonrandomized treatment allocation despite propensity matching

Zeng 2017 [101]	China; single-center academic hospital (Third Military Medical University, Chongqing)	Retrospective single-center case series	37 women / 45 patients (82% female); mean age 40.6 years (range 18–62); ≤50 not explicitly isolated	Mean 9.8 years (range 8.9–11.8 years)	Crude survivorship; no Kaplan–Meier or competing-risk analysis	1 year, 5 years, and 10 years	0/52 hips (0%)	NA (DDH-only study)	NA	0/52 hips (0%)	NA	None; no component loosening, migration, or nonunion observed	Y; Crowe type IV only	2005–2008; posterolateral approach; cementless Pinnacle acetabular component; S-ROM modular femoral stem; ceramic-on-ceramic bearing in 49 hips, ceramic-on-polyethylene in 3 hips; dual mobility not used; head size not reported	Revision defined as component exchange; no revisions observed; DDH-only high-grade cohort; no comparator group; relatively small sample size; survivorship inferred from absence of revision rather than formal survival analysis
Yacovelli 2021 [112]	United States; single high-volume acade	Retrospective matched cohort study (1:3	49 DDH with prior pelvic osteotomy (43 wome	Mean 3.8 years (±2.6 prior PO; ±2.1 controls);	Crude revision rates; no Kaplan–Meier or	None prespecified; events reported over entire f/u	6/49 (12.2%)	6/147 (4.1%)	OR 3.3 (95% CI 1.0–11.2), unadjusted; P = 0.077	1/49 (2.0%)	4/147 (2.7%)	DDH: aseptic loosening (2), periprosthetic joint infection (1), periprosthetic fracture	Y; Crowe I–II only (Crowe III–IV excluded)	2000–2018; predominantly anterior approach; cementless acetabular components; mean head size ~32 mm;	Revision defined as component exchange; reoperation broader than revision; relatively short f/u;

	mic center arthroplasty database	propensity-matched)	n, 87.8%); 147 DDH without prior osteotomy (125 women, 85.0%); ≤50 not explicitly stratified (mean age ~40)	range not reported	competing-risk analysis							(1), cortical perforation (1), instability (1)<br>Non-DDH: aseptic loosening (1), instability (4), ceramic head fracture (1)		ceramic heads predominant; dual mobility rare (2.0% DDH vs 4.8% non-DDH)	DDH limited to low-grade dysplasia; prior pelvic osteotomy alters anatomy and surgical complexity
Wang 2017 [102]	China; West China Hospital institutional arthroplasty	Retrospective single-center cohort	62 patients (76 hips); 49 women (79%); mean age	Mean 10 years (range 6.6–13.2 years)	Kaplan–Meier survivorship analysis	10-year survivorship	2/76 hips (2.6%)	NA (DDH-only study)	NA	0/76 hips (0%)	NA	Aseptic loosening of femoral stem (1); aseptic loosening of acetabular component following	Y; Crowe type IV / Hartofilakidis type III	2002–2010; posterolateral approach; cementless Pinnacle acetabular cup with screw fixation; S-ROM modular femoral stem;	Revision defined as component exchange; no control group; high surgical complexity; single-center experience;

	registry **Design:** Retrospective single-center cohort study	rt study	46.5 years (range 19–73); ≤50 not isolated								acetabular fracture (1)		ceramic-on-ceramic bearing in 60 hips and metal-on-polyethylene in 16 hips; head sizes 28 mm (34 hips), 32 mm (30 hips), 36 mm (12 hips); dual mobility not used	outcomes specific to high-grade DDH treated with subtrochanteric shortening osteotomy
Wang 2012 [100]	China; single-center institutional total joint registry (Shanghai Ninth People's	Retrospective cohort study	611 women / 820 patients (74.5% female); mean age 49.8 years (range 12–86); ≤50	Mean 3 years; minimum 6 months	No formal survivorship analysis (no Kaplan–Meier or competing-risk curves); crude	f/u visits at 3 months, 6 months, 1 year, 2 years, and 5 years	7/820 (0.85%) required revision for recurrent dislocation	NA (DDH-only study)	NA	7/820 (0.85%) revision for recurrent dislocation	NA	Revision only reported for recurrent dislocation; aseptic loosening, infection, and fracture revisions not quantified	Y; Crowe I–IV	2000–2009; anterior, posterior, and lateral approaches; cementless acetabular components; cemented stems in 94 hips, cementless stems in remainder; S-ROM modular stem in 106 hips (0 dislocations); femoral head	Dislocation defined as event requiring physician reduction; revision performed only for recurrent instability; large heterogeneous surgeon cohort; short-to-midterm f/u; outcomes

	Hospital)		not isolated		event rates reported										sizes 22 mm, 28 mm, and ≥32 mm (larger heads associated with lower dislocation risk); dual mobility not reported	limited to instability rather than comprehensive revision etiologies
Vigdorchik 2020 [84]	United States; two high-volume academic institutions, institutional arthroplasty databases	Retrospective multicenter cohort study	56 women of 79 total patients (71% female); mean age 45 years (range 26–64); ≤50 not explicitly stratified	Mean 3.1 years (range 2.0–4.6 years)	NR; no Kaplan–Meier or competing-risk analysis	None prespecified; outcomes reported at latest f/u	0/79 (0%)	NA (DDH-only study)	NA	0/79 (0%)	NA	None; no revisions or reoperations reported	Y; Crowe I–IV and Hartofilakidis A–C	2010–2015; posterior approach; CT-based robotic-assisted THA (MAKO); S-ROM modular femoral stem; head size and liner type planned preoperatively but final implanted sizes not reported; dual mobility not reported	DDH-only cohort; short-term f/u; no comparator group; discrepancy in reported study years between abstract and methods

Viamont-Guerra 2020 [106]	France and Brazil; single high-volume surgeon series at Ramsay Santé (Paris) and Hospital Israelita Albert Einstein (São Paulo)	Retrospective single-surgeon cohort study	23 patients (29 hips); 22 women (76%); mean age 49.1 years (range 17–75); ≤50 not isolated	Mean 8.4 years (±4.7); range 2–20 years	No formal survival analysis; crude revision counts reported	Outcomes reported over entire f/u; revisions occurred at 14, 16, and 18 years	3/29 hips (10.3%)	NA (DDH-only study)	NA	0/29 (0%)	NA	Polyethylene wear with loosening (3 hips); no infection- or instability-related revisions	Y; Crowe III (69%) and Crowe IV (31%)	1993–2016; direct anterior approach on traction table; cups placed in true acetabulum; femoral head autograft used in 90%; subtrochanteric shortening osteotomy in 17%; uncemented cups in 76%, hybrid or cemented remainder; polyethylene liners (non-cross-linked in early era); dual mobility not used	Revision defined as component exchange; no comparator group; heterogeneous implants across long era; survivorship analysis not performed due to small cohort and variable f/u

Ukaj 2025 [107]	Kosovo; University Clinical Center of Kosovo and Otrila Hospital	Prospective single-center cohort study	19 women of 22 total patients (86.3% female); mean age 49.2 years (SD ±12.1); ≤50 not explicitly stratified	Minimum 6 months; f/u assessments at 1, 3, and 6 months; no long-term f/u reported	NR; crude complication and revision reporting only	None; short-term f/u only	0/22 (0%)	NA (DDH-only study)	NA	0/22 (0%)	NA	None; no revisions reported; osteotomy union achieved in all cases	Y; Crowe type IV only	2016–2022; posterior approach; uncemented dual mobility acetabular cup (Quattro); uncemented femoral stem with subtrochanteric shortening osteotomy; dual mobility used in all cases; head size and bearing materials not individually specified	Short-term outcomes only; no comparator group; revision defined as component exchange; limited sample size; survivorship conclusions limited by 6-month f/u
Tu 2022 [103]	China; multi-institutional academic hospit	Retrospective single-arm coho	8 women of 12 total patients (66.7	Mean 72.4 months (range 38–135	No Kaplan–Meier or competing-risk	Final f/u at mean ~6 years; no fixed prespecified intervals	0/12 hips (0%)	NA (DDH-only study)	NA	0/12 hips (0%)	NA	None; no component loosening, migration, or infection reported	Y; Crowe type IV only	2007–2015; posterolateral approach; acetabular cup placed in true acetabulum using 3D-printed	Revision defined as component exchange; DDH-only cohort; no control group; small

	als (Southern Theatre Command of PLA–affiliated centers)	rt study	% female); mean age 33.3 years (range 20–46); all ≤50	months)	analysis; crude survivorship reported									individualized guiding template; uncemented acetabular components with screw fixation; femoral shortening osteotomy in 7 hips; femoral anteversion corrected to ~10°; bearing surface, head size, and dual mobility use not reported	sample size; long and variable f/u; results reflect technical feasibility rather than comparative effectiveness
Thillemann 2008 [26]	Denmark; Danish Hip Arthroplasty Registry linked with National	Nationwide population-based retrospective coho	≤50 subgroup not isolated; total cohort 56,087 THAs; acetabular dysplasia	Mean 4.6 years; range 1 day to 12 years	Kaplan–Meier survivorship curves with Cox proportional hazards	Early 0–6 months and late 0.5–12 years; 10-year cumulative failure rates reported	Acetabular dysplasia 43/890 (4.8%) overall; 19 revis	Primary osteoarthritis 2,204/53,694 (4.1%) overall	Adjusted RR for revision in acetabular dysplasia 0–6 months 1.9 (95% CI 1.2–	14/890 (1.6%) within first 6 months; additional 4/802 (0.5%) after 6 months	303/53,689 (0.6%) within first 6 months; 396/49,791 (0.8%) after 6 months	Aseptic loosening (acetabular or femoral), deep infection, femoral fracture, implant component failure, pain; dislocation most	N; DDH defined by registry diagnosis (acetabular dysplasia), no Crowe or Hartofilakidis stratification	1995–2006; cemented, uncemented, and hybrid fixation; femoral head sizes predominantly 28–30 mm; >30 mm used more frequently in dysplasia; dual	Revision defined as partial or complete component removal or exchange; reductions without exchange excluded; registry-based

	Patient Registry and Civil Registration System	rt study	subgroup 890 hips (742 patients); 611 women with dysplasia		regression (adjusted relative risks)		ions in first 6 months, 24 revisions from 6 months to 12 years		3.1); no increased adjusted risk beyond 6 months			common early cause, especially in acetabular dysplasia		mobility not reported	diagnosis may be misclassified; no patient-level anatomy or surgical approach data
Solarino 2021 [126]	Italy; single-center academic hospital arthroplasty database	Retrospective single-center cohort study (Level IV case	63 women of 87 patients total (72% female); mean age 53 years (range 27–88);	Mean 11.7 years (range 2.2–21.8 years)	Kaplan–Meier survivorship analysis	5-year, 10-year, and 20-year survivorship reported	1/100 hips (1.0%) revision for suspected acetabular loos	NA (DDH-only study)	NA	0/100 (0%)	NA	No revisions for aseptic loosening of stem or cup, infection, periprosthetic fracture, or instability	Y; Crowe I–III (no Crowe IV hips)	1993–2016; direct lateral approach; cementless hemispherical acetabular components; Wagner cone uncemented femoral stem (125° or 135° neck); bearing surfaces ceramic-on-polyethylene,	Revision defined as component exchange; long and heterogeneous implant era; no comparator group; DDH limited to Crowe I–III; survivorship reflects stem-focused

		series)	≤50 not isolated				opening with liner and head exchange						metal-on-polyethylene, or ceramic-on-ceramic; femoral head sizes 28 mm, 32 mm, and 36 mm; dual mobility not used	outcomes rather than global THA revision risk
Siddiqi 2020 [91]	United States; American College of Surgeons National Surgical Quality Improvement Program	Nationwide retrospective propensity-matched cohort study	≤50 not isolated; matched cohorts included 557 DDH patients and 557 primary OA patients; DDH cohort 72.7%	30-day postoperative f/u only (NSQIP limitation)	NR; no Kaplan–Meier or competing-risk analysis	30 days only	NR beyond 30 days; reoperation within 30 days 13/557 (2.4%)	NR beyond 30 days; reoperation within 30 days 9/557 (1.7%)	Adjusted OR for reoperation within 30 days 1.43 (95% CI 0.61–3.40), not statistically significant	NR; NSQIP does not capture indication for reoperation	NR	Not specified; NSQIP captures reoperation but not revision indication or implant failure mechanism	N; DDH identified by ICD-9/ICD-10 codes without severity stratification	2008–2016; implant type, head size, bearing surface, and dual mobility use not available in NSQIP	Outcomes limited to 30-day reoperation; true revision and long-term failure not captured; DDH severity and prior osteotomy not available; results likely underestimate risk in severe DDH

	(NSQIP) database		women, mean age 51.2 years												
Shen 2020 [109]	China; single tertiary academic center (PLA General Hospital, Beijing)	Retrospective single-center cohort study	Not explicitly stratified by age ≤50; mean age 46.5 ± 12.6 (Group A) and 46.4 ± 11.0 (Group B); 81% women overall	Mean 8.9 ± 1.8 years (range 6.0–14.1 years)	Kaplan–Meier survivorship analysis	Final f/u only (midterm); survivorship reported at ~9 years	2/85 hips (2.4%) overall DDH cohort	NA (DDH-only cohort)	NA (no non-DDH comparator)	1/85 hips (1.2%); revision for dislocation at 8.3 years	NA	Osteolysis (1 hip, 1.2%); occurred in metal-on-polyethylene construct	Y; Crowe classification (Crowe II and III only)	2003–2013; cementless cups; predominantly ceramic-on-ceramic bearings (~92%); no dual-mobility constructs reported	Revision defined as reoperation for any reason; DDH-only cohort limits comparative inference; no Crowe IV hips; single-surgeon series; limited generalizability to modern DM-era constructs

Sato 2022 [111]	Japan; single-center academic hospital (Nissan Tamagawa Hospital, Tokyo)	Retrospective single-center propensity score–matched cohort study (robotic-assisted vs manual THA)	≤50 not isolated; matched cohort 168 patients total (168 hips); 166 women (98.8%); mean age 66 years (SD ±8)	Short-term; postoperative CT evaluation at 3 months; no long-term f/u reported	NR; no Kaplan–Meier or competing-risk analysis	None; perioperative and 3-month outcomes only	0/168 (0%)	NA (DDH-only study)	NA	0/168 (0%)	NA	None; no infection, fracture, or dislocation reported during short-term f/u	Y; Crowe classification (Crowe I–II only; no Crowe III–IV)	2019–2020; modified Watson–Jones approach in lateral decubitus position; cementless Accolade II femoral stem and Trident acetabular cup (Stryker); press-fit cup with routine screw fixation; bearing surface, head size, and dual mobility use not reported	Study focused on accuracy of cup placement rather than clinical failure; extremely short f/u limits revision and instability assessment; DDH limited to low-grade dysplasia; not designed to assess survivorship
Rajan 2025 [92]	United States; TriNetX	Retrospective propensity	DDH 18–50 subgroup n = 2802; female	Up to 2 years postoperatively (90 days, 1	NR; no Kaplan–Meier or	90 days, 1 year, and 2 years	Revision odds reported; crud	NR separately (registry-based	Adjusted OR for revision DDH vs primary	NR as discrete revision counts; dislocation/subluxat	NR	Aseptic loosening, periprosthetic fracture, periprosthetic joint	N; DDH identified by ICD-10 diagnosis codes only	2014–2023; implant type, head size, bearing surface, and dual-mobility use not	Revision defined by CPT/ICD reoperation codes; no implant-level

	national federated electronic health record research network	score–matched cohort study	58.9%; non-DDH ≤50 subgroup size not isolated	year, 2 years)	competing-risk analysis		e counts not provided; adjusted OR for revision at 2 years 1.10 (95% CI 0.92–1.31)	odds modeling only)	OA at 2 years 1.10 (95% CI 0.92–1.31), not statistically significant	ion captured as complication		infection, heterotopic ossification; reported as odds ratios, not event counts		available in TriNetX	data; no radiographic severity; limited to short-term outcomes; survivorship inference limited
Osawa 2019 [114]	Japan; single-center academic hospit	Retrospective CT-based matched	49 women of 55 patients (89% female	NA; no clinical f/u (preoperative CT-based	NA	NA	NA (revision outcomes not asse	NA	NA	NA	NA	Not assessed	Y; Crowe I–III	2010–2017; CT-based simulation using standardized cup placement (45° inclination, 20° anteversion); evaluated	Morphologic simulation study only; no intraoperative or postoperative

	al (Nagoya University Hospital)	case–control simulation study	); mean age at THA 56.6 years (range 27–80); ≤50 not isolated	simulation only)			ssed)						anatomic vs +10 mm elevated hip center; implant brand, bearing surface, head size, and dual mobility not assessed	clinical outcomes; findings relate to acetabular bone stock and socket coverage rather than survivorship or instability
Osawa 2016 [113]	Japan; single tertiary academic center (Nagoya University Graduate School of	Retrospective matched case–control cohort study	Osteotomy group 48 patients (52 hips), 46 women (96%); control group 96 patients (104 hips),	Mean 5.4 years (range 1–14 years)	No Kaplan–Meier or competing-risk analysis; crude revision reporting	Final f/u (~5 years)	0/52 hips (0%)	0/104 hips (0%)	Not estimable (no revision events)	0/52 (0%); 1 dislocation without revision	0/104 (0%); 1 dislocation without revision	None; no infection, fracture, or aseptic loosening requiring revision	Y; Crowe I–II hip dysplasia; prior PAO types ERAO and RAO reported	2000–2014; posterior approach; cementless sockets predominantly (Trident HA, TriAD HA, Secur-Fit AD); some cemented all-polyethylene cups; ceramic-on-ceramic, ceramic-on-polyethylene, and metal-on-polyethylene bearings;	Revision defined as component exchange; prior PAO cohort only; relatively short f/u for survivorship; no Crowe III–IV hips; revision risk likely underestimated due to midterm f/u

	Medicine)		94 women; mean age at THA 56.5 years (range 44–74); ≤50 not isolated											femoral head sizes not explicitly listed; no dual mobility	
Ors 2022 [104]	Turkey; single private tertiary orthopedic hospital	Retrospective cohort study (prospectively collected data, retrospectively	91 patients (127 hips); 81 women (89%); mean age 43.0 ± 13.8 years; majority ≤50	Mean 8.4 years (100.6 months); range 6–10.4 years (72–125 months)	Kaplan–Meier survivorship analysis	10-year survivorship	7/127 hips (5.5%) revised for any reason	NA (DDH-only cohort)	NA	2/127 hips (1.6%) required revision for recurrent instability or liner-related instability	NA	Aseptic loosening (acetabular 3 hips; femoral 1 hip), ceramic liner fracture (1 hip), infection requiring debridement without component removal (3 hips;	Y; Crowe type IV only	2011–2015; posterolateral approach; cementless Wagner cone femoral stem with Trilogy IT acetabular shell; ceramic bearing; femoral head sizes 28 mm (76 hips) and 32 mm (51 hips); no dual mobility	Revision defined as exchange or removal of femoral or acetabular component; no comparator group; high intraoperative femoral crack rate; DDH severity homogeneous (Crowe IV

		analyzed)									revision not counted), femoral nonunion after fracture (1 hip)			only); mid-term f/u
Mu 2016 [105]	China; First Affiliated Hospital of Xinjiang Medical University	Retrospective single-center cohort study	58 patients (71 hips); 53 women (91%); mean age 35.8 years (range 16–56); majority ≤50	Mean 70.5 months (range 39–98 months)	Kaplan–Meier survivorship analysis with revision for any reason as endpoint	Survivorship reported at 98 months (~8 years)	7/71 hips (9.9%)	NA (DDH-only cohort)	NA	1/71 hips (1.4%)	NA	Severe polyethylene wear (4 hips), acetabular loosening (1 hip), metal cup breakage (1 hip), dislocation related to liner wear (1 hip)	Y; Crowe IV / Hartofilakidis type III only	1999–2009; posterolateral approach; cementless Zweymüller BICON-PLUS threaded acetabular cup and SL-PLUS femoral stem; polyethylene liner; femoral head sizes 22 mm or 28 mm; no dual mobility; transverse subtrochanteric shortening osteotomy in 56 hips	Revision defined as exchange or removal of any component; high complication rate related to early implant design; no comparator group; mid-term f/u; polyethylene wear prominent due to non-HXLPE liners

Mortazavi 2022 [18]	Iran; Tehran University of Medical Sciences, Imam Khomeini Hospital Complex	Retrospective single-center cohort study	DDH cohort 143 patients (171 hips), 127 women (88.8%); mean age 42.2 years (range 25–71); majority ≤50	Mean 40.4 ± 18.2 months (range 6–59 months)	No Kaplan–Meier or competing-risk analysis; crude event rates and logistic regression	Short-term to mid-term; f/u to ~5 years	Not explicitly reported; revisions not primary endpoint	Not explicitly reported	NR for revision	Not clearly specified; instability reported as dislocation events rather than revision indication	NR	Not detailed; study focused on dislocation rather than revision etiology	Y; Crowe classification I–IV	2014–2019; direct anterior approach; hemispherical uncemented cups (Continuum, Trilogy, Trident, Pinnacle); stems included Fitmore, Wagner SL, Wagner Cone, M/L Taper, Accolade, Corail, OmniFit; neutral liners only; femoral head size <32 mm associated with instability; no dual-mobility cups	Revision not primary outcome; instability assessed clinically; limited f/u; lack of implant survivorship curves; results primarily inform instability risk rather than long-term revision
Minoda 2006 [115]	Japan; single-center academic hospit	Retrospective matched coho	8 patients (10 hips) in Chiari group and 17	Mean 3.0 years; Chiari group 2.7 ± 1.1 years	NR; no Kaplan–Meier or competing-	Final f/u (~3 years)	0/10 hips (0%)	0/20 hips (0%)	Not estimable (no revision events)	0/10 hips (0%)	0/20 hips (0%)	None; no acetabular or femoral component loosening, migration, or osteolysis	Y; Crowe classification I–III (no Crowe IV)	1998–2003; posterolateral approach; cementless acetabular and femoral components; bulk autogenous	Revision defined as component loosening or failure; short f/u; small sample size; study focused

	al (Suita Municipal Hospital, Osaka)	rt study	patients (20 hips) in control group; all female; mean age 55.4 years (Chiari) and 56.3 years (control); ≤50 not isolated	(range 2–5), control group 3.1 ± 1.1 years (range 2–5)	risk analysis									femoral-head bone graft used in 9/10 Chiari hips and 18/20 control hips; prostheses included Stryker Omnifit and Anca-Fit; head size not specified; no dual mobility	primarily on biomechanical and radiographic outcomes rather than survivorship
Matsushita 2022 [120]	Japan; single academic institution (The	Retrospective cohort with Kapl	220 hips (201 hips available for long-term	Mean 11.4 years (137 months); range 5–14.6	Kaplan–Meier survival analysis	Up to 14 years (168 months); survivorship curves displaye	2 revisions among 220 hips	NA (DDH-only cohort)	NR (no comparator group)	0 revisions for instability	NA	Trunnionosis with adverse reaction to metal debris (1 hip); polyethylene liner	Y; Crowe classification (Type I–IV)	Cementless modular S-ROM-A femoral stem; cobalt-chromium heads; conventional and highly	Revision defined as any reoperation requiring component exchange; implant

	Jikei University School of Medicine)	an–Meier survivors hip analysis	f/u); majority female; age at surgery mean 62 years (range 35–84); women ≤50 not separately reported	years (60–175 months)		d to 168 months	(~1 %)					dissociation (1 hip)		cross-linked polyethylene liners; no dual-mobility bearings; stem anteversion adjusted intraoperatively	failure also analyzed as secondary endpoint; acetabular component heterogeneity limits inference; DDH-only cohort precludes comparative risk estimates
Liu 2021 [108]	China; single tertiary academic center (Xinqiao	Retrospective observational comparative coho	DAA cohort 22 patients (23 hips), 18 women (81.8	DAA mean 28.5 months (range 26–45 months); PLA	No Kaplan–Meier or competing-risk analysis;	Short-term f/u only (~2–3 years)	DAA 0/23 hips (0%); PLA 2/50 hips	NA (all patients DDH)	NA (approach comparison within DDH only)	0/73 hips overall (0%)	NA	Aseptic loosening (acetabular loosening at 8 months; femoral stem loosening due to osteotomy nonunion at	Y; Crowe III–IV only	2016–2018; cementless acetabular components placed at true hip center; frequent use of transverse subtrochanteric shortening	Revision defined as reoperation requiring component exchange; short f/u limits survivorship inference;

	Hospital, Army Medical University, Chongqing)	rt study (direct anterior approach vs posterolateral approach)	%), mean age 42.0 ± 13.6 years; PLA cohort 47 patients (50 hips), 33 women (70.2 %), mean age 47.8 ± 17.6 years; majority ≤50	mean 39.0 months (range 26–48 months)	crude revision reporting		(4.0 %)					14 months; both in PLA cohort)		osteotomy; bearing surfaces predominantly ceramic-on-ceramic and ceramic-on-polyethylene; femoral head sizes mainly 28 mm and 32 mm; no dual-mobility constructs	DDH-only cohort; comparison reflects surgical approach rather than diagnosis-based risk
Lari 2025 [127]	Kuwait, United Kingdom, Unite	Retrospective multicenter coho	37 patients (49 hips); 29 women	Mean 7.8 years (range 3–12 years)	Kaplan–Meier survivorship with revisi	Up to 12 years (survivorship stable	1/49 hips (2.0 %)	NA	NA	0/49 hips (0%)	NA	Osteotomy nonunion with over-lengthening requiring femoral stem	Y; Crowe type IV only	2013–2022; predominantly cementless cups; head sizes 22–36 mm; ceramic-on-polyethylene	Revision defined as any reoperation requiring component

	d Arab Emirates, Egypt; multicenter institutional databases	rt with Kaplan–Meier survivorship analysis	(78.4 %); mean age 35 ± 11.9 years; majority ≤50		on for any cause as endpoint	from 1 to 12 years)						revision (1 hip)		most common; limited dual mobility; tapered and conical stems; frequent subtrochanteric shortening osteotomy	exchange; no non-DDH comparator; heterogeneity in implants and surgical approach; complication rate relatively high but instability absent
Kristoffersson 2021 [121]	Sweden; Umeå University Hospital institutional arthroplasty cohort	Retrospective observational radiographic study	50 patients (56 hips); 27 women; age range 23–68 years; women ≤50 not isolated	Radiographic f/u 1–3 days postoperatively; no longitudinal clinical f/u for revision	NR	NA	NR	NA	NA	NA	NA	NA	Y; Crowe and Hartofilakidis classification systems	2008–2015; cementless acetabular and femoral components; multiple cup and stem designs; head size and bearing surface not reported; no dual-mobility constructs described	Revision outcomes not assessed; study focused exclusively on preoperative templating accuracy and radiographic reconstruction; limits inclusion in survivorship synthesis but relevant to technical

															planning in DDH
Kim 2023 [29]	Republic of Korea; single tertiary academic center	Retrospective cohort study	Women not stratified by age ≤50; total cohort 126 females / 172 hips	Mean f/u 2 years (minimum ≥2 years)	Crude event reporting (no Kaplan–Meier or competing-risk analysis)	2-year f/u only	0 revisions reported	NA	NR	0 revisions (no intraprosthetic dissociation or revision for dislocation)	NA	None observed during f/u	Y; Crowe classification (I–IV)	2015–2020 era; dual-mobility vs fixed-bearing cups; vitamin E-infused highly cross-linked polyethylene; femoral heads 28–36 mm	Revision defined as any reoperation requiring component exchange; short f/u limits survivorship inference; no non-DDH control; instability outcomes hip-based rather than patient-based
Kawasaki 2017 [97]	Japan; Aichi Kosei ren Konan Kosei Hospi	Prospective observational MRI-base	50 patients (50 hips); predominantly female;	12 months postoperative f/u only	NR; no Kaplan–Meier or competing-risk	12 months only	NR	NA	NA	NR	NA	NR	Y; Crowe classification (Group I, II, III)	2013–2014 era; cementless G7 acetabular component; Taperloc Complete Microplasty stem; direct anterior	Revision and survivorship outcomes not assessed; study objective limited to MRI-detected muscle

	tal / Nagoya University–affiliated institutions	d cohort study	women ≤50 not stratified		analysis									approach; bearing details not specified; no dual-mobility constructs	damage and fatty atrophy; inclusion appropriate for technical DDH literature but not for outcome synthesis
Kaiser 2022 [88]	Switzerland; Balgrist University Hospital institutional database	Retrospective cohort study with clinical and radiographic f/u	24 patients (29 hips); 17 women; mean age 43 years; women ≤50 not separately stratified	Mean f/u 35 months (range 12–137 months)	Crude event reporting; no Kaplan–Meier or competing-risk analysis	Final f/u only (up to ~11 years maximum)	1/29 hips (3.4 %)	NA	NR	0/29 hips (0%)	NA	Polyethylene liner exchange for progressive acetabular osteolysis (1 hip); no acetabular loosening; no graft failure	Y; Hartofilakidis classification (Type A and B)	2006–2018 era; cementless cups with bulk femoral head autograft; predominantly 28 mm heads; polyethylene liners; rare dual-mobility use; direct anterior approach with fluoroscopic guidance	Revision defined as reoperation with component exchange; short- to mid-term f/u; DDH-only cohort; absence of survivorship curves; results limited to mild-to-moderate dysplasia

Ikezaki 2024 [116]	Japan; Kyoto University Hospital	Retrospective cohort study with propensity-score matched control	Age not stratified ≤50; 40 women / 46 hips in shelf group; 39 women / 46 hips in control group	Shelf group mean 9.2 ± 6.6 years; control group mean 6.0 ± 3.7 years	Crude event reporting; no Kaplan–Meier or competing-risk analysis	Final f/u only (mean ~6–9 years)	0/46 hips (0%)	0/46 hips (0%)	NR (no revision events)	0/46 hips (0%)	0/46 hips (0%)	None observed; no infection, aseptic loosening, or implant failure reported	Y; Crowe classification (Crowe I only)	1997–2020; cemented, cementless, and hybrid THA; fixed-bearing acetabular components; frequent autologous bone grafting; anatomic hip center placement	Revision defined as any reoperation requiring component exchange; short- to mid-term f/u; limited generalizability to severe DDH (Crowe II–IV excluded); outcomes reported at hip level rather than patient level
Holzapfel 2012 [119]	Germany; institutional arthroplasty databases (Wür	Retrospective matched cohort study	37 patients (40 hips) cranial socket group; 35 patient	Cranial socket group mean 5.6 years (range 26–	Crude event reporting; no Kaplan–Meier or	Final f/u only (mid-term, ~5–7 years)	Cranial socket group: 0/40 hips (0%)	NA	NR	Cranial socket group: 0/40 hips (0%)<br>Control group:	NA	None; no aseptic or septic loosening in either group	Y; Hartofilakidis classification (type II only)	1998–2008; cementless press-fit acetabular components; UHMWPE liners; femoral heads 28–36 mm; no dual-	Revision defined as component exchange; mid-term f/u only; DDH-only cohort; outcomes hip-based

	zburg and Munich centers)		s (40 hips) control group; women ≤50 not isolated	133 months) Control group mean 6.9 years (range 30–151 months)	competing-risk analysis		Control group: 1/40 hips (2.5%) revised for recurrent dislocation			1/40 hips (2.5%)				mobility; cranial socket vs hemispherical cup with bulk autograft	rather than patient-based; limited generalizability to severe DDH or modern constructs
Gharanizadeh 2025 [99]	Iran; institutional arthroplasty databases from two referral centers	Retrospective comparative cohort study	134 patients (134 hips); female predominance in DDH group; age ≤50 not separately	Minimum f/u 3 months; longer-term survivorship not assessed	Crude event reporting; no Kaplan–Meier or competing-risk analysis	90-day postoperative outcomes only	2/47 hips (4.3%)	3/87 hips (3.4%)	NR for revision outcomes	1/47 hips (2.1%)	0/87 hips (0%)	Periprosthetic fracture (DDH and non-DDH); irrigation and debridement for early infection (non-DDH); sciatic nerve exploration (non-DDH)	Y; Crowe classification (Crowe III and IV in DDH group)	2021 era; mixed cemented/cementless THA; fixed-bearing liners; no dual-mobility use; frequent femoral shortening osteotomy and acetabular grafting in DDH hips	Revision defined as any reoperation; short-term f/u limits survivorship inference; study designed for VTE outcomes rather than instability or implant

			reported												survival; DDH and non-DDH groups differed substantially in baseline characteristics
Galea 2018 [98]	United States and Japan; single tertiary academic center (Massachusetts General Hospital collab	Prospective cohort study	Women ≤50 not stratified; 114/123 hips female (92.7%)	Mean 13.8 years (range 13.0–15.5 years)	Crude survivorship; revision-free survival percentage (no Kaplan–Meier or competing-risk analysis)	13-year f/u	1/112 hips (0.9%) revised (femoral aseptic loosening)	0/11 hips (0%)	NR	0/112 hips (0%)	0/11 hips (0%)	Femoral aseptic loosening (DDH only); no osteolysis, no polyethylene-related revisions	Y; Crowe classification (I–IV)	2000–2002 era; cementless cups; highly cross-linked polyethylene liners; 26 mm ceramic heads; no dual-mobility constructs	Revision defined as component exchange surgery; outcomes hip-based; very low event rates; small non-DDH comparator; limited instability conclusions due to zero-event outcome

	oration)														
Fukushi 2018 [110]	Japan; Kyushu University Hospital institutional cohort	Retrospective observational cohort study	100 patients (100 hips); 86 women; women n ≤50 not separately stratified	12 months postoperative f/u only	NR; no survivorship analysis is performed	12 months only	NR	NA	NA	NR	None reported	NR	Y; Crowe classification (Crowe I–III)	2012–2016 era; cementless cups and stems; femoral head sizes 26–32 mm; bearing surface not specified; no dual-mobility constructs	Revision and survivorship outcomes not evaluated; study focused on abductor muscle strength recovery and hip center position; appropriate for technical DDH literature but not for instability or revision synthesis
Fahlbusch 2023 [66]	Germany; Helios ENDO-Klinik Hamb	Retrospective single-center coho	91 women (83%) among 126 patients; wome	Mean 23 ± 1.3 years (range 21–25); minimum 21	Kaplan–Meier survivorship analysis	10 years, 20 years, final f/u (~23 years)	22 revisions / 121 hips (18 %)	NA	NR	1 revision related to dislocation (cup and taper exchange due to	NA	Aseptic cup loosening (8%); aseptic loosening of both cup and stem (8%); periprosthetic infection	Y; Crowe classification (I–IV)	Late 1990s implant era; predominantly cemented polyethylene cups; ceramic and metal femoral heads;	Revision defined as surgery requiring component exchange; DDH-only cohort without non-

	urg and University Medical Center Hamburg-Eppendorf	rt study	n ≤50 not separately stratified	years required for inclusion						loosening) (~0.8%)		(1%); periprosthetic fracture (1%)		no dual-mobility constructs	DDH control; long f/u strengthens survivorship estimates but reflects outdated implant designs; instability and revision outcomes reported per hip rather than per patient
Engesaeter 2012 [25]	Denmark, Norway, Sweden; national arthroplasty registries (NARA)	Population-based registry cohort with Kaplan–Meier and Cox	Total cohort 370,630 THAs; DDH subgroup 12,068 THAs; women ≤50 not separately	Mean f/u ~6.4 years for DDH; registry f/u up to 14 years	Kaplan–Meier survivorship with Cox proportional hazards regression; comp	6 months, 10 years, up to 14 years	706/12,068 THAs (5.9%)	11,214/288,435 THAs (3.9%)	Adjusted RR for any revision: 1.09 (95% CI 1.00–1.18); not significant after full adjustm	138/12,068 (1.1%)	2,485/288,435 (0.86%)	Aseptic loosening; infection; periprosthetic fracture (reported but not stratified by DDH severity)	N; DDH recorded as dichotomous registry diagnosis only	1995–2009; mix of cemented, uncemented, hybrid, and reverse hybrid fixation; bearing details not available	Revision defined as exchange or removal of any component; bilateral hips included (~20%); dislocations captured only if revised; inability to stratify by DDH severity

		regression analyses	reported		eting risk assessed and negligible				ent; early revision (<6 months) RR 1.3 (95% CI 1.1–1.6)						is major limitation
Di Martino 2021 [122]	Italy; Emilia-Romagna regional arthroplasty registry (RIPO)	Population-based retrospective registry cohort with Kaplan–Meier and Cox regression	5,761 THAs; female predominance (~75–80%); women ≤50 not separately stratified	Mean f/u varied by cohort (6.5–10.7 years); maximum f/u 17 years	Kaplan–Meier survivorship; Cox proportional hazards regression adjusted for age and sex	1, 3, 5, 7, 10, and 15 years	Overall revision rates ranged from 1.6% to 3.3% by stem cohort at mid- to long-	NA	NA	Approximately 0.2–0.6% depending on stem design; no significant difference between stem cohorts	NA	Cup aseptic loosening (notably higher in non-modular conical stems, 2.5%); stem aseptic loosening; periprosthetic fracture; infection	N; DDH recorded as registry diagnosis only (no Crowe or Hartofilakidis classification)	2000–2017; cementless THA; mixed ceramic-on-ceramic, ceramic-on-polyethylene, metal-on-polyethylene bearings; head sizes both <36 mm and ≥36 mm; no dual-mobility reporting	Revision defined as exchange or removal of any component; bilateral hips included; instability captured only if revised; inability to stratify by DDH severity is a major limitation

		analyses					term f/u								
DeNovio 2025 [124]	United States; Mayo Clinic institutional total joint registry	Retrospective cohort study with long-term f/u	Women ≤50 not stratified; 24 patients (28 hips), 83% female	Mean f/u 18 years; range 12–28 years	Kaplan–Meier survivorship analysis	1, 2, 5 years and every 5 years thereafter; survivorship reported to 20 years	5 revisions among 28 hips; 20-year survivorship free of any revision 79%	NA	NR	0 revisions performed for instability; dislocations treated with closed or open reduction only	NA	Aseptic loosening of femoral stem (2 hips); acetabular aseptic loosening (1 hip); femoral stem fracture (1 hip); polyethylene liner dissociation (1 hip)	Y; Crowe classification (Crowe IV only)	1992–2005 era; cementless porous-coated stems and cups; highly cross-linked polyethylene liners; no dual-mobility constructs	Revision defined as any procedure involving removal or exchange of an implant component; reoperations without component exchange excluded; small cohort; DDH-only population limits comparative inference; outcomes reported on a hip-based basis
Colo 2016 [90]	The Netherlands	Retrospective coho	22 patients (28 hips);	Mean f/u 20 years (range	Competing-risk analys	15 years and 20 years	3/28 hips (10.7%	NA	NR	0/28 hips (0%)	NA	Aseptic cup loosening (2 hips at 12 and 26	Y; Crowe classification (I–IV) and	1984–1995 era; cemented THA; impaction bone grafting with	Revision defined as any surgery

	; Radboud University Medical Center institutional cohort	rt study with competing-risk analysis	17 women; women ≤50 partially represented but not separately stratified	16–29 years); minimum f/u 15 years	is accounting for death as competing event		crude); cumulative incidence of revision for any reason 7% at 15 and 20 years					years); cup revision for sciatic nerve palsy related to high hip center (1 hip at 2 years)	Eftekhar classification (A–C)	cemented cups; polyethylene liners; no modern bearings or dual-mobility constructs	requiring component exchange; deaths treated as competing risk; small cohort; no comparator group; instability outcomes absent but not primary endpoint
Clark 2025 [95]	United States; Mayo Clinic Joint Replacement	Retrospective propensity-matched coho	Women ≤50 not separately reported; total cohort 190 hips	Mean f/u 5.2 ± 2.8 years (DDH) and 5.1 ± 2.8 years (OA	Kaplan–Meier survivorship analysis for freedom from	Up to approximately 8–10 years; primary reporting at final f/u (~5 years)	2/38 hips (5.3%)	2/152 hips (1.3%)	NR; no adjusted hazard ratio provided; difference not statistically signific	0 revisions for instability	0 revisions for instability	Leg length discrepancy (DDH and OA); femoral stem loosening (DDH); deep infection requiring irrigation and	Y; Crowe classification (I–IV)	2010–2022 era; fixed-bearing acetabular components; polyethylene liners; femoral heads predominantly 32–36 mm; no	Revision defined as any reoperation requiring component exchange; instability outcomes hip-based rather than

	Registry	rt study	(38 DDH, 152 OA); female proportion ~69% in both cohorts		revision				ant (P = 0.179)			debridement (OA)		dual-mobility constructs	patient-based; predominance of mild dysplasia limits generalizability to Crowe III–IV; retrospective design with registry dependence
Chen 2018 [23]	China; West China Hospital, Sichuan University	Prospective cohort study with biomechanical f/u	45 DDH patients (39 women); women ≤50 not separately stratified	Mean f/u approximately 5 years (57 months)	NR; no survivorship analysis is performed	5-year f/u only	NR	NA	NA	NR	NA	NR	Y; Crowe classification (Crowe II–III)	Early–mid 2010s era; cementless ceramic-on-ceramic THA; no dual-mobility constructs; posterolateral approach	Revision and instability outcomes not assessed; study designed for gait analysis and limb-length discrepancy effects; appropriate for biomechanical context but excluded from

															instability or survivorship synthesis
Chai 2022 [128]	China; single high-volume academic center (PLA General Hospital, Beijing)	Retrospective propensity score–matched comparative cohort study (1:1); robotic-assisted versus conv	DDH only; matched cohort of 54 hips (27 robotic-assisted, 27 conventional); 100% female in matched groups; mean age 43.0 ± 8.9	Minimum 2 years; clinical outcomes assessed at 1 and 2 years	NR; no formal survivorship analysis is reported	1 and 2 years	NR	NA	NA; no non-DDH comparator	NR; no dislocations reported	NA	NR	Y; Crowe III–IV only	January 2017–August 2019; cementless THA; posterolateral approach; Mako robotic-assisted versus conventional/manual THA; Trident cups in robotic group versus Pinnacle/Combi cup in conventional group; modular S-ROM femoral stem; true acetabulum reconstruction when possible; frequent subtrochanteric shortening	Study primarily evaluated acetabular cup-positioning accuracy and short-term clinical outcomes rather than implant survivorship; robotic THA achieved greater Lewinnek/Callanan safe-zone placement; no dislocations or nerve injuries were reported; formal all-

		entional THA	years (robotic) and 44.6 ± 9.5 years (conventional); women ≤50 not separately isolated; non-DDH = NA											osteotomy; head size NR; no dual mobility	cause or cause-specific revision outcomes were not reported
Caylak 2021 [125]	Turkey; single tertiary orthopedic center	Retrospective cohort study	43 women / 67 hips (age not stratified specifically	Mean 131.9 months (range 120–148 months)	Kaplan–Meier survivorship analysis	10-year f/u	4/67 hips (6.0 %)	NA	NR	0 revisions for instability; no revision performed specifically for	NA	Periprosthetic femoral fracture (1); periprosthetic joint infection (1); nonunion of subtrochanteric osteotomy	Y; Crowe classification (type IV only)	2008–2011 era; cementless acetabular and femoral components; ceramic-on-ceramic bearings; femoral heads 22–32 mm	Revision defined as any surgery requiring component exchange; long-term f/u strengthens survivorship data; no control

			≤50, but majority under 50)							dislocation		(1); aseptic femoral loosening (1)			group; high surgical complexity limits generalizability; instability outcomes reported per hip rather than per patient
Castagnini 2022 [123]	Italy; Emilia-Romagna Arthroplasty Registry (RIPO)	Population-based registry cohort with Kaplan–Meier survivorship and Cox	6,429 THAs; female predominance; women ≤50 not separately stratified	Mean f/u 9.0 years (ST) and 9.3 years (DT); range 0–19 years	Kaplan–Meier survivorship analysis; multivariable Cox regression adjusted for age (≥65 years), sex,	1, 3, 5, 7, 10, 15, and 17 years	ST cohort: 196/3,642 (5.4%); DT cohort: 115/2,787 (4.1%)	NA	NA	ST: approximately 0.7%; DT: approximately 0.6%	NA	Cup aseptic loosening (higher in ST); stem aseptic loosening (similar); implant breakage (higher in DT, 0.6%); periprosthetic infection; no revisions for metallosis	N; DDH recorded as registry diagnosis without Crowe or Hartofilakidis classification	2000–2018 era; cementless THA; mixed bearings; femoral head sizes commonly ≤28 mm; no dual-mobility constructs	Revision defined as exchange or removal of any component; bilateral hips included; registry cannot capture non-revised instability; demographic imbalance between ST and DT cohorts may bias results;

		regression			and head size (≤28 mm)										study overlaps conceptually with Di Martino 2021 but analyzes modularity specifically
Bus 2024 [94]	Netherlands; Dutch Arthroplasty Register (LROI)	Nationwide population-based registry cohort with competing-risk and Cox regression	24,263 THAs in patients <55 years; DDH n=3,032; OA n=20,645; women ≤50 not separately stratified	Minimum f/u 2 years; outcomes reported up to 10 years; mean/median f/u not explicitly stated	Competing-risk cumulative incidence (death as competing event); multivariable Cox proportional hazar	2, 5, and 10 years	10-year cumulative incidence 6.0% (95% CI 4.9–7.2)	OA: 10-year cumulative incidence 6.0% (95% CI 5.6–6.5)	Adjusted HR for overall revision (DDH vs OA): 1.0 (95% CI 0.8–1.4)	Not separately reported	Not separately reported	Aseptic cup failure (10-year cumulative incidence 3.4% DDH vs 3.4% OA); infection and other causes included within all-cause revision but not stratified by diagnosis	N; DDH recorded as registry diagnosis without Crowe or Hartofilakidis classification	2007–2019 era; predominantly uncemented cups; mixed bearings (ceramic-on-polyethylene most common); head sizes commonly 32 mm	Revision defined as exchange or removal of ≥1 component; diagnosis not radiographically validated; instability under-ascertained due to registry design; possible misclassification between mild DDH and OA

		analyses			ds models									
Boyle 2012 [93]	New Zealand; New Zealand National Joint Registry	Nationwide population-based registry cohort with Cox proportional hazards regression	Total cohort 41,794 THAs; DDH subgroup 1,205 THAs; women ≤50 not separately stratified	Maximum f/u 10 years; mean f/u not explicitly reported	Cox proportional hazards regression; crude revision rates per 100 component-years	Up to 10 years; early (6-month) outcomes reported separately	42/1,205 revisions; revision rate 0.79 per 100 component-years	1,031/40,589 revisions; revision rate 0.61 per 100 component-years	Adjusted HR for any revision: 1.08 (95% CI 0.75–1.55); not statistically significant	Reported as part of revision reasons; no significant difference vs OA; exact count not isolated	Reported as part of revision reasons; exact count not isolated	Aseptic loosening (acetabular and femoral); deep infection; pain; femoral fracture; dislocation (all without significant DDH vs OA difference)	N; DDH recorded as registry diagnosis only	1999–2008 era; mix of cemented, uncemented, and hybrid fixation; bearing and head size data unavailable; no dual-mobility reporting	Revision defined as exchange or removal of any component; registry cannot capture non-revised instability; severity of DDH unknown; functional outcomes reported at 6 months only; bilateral hips included
Biant 2009 [28]	Australia; Concord	Prospective singl	22 patients (28 hips);	Mean f/u 10 years; minim	Crude survivorship report	5 years and 10 years	3/28 hips	NA	NR	0/28 hips (0%)	NA	Acetabular wear and loosening (2 hips at 6 and	Y; Crowe classification (Crowe	Early–mid 2000s era; cementless modular S-ROM	Revision defined as any surgery requiring

	Repatriation General Hospital / University of New South Wales	e-center cohort study	17 women; women ≤50 not separately stratified but majority under 50	um 7 years; radiographic f/u to 10 years	ing; no Kaplan–Meier or competing-risk analysis		(10.7%)					15 years); polyethylene liner wear requiring exchange (1 hip at 7 years); no femoral stem revisions	III and IV only)	stem; polyethylene liners; femoral heads predominantly 28 mm; no dual-mobility constructs	component exchange; no patients lost to f/u; outcomes reported per hip; small cohort; absence of dislocation despite small head sizes may reflect meticulous technique and limited generalizability
Beringer 2019 [22]	Germany; BG Trauma Center Murnau / Garmisch-	Retrospective single-center cohort study	39 women / 61 hips; women ≤50 not separately stratified	Mean f/u 38 months (range 10–66 months)	Crude event reporting; no Kaplan–Meier or competing-	Approximately 3-year f/u only	1/61 hips (1.6%)	NA	NR	0/61 hips (0%)	NA	Aseptic acetabular loosening (1 hip in Crowe IV patient with prior childhood hip surgeries)	Y; Crowe classification (II–IV)	2010–2014 era; cementless cups and stems; polyethylene liners; femoral heads 28–36 mm; no dual-mobility constructs	Revision defined as surgery requiring component exchange; no dislocations, impingement, or neurologic complications observed;

	Partenkirchen Medical Center				risk analysis									outcomes reported per hip; limited f/u precludes long-term survivorship conclusions
Benad 2022 [117]	France; single tertiary academic orthopedic center	Retrospective case–control cohort study	Women ≤50 not stratified separately; total cohort 105 patients (124 hips)	Mean f/u 13 ± 6 years (range 5–25 years)	Kaplan–Meier survivorship analysis with log-rank comparison	15-year survivorship	Shelf group: approximately 11% revised by 15 years (survivorship 89%)	Control group: approximately 17% revised by 15 years (survivorship 83%)	No hazard ratio or risk ratio reported; log-rank p = 0.566 (not significant)	1 revision for chronic posterior instability (≈1.6%)	0 revisions for instability	Aseptic acetabular loosening (more frequent in control group), aseptic femoral loosening, painful impingement, periprosthetic fracture, deep infection	N; DDH defined radiographically using VCA and VCE angles; Crowe classification not used	1990–2010 era; mixed bearings; no dual-mobility constructs; occasional acetabular reinforcement rings and femoral head autografts	Revision defined as component exchange or removal; isolated liner or head exchange not counted as revision; older implant designs limit modern applicability; instability outcomes hip-based; no non-DDH population; shelf and control groups both

																represent dysplastic hips
Ashraf 2014 [96]	United States; Mayo Clinic institutional total joint registry and cost database	Matched retrospective cohort study	354 DDH patients and 1,029 matched osteoarthritis controls; women ≤50 not separately stratified	Minimum 2-year clinical f/u for inclusion; outcomes primarily reported for hospitalization and 90-day period	NR; no Kaplan–Meier or competing-risk survivorship analysis	90-day postoperative period only	NR	NR	NA	NR	NR	Not assessed	Y; Crowe classification (0–IV)	2000–2008 era; mixed fixation and implant designs; no reporting of bearing type, head size, or dual-mobility use		Study was designed to evaluate hospital cost, length of stay, and 90-day complications rather than revision or instability; long-term survivorship not assessed; possible occult DDH in control group; appropriate for economic context but excluded from instability and revision synthesis

Asai 2025 [118]	Japan; Nagoya University Graduate School of Medicine	Retrospective propensity-matched cohort study with Kaplan–Meier survivorship analysis	PAO group: 46 patients (50 hips); control group: 48 patients (50 hips); women ≤50 not separately stratified	PAO group: mean 13.0 years (range 10–21); control group: mean 13.6 years (range 10–22)	Kaplan–Meier survival analysis with revision surgery and radiolucent line appearance as endpoints; log-rank testing	10-year survivorship	PAO group: 2/50 hips (4.0%)	Control group: 2/50 hips (4.0%)	NR; no hazard ratio provided; log-rank test showed no significant difference	0/50 hips (0%)	0/50 hips (0%)	Deep infection (PAO group); osteolysis (PAO and control groups); periprosthetic fracture (control group)	Y; Crowe classification (I–III), used for propensity matching	2000–2014 era; cementless cups and stems; polyethylene liners; no dual-mobility constructs; occasional acetabular bone grafting	Revision defined as any surgery requiring component exchange; prior osteotomy group represents selected DDH population progressing to THA; limited power for rare events; instability outcomes hip-based; implant selection not standardized

Abbreviations: f/u = follow-up; Y = Yes; N = No; NR= Not Reported; NA= Not Applicable

Note: The definition of "revision" varies across registries and cohorts; specify whether it includes component exchange only, any reoperation, or any implant removal/exchange. If cause-specific revision coding is incomplete or variable, denote "cause not reliably classified" and interpret estimates cautiously.
